# Importance of quorum sensing crosstalk in the brown alga *Saccharina latissima* epimicrobiome

**DOI:** 10.1016/j.isci.2024.109176

**Published:** 2024-02-09

**Authors:** Emilie Adouane, Camille Mercier, Jeanne Mamelle, Emma Willocquet, Laurent Intertaglia, Bertille Burgunter-Delamare, Catherine Leblanc, Sylvie Rousvoal, Raphaël Lami, Soizic Prado

**Affiliations:** 1 Muséum National d’Histoire Naturelle, Unité Molécules de Communication et Adaptation des Micro-Organismes MCAM, UMR 7245, CNRS, Sorbonne Université, 75005 Paris, France; 2 Sorbonne Université, CNRS, UAR 3579 Laboratoire de Biodiversité et Biotechnologies Microbiennes LBBM, Observatoire Océanologique, 66650 Banyuls-sur-Mer, France; 3 Sorbonne Université, CNRS, Bio2Mar, Observatoire Océanologique, 66650 Banyuls-sur-Mer, France; 4 Biologie Intégrative des Modèles Marins, LBI2M (Sorbonne Université/CNRS), Station Biologique de Roscoff (SBR), 29680 Roscoff, France

**Keywords:** Microbiology, Microbiofilms

## Abstract

Brown macroalgae are colonized by diverse microorganisms influencing the physiology of their host. However, cell-cell interactions within the surface microbiome (epimicrobiome) are largely unexplored, despite the significance of specific chemical mediators in maintaining host-microbiome homeostasis. In this study, by combining liquid chromatography coupled to mass spectrometry (LC-MS) analysis and bioassays, we demonstrated that the widely diverse fungal epimicrobiota of the brown alga *Saccharina latissima* can affect quorum sensing (QS), a type of cell-cell interaction, as well as bacterial biofilm formation. We also showed the ability of the bacterial epimicrobiota to form and inhibit biofilm growth, as well as to activate or inhibit QS pathways. Overall, we demonstrate that QS and anti-QS compounds produced by the epimicrobiota are key metabolites in these brown algal epimicrobiota communities and highlight the importance of exploring this epimicrobiome for the discovery of new bioactive compounds, including potentially anti-QS molecules with antifouling properties.

## Highlights

• The epimicrobiome of brown alga *S. latissima* shows high microbial and chemical diversity
• The algal epimicrobiome hosts intense and multidirectional cell-cell communication
• QS and anti-QS compounds are crucial for cell-cell communication in fungi and bacteria
• Algal epimicrobiome yields bioactive compounds, potential anti-QS antifouling agents

## Graphical abstract

**Figure:**
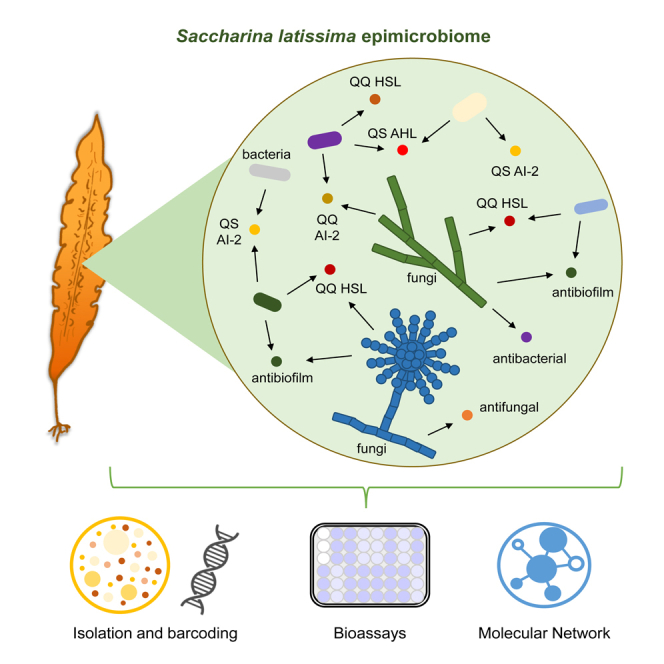

## Introduction

Brown macroalgae play a crucial role in temperate coastal ecosystems by serving as vital habitats and nutrient sources for many organisms, making a substantial contribution to local biodiversity.^1^^,^^2^ Currently, the macroalgae industry is undergoing notable expansion, particularly in sectors such as agriculture,^3^ aquaculture,^4^ pharmacology,^5^^,^^6^ and cosmetics.^7^^,^^8^ Additionally, brown algae might be valuable sources of energy and biofuel.^9^^,^^10^^,^^11^ Like many eukaryotes, brown macroalgae harbor a diverse range of microorganisms that exert a significant influence on their growth, morphogenesis, and nutrition.^12^^,^^13^^,^^14^^,^^15^ These multiple and specific interactions led therefore to define macroalgae and their associated microbiome as a “superorganism,” called the holobiont.^12^ These interactions are finely regulated by the production of chemical mediators that control the maintenance of host-microbiome homeostasis and thus contribute to the health status of the host.^16^^,^^17^^,^^18^^,^^19^

The surface of macroalgae is colonized by a microbial community, a biofilm-like epimicrobiome, known as a “second skin,”^15^ which is the site of chemical interactions and ecological processes. However, our understanding of the nature, diversity, and ecological roles of chemical signals within this biofilm remains incomplete. Bacteria within the epimicrobiota have been the subject of numerous studies.^19^^,^^20^^,^^21^^,^^22^^,^^23^^,^^24^ These bacteria have been found to produce antimicrobial and antifouling compounds, enabling algae to defend themselves against pathogens.^25^^,^^26^^,^^27^

Conversely, the role of fungi within the holobiont has been relatively understudied, with only a few studies specifically focusing on brown macroalgal endophytes.^28^^,^^29^ For instance, Vallet et al.^30^ demonstrated that fungal endophytic metabolites play a key role against protist pathogens in various brown algae including *Saccharina latissima* (Linnaeus) C.E. Lane, C. Mayes, Druehl et G.W. Saunders^31^. In addition, some endophytic fungi associated with brown macroalgae also display significant production of antibiotic, antifungal, antioxidant, and larvicidal compounds.^32^^,^^33^^,^^34^^,^^35^ Some authors have shown that epiphytic fungi are also able to synthesize secondary metabolites that contribute to algal health.^36^^,^^37^^,^^38^^,^^39^ However, the molecular characterization of interspecies interactions between bacteria and fungi within this complex network remains largely unexplored.

Quorum sensing (QS), a coordinated response of a microbial population based on the production of chemical signals, also known as autoinducers (AI), plays a key role in biofilm formation and maintenance.^40^^,^^41^^,^^42^ As cell density increases, AI accumulate until they reach a threshold that triggers the activation or repression of numerous genes regulating phenotypes such as adhesion, virulence, colonization, or even the motility of microorganisms.^43^^,^^44^^,^^45^ Various types of AI are already known. Some have been found in only one bacterial genus, whereas others, such as *N*-acyl homoserine lactone (acyl homoserine lactone [AHL]), type 1 (AI-1), or furanosyl borate diester, type 2 (AI-2), are present in various bacterial genera.^46^^,^^47^^,^^48^ Strikingly, quorum signals are not exclusively produced by bacteria. Fungi are also able to regulate their morphogenesis, pathogenicity, biofilm formation, and dispersal through QS mechanisms (QSM) involving compounds such as farnesol, phenylethanol, and tryptophol.^49^^,^^50^^,^^51^^,^^52^ Some of these compounds are involved in cross-kingdom signaling, enabling bacteria and fungi to regulate each other via specific compounds.^53^^,^^54^^,^^55^ Additionally, cross-kingdom signaling is also modulated by QS inhibitors, i.e., quorum quenchers.^56^^,^^57^ To date, halogenated furanones produced by the red macroalga *Delisea pulchra* are the best-known bacterial QS inhibitors and enable it to regulate bacterial colonization.^58^^,^^59^^,^^60^

Our group reported that bacterial and fungal endophytes associated with brown algae (*Ascophyllum nodosum*, *Pelvetia canaliculata*, *Laminaria digitata*, and *Saccharina latissima*) produce metabolites that interfere with bacterial AI-2 QS, a key cell-cell signaling system implicated in virulence and host colonization.^61^ However, the role of QS in regulating interactions between microorganisms associated with brown macroalgae remains poorly investigated especially on the surface of the algae, the site of complex biofilms.

This study aimed to investigate the diversity of molecular interactions between microorganisms within the *Saccharina latissima* epimicrobiome. We first isolated and identified the bacterial and fungal strains present in the algal epimicrobiome. Subsequently, the metabolomes of the fungal strains were analyzed by using LC-MS/MS. To explore the role of QS in these interactions, we evaluated the ability of fungi to inhibit QS and bacterial biofilm formation. Furthermore, we assessed the bacterial strains’ capacity to form and inhibit biofilms, as well as produce and inhibit QS compounds. Overall, our findings suggest that QS compounds, whether they are activators or inhibitors, produced by the cultivable microbiota of *S. latissima* play an essential role in the complex network of interactions within the algal holobiont.

## Results

### Cultivable fungal and bacterial diversity from *S. latissima* epimicrobiota

**Figure 1:**
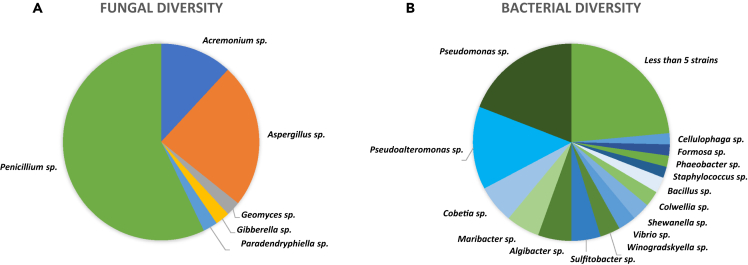
Fungal and bacterial cultivable diversity from the epimicrobiota of *S. latissima* (A) fungal diversity at the genus level of the 42 strains isolated (B) bacterial diversity at the genus level of the 272 strains isolated.

Fungal strains were isolated on two culture media (malt extract agar [MEA] 75% natural seawater [NSW] and potato dextrose agar [PDA] 75% NSW) to maximize cultivable diversity. Forty-two fungal strains were obtained (Table S1). Among the fungal strains, 100% were affiliated with Ascomycota (95% Sordariomycetes, 2% Dothideomycetes, and Leotimycetes). The cultivable fungal epimicrobiota contained 10 different species (Table S1), including many *Penicillium* sp. (57%), *Aspergillus* sp. (24%), and a few *Acremonium* sp. (12%). The singletons were *Geomyces pannorum*, *Gibberella intricans*, and *Paradendryphiella salina* (Figure 1A).

A total of 272 bacterial strains were isolated on marine agar (MA) from the epimicrobiota of *S. latissima* (Figure 1B; Table S2) and classified, based on their 16S rRNA gene sequences, into 4 phyla, 6 classes, 13 orders, 24 families, 48 genera with 16 taxonomically unique units. The most abundant phyla were Pseudomonadota (formerly known as Proteobacteria, 61%), Bacteroidota (Bacteroidetes, 24%), Bacillota (Firmicutes, 10%), and Actinomycetota (Actinobacteria, 5%). Among Pseudomonadota, Gammaproteobacteria were the most abundant (51%), compared with Alphaproteobacteria (8.8%). The Flavobacteriaceae family was the most represented one (23% of total sequences), including *Algibacter* sp. (5%), *Maribacter* sp. (5%), and *Winogradskyella* sp. (3%), as well as *Cellulophaga* sp., *Dokdonia* sp., and *Zobellia* sp. The Pseudomonadaceae and Pseudoalteromonas families were composed of *Pseudomonas* sp. (19%) and *Pseudoalteromonas* sp*.* (13%). Bacillaceae (7%) and Rhodobacteraceae (8%) families were also well-represented with the genera *Bacillus* sp. (2.5%), *Brevibacterium* sp., *Cytobacillus* sp*., Priesta* sp., *Sulfitobacter* sp. (4.7%), and *Phaeobacter* sp. Members of Halomonadaceae (6.25%), Shewanellaceae (3%), Colwelliaceae (2.5%) and Vibrionaceae (2.5%) families were also isolated. This high bacterial diversity was confirmed by the fact that 24% (33 strains) of the collection consisted of genera represented by less than 5 isolates, including 16 genera represented by only one isolate each.

### Diversity of metabolites involved in microbial interactions produced by fungal strains isolated from the epimicrobiota of *S. latissima*

**Figure 2:**
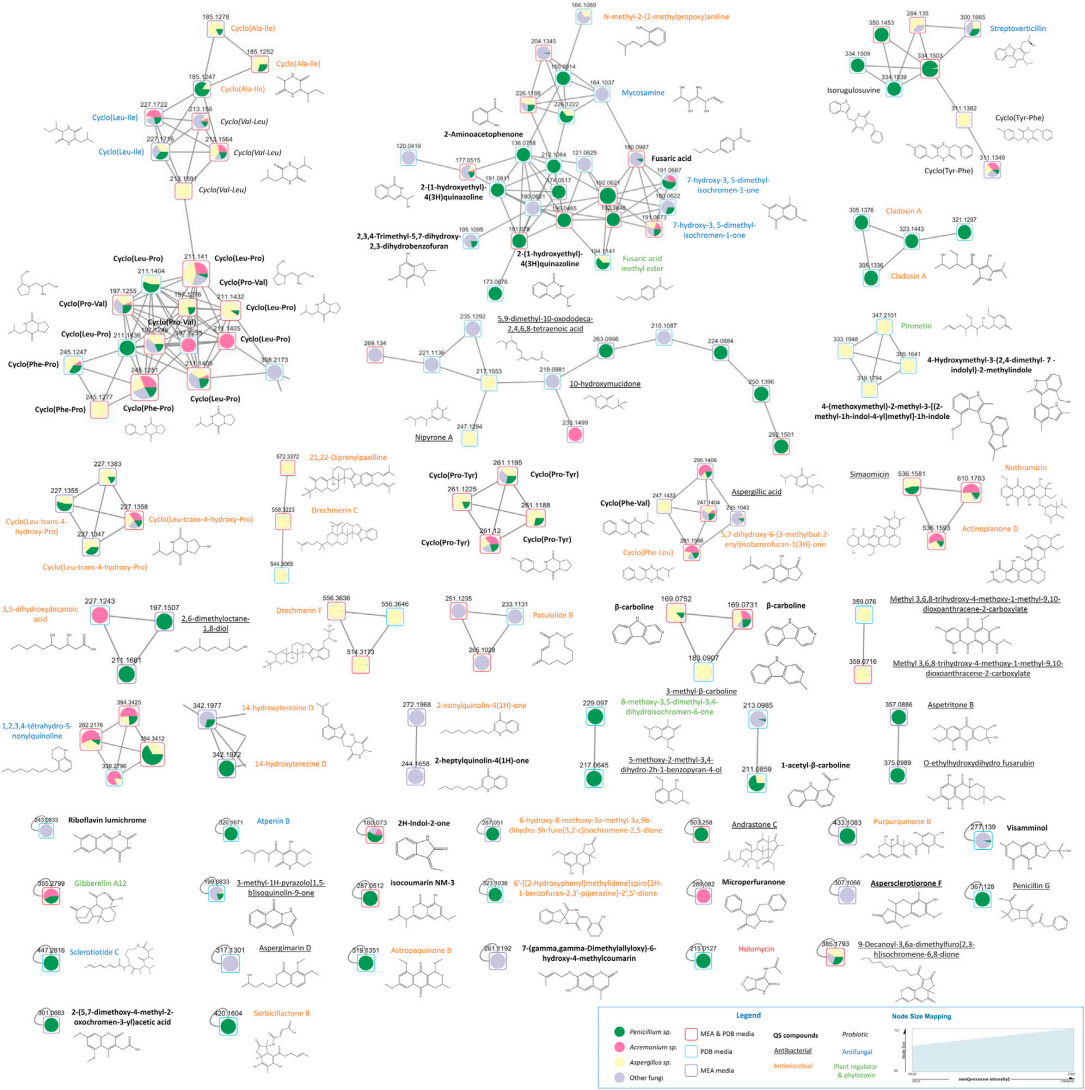
Metabolites putatively involved in microbial interactions produced by fungal strains isolated from the *S. latissima* epimicrobiota Molecular subnetworks obtained after extraction of 100 metabolites potentially involved in interactions between microorganisms. The complete network containing 573 metabolites is shown in Figure S1 and annotations are shown in Tables S3 and S4.

The metabolomes of 42 fungal strains isolated from the epimicrobiota of *S. latissima* grown in MEA and in potato dextrose broth (PDB) were analyzed by LC-MS/MS. After data processing, 1,442 features were obtained in MS (data not shown) and 573 in MS/MS (Table S3). The molecular network formed by the 573 metabolites is shown in Figure S1 and comprises amino acids (18.8%), numerous fatty acyls and derivatives (16.5%), terpenoids (4%), prenol lipids (3%), and indoles (3%) (Table S3). Among the putatively annotated metabolites (Table S3), those potentially involved in microbial interactions (17.4%, 99 metabolites) based on the literature (Table S4) have been grouped in Figure 2 in the form of a subnetwork.

Within this subnetwork, amino acids and derivatives were particularly predominant, with 39 putative compounds including 36 cyclodipeptides. The results also highlighted a wide range of compounds produced, including putative antibacterials (18), such as aspergillic acid,^62^ penicillin G,^63^ simaomicin,^64^ nothramicin,^65^ and 3-methyl-1H-pyrazolo[1,5-*b*]isoquinolin-9-one^66^ or O-ethylhydroxydihydrofusarubin.^67^ The fungal strains also produced putative antifungals (10), such as *cyclo*(Phe-Pro) described previously,^68^ streptoverticillin,^69^ and mycosamine,^70^ as well as more general antimicrobials (26), such as astropaquinone B,^71^ atpenin B,^72^ 7-hydroxy-3,5-dimethyl-isochromen-1-one,^73^ drechmerin C and F,^74^ cyclo(Ala-Ile)^75^ and cladosin A.^76^

QS-inhibiting compounds were also putatively annotated, such as *cyclo*(Leu-Pro), *cyclo*(Phe-Pro), *cyclo*(Phe-Val), *cyclo*(Pro-Val), *cyclo*(Val-Leu), and *cyclo*(Pro-Tyr).^77^^,^^78^^,^^79^^,^^80^^,^^81^^,^^82^ Indeed, cyclopeptides/diketopiperazines (12 detected) are frequently involved in QS inhibition mechanisms,^77^^,^^83^^,^^84^ as well as coumarins (8 compounds detected),^85^^,^^86^ beta-carboline (5)^87^ and butenolides (2).^88^^,^^89^ Other compounds involved in QS were putatively annotated, such as activators 2-aminoacetophone,^90^ riboflavin lumichrome,^91^ 2-heptylquinolin-4(1H)-one (HHQ)^92^ and an inhibitor, microperfuranone.^93^^,^^94^ Two algicides were also detected: *cyclo*(Leu-*trans*-4-hydroxy-Pro) and *N*-methyl-2-(2-methylpropoxy)aniline.^95^^,^^96^ Fungal compounds involved in chemical plant signaling were also identified such as the growth regulator pironetin,^97^ a compound involved in plant signaling, gibberellin A12^98^ and a root growth inhibitor, aspergimarin D.^99^

### Production of QS chemical inhibitors by cultivable fungal strains of the algal epimicrobiota

**Figure 3:**
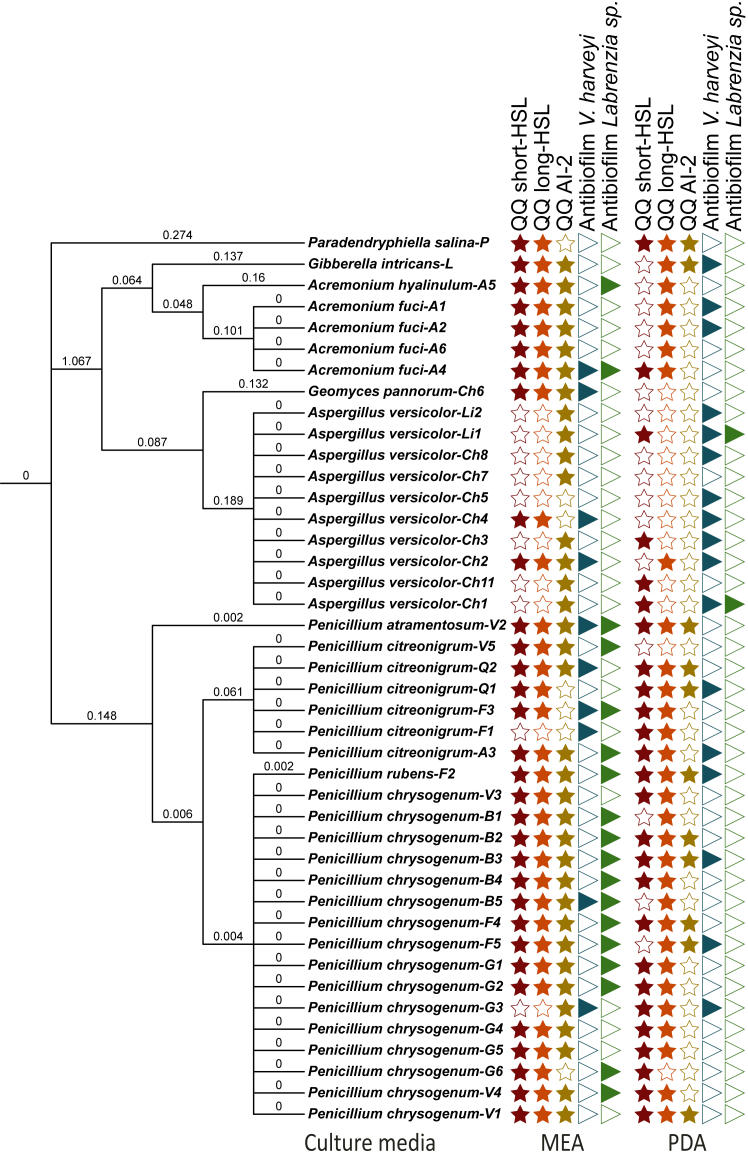
Bioactivities of fungal strains in MEA and PDB media Phylogenetic tree of fungal strains and biotest results for each strain in MEA and PDA culture media. The numbers on the branches correspond to the genetic distance between strains.

The extracts used in the bioassays demonstrated no impact on the growth of the biosensors. However, their efficacy in modulating QS-mediated signaling was assessed. The results revealed that, among the 42 fungal strains (Figure 3; Tables S5 and S6), 61 and 76% of fungal extracts were able to inhibit short- and long-chain homoserine lactone (HSL)-based QS, respectively, in MEA and PDA culture media. AI-2-based QS was inhibited by 83% of fungal extracts in MEA culture medium and 26% in PDA. Among the extracts, *Acremonium* sp. strains stood out in the MEA culture medium by inhibiting all the signals involved in the QS tested. *Penicillium* sp. were also able to interact with QS compounds, with 80 and 92% of extracts inhibiting short- and long-chain HSL-based QS, respectively, and 84% inhibiting AI-2-based QS in MEA medium. In PDA medium, 84–92% of *Penicillium* sp. extracts were able to inhibit short- and long chain-HSL and 38% of AI-2-based QS. For *Aspergillus* sp. 20–70% inhibited long and short chain-HSL based QS and 80% inhibited AI-2-based QS in MEA, whereas in PDA, 10–40% inhibited long and short chain-HSL-based QS and 0% inhibited AI-2-based QS. *G. intricans* sp. strain inhibited all the compounds involved in QS tested (AHLs and AI-2) in MEA medium, while *P. salina* was able to inhibit them in PDA.

### Chemical mediators involved in QS produced by cultivable bacterial epimicrobiota

**Figure 4:**
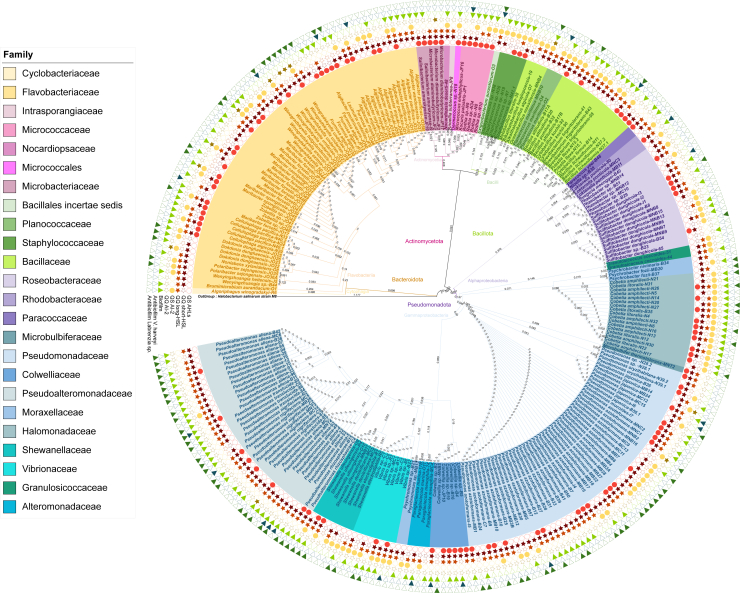
Bioactivities of bacterial strains in MB media Phylogenetic tree of bacterial strains and biotest results for each strain in MB culture medium. The numbers on the branches correspond to the genetic distance between strains.

The 272 bacterial strains isolated were screened for their ability to produce and inhibit QS mechanisms (short-HSL, long-HSL, and AI-2-based QS) (Figure 4; Table S7) in marine broth (MB). The results showed that 40% of the strains were able to produce AHL. Among the Micrococcaceae and Microbacteriaceae, 100% of the strains isolated were able to produce AHLs, as were 85% of the Colwelliaceae. Surprisingly, under our cultural conditions, Moraxellaceae, Alteromonadaceae, Planococcaceae, and Granulosicoccaceae were not able to produce AHLs. However, Granulosicoccaceae produced AI-2, as did Staphylococcaceae and Halomonadaceae. Overall, 44% of the strains in the collection produced AI-2 under our culture conditions.

Regarding QS inhibitors, 100% of strains of Granulosicoccaceae, Paracoccaceae, and Planococcaceae were able to inhibit short and long-HSL-based QS. Of the 272 strains, 83.5% produced short-HSL-based QS inhibitors, compared with 65% for long-HSL-based QS. Among short-HSL based-QS-inhibiting strains, Moraxellaceae (100%), Microbacteriaceae (100%), and 98% of Pseudomonadaceae were particularly productive of this type of inhibitor, while for long-HSL inhibition Vibrionaceae were the most active (87%). Most strains in the Pseudoaltermonadaceae family produced both short and long-HSL-based QS inhibitors (98% and 87%, respectively). Overall, for each bacterial genus, at least one strain is capable of inhibiting AHLs. However, only 3% of the strains isolated were able to inhibit AI-2-based QS, notably 20% of Microbacteriaceae and 12% of Flavobacteriaceae.

### Ability to form and inhibit bacterial biofilm formation

Extracts of 42 fungal strains grown in 2 culture media (MEA 75% NSW and PDA 75% NSW) were also tested for their ability to inhibit bacterial biofilms (Figure 3; Tables S5 and S6). *Vibrio harveyi* biofilm formation was inhibited in MEA by 20% of extracts produced by *Acremonium* sp*.* and *Aspergillus* sp*.* strains, and by 25% of those of *Penicillium* sp. Such an antibiofilm effect was also shown for the extract of *Geomyces pannorum* cultivated in MEA and for *Gibberella intricans* cultured in PDA. In this culture medium, 80% of *Aspergillus* sp*.*, 40% of *Acremonium* sp., and 25% of *Penicillium* sp. strains were able to inhibit biofilm formation. The biofilm of the *Labrenzia* sp. BBCC2184 strain was inhibited in MEA by 67% of the Penicilliums metabolome and 40% of the Acremoniums metabolome, but not by *Aspergillus* sp. In PDA, only 20% of *Aspergillus* sp. was able to inhibit biofilm formation. The biofilm-forming capacities of 272 bacterial strains were also tested and 59% were able to form biofilms under our culture conditions, notably 100% of Staphylococcaceae and Halomonadaceae, and 90% of Pseudoalteromonadaceae. Bacteria from the Microbacteriaceae and Colwelliaceae families were not able to form biofilms under our culture conditions (Figure 4; Table S7).

The biofilm growth inhibiting capacities of bacterial and fungal strains were also tested. Two biofilm inhibition tests were carried out, one on the biofilm-forming capacity of *Vibrio harveyi* and the second on *Labrenzia* sp. BBCC2184. Eight percent of bacterial supernatants were shown to inhibit *V. harveyi* biofilm without affecting its viability, notably 33% of Rhodobacteraceae and Planococcaceae strains, but also 28% of strains from the Colwelliaceae family. Regarding *Labrenzia* sp. BBCC2184 inhibition, 38% of bacterial strains inhibited its biofilm formation, 72% of Pseudoalteromonas, and 62% of Shewanellaceae inhibited its biofilm formation. Among the Flavobacteriaceae family, the genus *Dokdonia* was shown capable of inhibiting this type of biofilm.

## Discussion

### Cultivable diversity of the epimicrobiota of *Saccharina latissima*

The macroalgal surface epimicrobiome forms a complex biofilm with intricate microbial interactions.^12^^,^^15^ Few studies have been dedicated to the investigation of the epiphytic fungi associated with brown macroalgae, and the cultivable fungal diversity of the *S. latissima* epimicrobiome remains poorly explored. Our findings primarily noted the presence of epiphytic fungi belonging to the Dothideomycetes class (95%), with a predominant occurrence of *Penicillium* sp. (57%), *Aspergillus* sp. (24%), and a few *Acremonium* sp. (12%). Interestingly, our results reveal a significant disparity between the fungal epimicrobiota and the endomicrobiota, composed of Sordaryomycetes,^30^ indicating a potential specificity of the fungal community based on colonized algal tissues. Other studies have focused on the epiphytic fungi of brown macroalgae, with the authors isolating *Penicillium* sp*.*, *Acremonium* sp*.*, *Aspergillus* sp., as well as *Geomyces* sp. and *Paradendryphiella salina* from *Fucus* sp.^32^^,^^38^ *Penicillium* sp. is a commonly occurring genus within the algal epimicrobiota, and its presence is therefore not surprising.^100^

Regarding the cultivable bacterial diversity within the *S. latissima* epimicrobiota, our study revealed the presence of Pseudomonadota (Gammaproteobacteria and Alphaproteobacteria), Bacteroidota, Bacillota, and Actinomycetota. Previous studies on *S. latissima* have identified similar strains, including *Pseudoalteromonas* sp., *Sulfitobacter* sp*.*, *Granulosicoccus* sp*.*, *Maribacter* sp., and *Psychrobacter* sp. in different proportions.^27^^,^^101^^,^^102^^,^^103^ Notably, Lu et al. (2023) highlighted the significant presence of Flavobacteriaceae and Rhodobacteraceae within the core microbiota of brown macroalgae, constituting 23% and 8% of the cultivable bacterial community in our study. Furthermore, Dong and collaborators showed the predominance of *Pseudomonas* sp. and *Psychromonas* sp. within *S. latissima* from the Arctic, whereas, in our study, *Pseudomonas* sp. and *Pseudoalteromonas* sp. prevailed, with *Psychromonas* sp. being infrequently isolated.^101^ However, culture-based and metabarcoding approaches could lead to different but complementary biodiversity patterns. Variation in the composition and proportions of bacterial epimicrobiota among different studies can be attributed to diverse sampling locations, environmental factors (Arctic, Baltic Sea, China), algae habitat (natural or cultivable), and algal maturity.^104^ These variations may also reflect the capacity of macroalgae to modulate their surface epibionts based on biotic and abiotic parameters, similar to the “gardening” phenomenon observed in plants, where they chemically recruit microorganisms that contribute to the enhancement of seaweed fitness.^105^

### Diversity of fungal chemical mediators

Macroalgal extracts have been extensively studied for their production of various biologically active metabolites.^106^^,^^107^^,^^108^ However, the investigation into metabolites produced by macroalgal-associated fungi remains limited. As highlighted in a review by Papagianni et al.,^109^ each fungus exhibits unique metabolite production under distinct morphological and physiological conditions. Previous studies highlighted the production of secondary metabolites with antioxidant, antimicrobial and cytotoxic activities^33^^,^^34^^,^^35^ as well as anticancer activity.^32^ LC-MS/MS analysis of the epiphytic fungal metabolomes yielded 573 metabolites. We focused on annotating metabolites potentially involved in microbial interactions, revealing over 99 metabolites from the fungal microbiota of *S. latissima* engaged in a molecular dialogue. These include antibacterial (18), antifungal (9), and antimicrobial compounds (26). Interestingly, some of the identified putative compounds are related to one species, whereas others seem to be common in different fungal strains, suggesting both specific and shared biological roles.

Among the different chemical mediators, our interest extended to QS molecules, pivotal in microorganism communication, biofilm formation, and maintenance.^40^^,^^41^^,^^42^ Our group has also previously demonstrated the key role of QS in the interspecies interactions between fungal and bacterial endophytes associated with brown algae.^61^ Herein, we demonstrated that fungal strains from the epimicrobiota produce various QS signals able to be activators such as the precursor 2-heptylquinolin-4(1H)-one (HHQ),^92^ or inhibitors such as cyclopeptides (5).^110^^,^^111^^,^^112^^,^^113^ Moreover, in the coumarin family, 8 compounds were detected (Table S4), which features prominently in our investigation and are frequently associated with anti-QS and antibiofilm activities.^114^^,^^115^^,^^116^ Similarly, furanones and butenolides have been linked to anti-QS activities.^59^^,^^117^^,^^118^ Although less studied, beta-carbolines have also demonstrated potential in this regard.^87^ Consequently, many of the other annotated compounds in our study may possess QS or anti-QS activity, but they have never been tested for such activity.^119^ These results highlight the complex interactions that fungal strains can have with other microorganisms within the algal epimicrobiota through specific secondary metabolites.

### Role of the epimicrobiota in regulating bacterial biofilms

Fungal strains isolated from algal epimicrobiota have a strong potential to inhibit QS mechanisms, including AHLs and AI-2 based QS. Remarkably, 95% of strains cultivated in MEA culture media possess anti-QS activity, a phenomenon previously demonstrated in the marine environment^120^^,^^121^^,^^122^^,^^123^^,^^124^^,^^125^^,^^126^ and in endophytic fungi associated with brown algae.^30^^,^^61^ However, to our knowledge, the presence of such activities in epiphytic fungal strains of macroalgae has not been documented. Notably, our research findings also highlight the presence of bacterial biofilm-inhibiting strains in the MEA culture media, accounting for approximately 57% of the total strains. This indicates that fungi possess significant potential in regulating bacterial biofouling. To highlight the role of fungi in regulating the algal epimicrobiome, it would be particularly relevant to test the ability of fungal strains to inhibit biofilm formation by bacteria belonging to the core microbiota identified by Lu et al.^102^ While the production of antifouling compounds by marine fungi has been the subject of several studies, with an overview provided by Dobretsov et al.,^127^ only a limited number of studies have focused on the ecological role of fungi in the epiphytic biofilm of macroalgae. Our findings suggest that epiphytic fungi may play a regulatory role in the epimicrobiota of *S. latissima*, particularly in shaping bacterial communities acting on QS mechanisms. In particular, the production of secondary metabolites or anti-QS enzymes could regulate bacterial QS and biofilm formation. However, the diversity of quorum quenching (QQ) enzymes produced by fungal strains has not been studied here.

We investigated bacterial strains for their communication abilities through QS mechanisms (AHLs and AI-2 based QS), and biofilm formation capacity. Results revealed that 40% of bacterial strains were able to produce AHLs (short-, medium-, and long-chain HSL), 44% of the strains communicated via AI-2 and 59% demonstrated biofilm-forming capabilities under our specific culture conditions. This substantial communication capacity suggests a crucial role of QS in maintaining the surface biofilm of brown macroalgae. Similarly, previous studies indicated the involvement of QS in the protection of algae from biofouling through the action of QS inhibitors produced by epiphytic bacteria.^25^^,^^26^^,^^27^^,^^128^ Specifically, 87.5% of the bacterial strains inhibited short-chain HSL, and 65% inhibited long-chain HSL base QS. However, only 3% of the strains were capable of inhibiting AI-2 communication, and 8% exhibited biofilm formation inhibition. Moreover, the spectrum of QS compounds, which includes both activating and inhibiting agents sometimes produced by the same bacterial strains, shows remarkable diversity, with no exclusive association with a single bacterial genus. Therefore, the bacterial strains present in the *S. latissima* epimicrobiota exhibit the ability to influence the formation of algal biofilms. However, additional research is necessary to elucidate the precise mechanisms by which bacteria regulate the communities of epiphytic fungi. While Tourneroche et al.^61^ demonstrated the involvement of AI-2 in cross-kingdom signaling between bacteria and endophytic fungi in *Saccharina latissima*, it is plausible that other mechanisms may also contribute to these interactions.

### Brown algae epimicrobiota, a source of new bioactive and antifouling compounds

The microbiota of macroalgae is a source of new biologically active compounds such as antioxidant, antibacterial, and anticancer agents.^5^^,^^6^^,^^129^ Numerous studies have highlighted a wide range anti-QS compounds isolated from macroalgae, which could be used as innovative and low ecotoxic antifouling agents.^25^^,^^130^^,^^131^^,^^132^^,^^133^^,^^134^ One of the most well-known example are the halogenated furanones extracted from the red macroalga *D. pulchra*,^59^^,^^135^ although some others have since been identified such as 2-dodecanoyloxyethanesulfonate from *Asparagopsis taxiformis*.^25^ However, most of the bioactive anti-QS compounds previously identified, such as halogenated furanone, were also noted for their potential toxicity, reinforcing the need to pursue this exploration. Limited attention has been given to studying the anti-fouling properties of fungal strains of the brown algal epimicrobiota. Collectively, our findings establish that fungal strains originating from the epimicrobiota of brown macroalgae might represent a valuable source for anti-QS and antibiofilm compounds of interest. From our study, the epimicrobiota of brown algae, especially *Saccharina latissima*, represents a source of potential new compounds.

### Limitations of the study

Chemical databases to annotate LC-MS metabolomic profiles remain poorly documented especially for marine bacterial and fungal metabolites, limiting the identification of bioactive compounds in our study but this opens some very interesting perspectives for the discovery of novel antibiofilm compounds. The production of bioactive compounds by isolated strains might also be dependent on the selected media. We used two different media to overcome this limitation; however, we can expect the production of a wider diversity of pro-QS and anti-QS metabolites by each individual strain if multiplying culture conditions.

## STAR★Methods

### Key resources table

**Table:** 

REAGENT or RESOURCE	SOURCE	IDENTIFIER
**Bacterial and virus strains**		

Bacterial isolates	This study	Database: OR491302-OR491573 BIOPROJECT: PRJNA1010225
Fungi isolates	This study	Database: OR461711-OR461752 BIOPROJECT: PRJNA1010225
*Vibrio fischeri* BBCC707 (MOLA 707)	Blanchet et al.^136^ Banyuls Bacterial Culture Collection	N/A
*Shewanella pacifica* BBCC292 (MOLA292)	Blanchet et al.^136^ Banyuls Bacterial Culture Collection (Bio2Mar – EMBRC)	N/A
*Hyphomonas atlantica* BBCC116 (MOLA116)	Blanchet et al.^136^ Banyuls Bacterial Culture Collection (Bio2Mar – EMBRC)	N/A
*Microsphaeropsis olivacea* AN329T	Tourneroche et al.^61^	N/A
*Vibrio harveyi* F5-F11	This study – from Banyuls Bacterial Culture Collection (Bio2Mar – EMBRC)	N/A
*Labrenzia sp.* BBCC2184	Blanchet et al.^136^ Banyuls Bacterial Culture Collection (Bio2Mar – EMBRC)	N/A

**Chemicals, peptides, and recombinant proteins**		

Marine Broth 2216 Agar	BD Difco	Cat# 11718193
Marine Broth 2216	BD Difco	Cat# 11753513
Reasoner’s 2A agar (R2A)	BD Difco	Cat# 11798273
Potato dextrose agar	BD Difco	Cat# 10105824
Potato dextrose broth	BD Difco	Cat# 11738982
Malt extract	BD Difco	Cat# 11718123
Agar-agar	Sigma-Aldrich	CAS# 9002-18-0
Glucose	Sigma-Aldrich	CAS# 50-99-7
Kanamycin sulfate from Streptomyces	Sigma-Aldrich	CAS# 25389-94-0
Benzylpenicillin	Sigma-Aldrich	CAS# 69-57-8
Gentamycin	Sigma-Aldrich	CAS# 1405-41-0
Tetracycline	Sigma-Aldrich	CAS# 64-75-5
Chloramphenicol	Sigma-Aldrich	CAS# 56-75-7
C6-HSL	Cayman Chemical, Ann Arbor, MI, USA	CAS# 147852-83-3
3-oxo-C10-HSL	Cayman Chemical, Ann Arbor, MI, USA	CAS# 192883-12-8
4,5-dihydroxy-2,3-pentanedione (DPD)	Rita Ventura’s research Group at ITQB, Oeiras, Portugal) Ascenso et al.^137^	N/A
Crystal violet	Sigma-Aldrich	CAS# 548-62-9

**Critical commercial assays**		

Quick-DNA™ Fungal/Bacterial Miniprep Kit	Zymo Research	Cat# D6007
Agencourt AMPure XP kit	Beckman Coulter	Cat# A63880
BigDye Terminator technology	Thermo Fisher Scientific	Cat# 4337450
Agencourt CleanSeq kit	Beckman Coulter	Cat# A29151

**Deposited data**		

16S rRNA sequences for bacterial isolates	NCBI	Database: OR491302-OR491573 BIOPROJECT: PRJNA1010225
ITS fragment sequences for fungi isolates	NCBI	Database: OR461711-OR461752 BIOPROJECT: PRJNA1010225

**Experimental models: Organisms/strains**		

*Pseudomonas putida* F117 (pRK-C12; Kmr; ppuI::npt)	Andersen et al.^138^	N/A
*Escherichia coli* MT102 (pJBA132)	Riedel et al.^139^	N/A
*Chromobacterium violaceum* CV026	McClean et al.^140^	N/A
*Vibrio harveyi* MM32 (luxN::Cm, luxS::Tn5Kan)	Bassler et al.^141^	N/A

**Oligonucleotides**		

Primer ITS1F: 3'-AGGAGAAGTCGTAACAAGGT-5'	Romani et al.^142^	N/A
Primer ITS4R: 3'-TCCTCCGCTTATTGATATGC-5'	Romani et al.^142^	N/A

**Software and algorithms**		

Staden-GAP4	Staden package Staden et al.^143^	https://staden.sourceforge.net/
BLAST ITS sequences	Mycobank	https://www.mycobank.org/
BLAST ITS sequences	Unite	https://unite.ut.ee/
BLAST 16S rRNA sequences	EZBioCloud	http://ezbiocloud.net/
MEGA 11	MEGA Software	https://www.megasoftware.net/
DataAnalysis version 4.4	Bruker Daltonik GmbH	N/A
MzMine 3.2.8	MzMine 3 Myers et al. Pluskal et al. Schmid et al.^144^^,^^145^^,^^146^	https://mzmine.github.io/
Feature-Based Molecular Networking (FBMN)	Global Natural Products Social Molecular Networking (GNPS) Nothias et al.^147^	https://gnps.ucsd.edu/ProteoSAFe/static/gnps-splash.jsp
GNPS annotation tools	Global Natural Products Social Molecular Networking (GNPS)	https://gnps.ucsd.edu/ProteoSAFe/static/gnps-splash.jsp
Sirius 5.6.3	Lehrstuhl Bioinformatik Jena	https://bio.informatik.uni-jena.de/software/sirius/
Natural products database	NpAtlas	https://www.npatlas.org/
Natural products database	MarinLit	https://marinlit.rsc.org/
Natural products database	LOTUS	https://lotus.naturalproducts.net/
Cytoscape 3.10.0 software	Cytoscape Software Shannon et al.^148^	https://cytoscape.org
GraphPad Prism v8	Dotmatics	https://www.graphpad.com/features
Interactive Tree Of Life (iTOL – online platform)	Letunic and Bork^149^	https://itol.embl.de/

**Other**		

Sanger AB3130xl 16-capillary sequencer	Applied Biosystems	N/A
Bacterial extraction and sequencing, with primer 1040R-1040F and P8-PC535	Genoscreen company	https://www.genoscreen.fr/fr/
C18 Acclaim™ RSLC PolarAdvantage II column (2.1 × 100 mm, 2.2 μm pore size	Thermo Fisher Scientific	Ref 068990
Dionex Ultimate 3000 HPLC system	Thermo Fisher Scientific	Ref IQLAAAGABHFAPBMBFB
Maxis IITM QTOF mass spectrometer	Bruker	N/A
Victor 3 spectrofluorometer	Perkin Elmer	N/A

### Resource availability

#### Lead contact

Further information and requests for resources and reagents should be addressed to the main contacts, Raphaël Lami (raphael.lami@obs-banyuls.fr).

#### Materials availability

This study did not generate new unique reagents.

#### Data and code availability

All DNA sequences data were deposited to NCBI. Accession numbers are listed in the key resources table. Standardized biotest data are shown in Tables S5–S7. This paper does not report original code. Any additional information required to reanalyze the data reported in this paper is available from the lead contact upon request.

### Method details

#### Sampling and isolation of epiphytic microbial strains

Fungi and bacteria were previously isolated from the brown alga *S. latissima* located in Perharidy (48°43'47.0 "N 4°00'17.1 "W), Roscoff, France, in January and June 2021. During low tide (coefficients 93 and 101), six young and healthy (without visible damages) *Saccharina latissima* individuals (< 1 m) were randomly collected and placed in sterile plastic bags. The six individuals were promptly transported to the laboratory in a cooler at approximately 4°C and stored in a cold storage room until sampling (maximum 2 hours).^150^

To extract the microbial community, each alga was rinsed with sterile seawater, and the microbial samples were obtained by scraping with a scalpel and then swabbing.^151^ The swabs were then placed in 2 mL Eppendorf tubes containing sterile seawater and vortexed for 2 min. Serial dilutions of the samples were made in Eppendorf tubes up to 10^-4^ dilution.^142^ Subsequently, 20 μL of each sample (from 10^0^ to 10^-4^ dilutions) were spread with a rake on Petri dishes. For the first sampling (January 2021), the isolation culture media used were MA (marine agar, BD Difco) and R2A (Reasoner’s 2A agar, BD Difco) with 100 % natural seawater (NSW) for bacterial strains. For fungal strains, PDA (potato dextrose agar, BD Difco) with 75 % NSW and MEA (malt extract agar) with 75 % NSW were used.^30^ The composition of MEA is as follows: 20 g of malt extract (BD Difco), 20 g of glucose (Sigma Aldrich), 1 g of Bacto peptone (BD Difco), 20 g of agar-agar (Sigma Aldrich) for 1 L. During the second sampling (June 2021), the samples were only spread on MEA 75 % and PDA 75 %, both containing 1 g.L^-1^ kanamycin (kanamycin sulfate from *Streptomyces*, Sigma Aldrich) and 1 g.L^-1^ penicillin G (benzylpenicillin, Sigma Aldrich) and then incubated at 18°C in the dark.^152^^,^^153^

The cultures were subsequently maintained at 18°C in the dark until new colonies stopped appearing on the Petri dishes. Each colony was isolated and purified based to their distinct morphotypes on their respective media.^136^ The microbial strains were stored at -80°C in cryotubes. For bacteria, cryopreservation medium consisting of liquid cultures with 35 % glycerol was used, while for fungi, agar pieces were plated in cryotubes with 715 μL of PDA 75 % NSW and 215 μL of 70 % glycerol.

#### Taxonomic identification of bacterial and fungal epiphytic strains

The 42 fungal isolates were identified using routine protocols based on ITS sequencing^30^^,^^136^^,^^154^ without any modification. Agar pieces from each fungal strain were collected and placed into ZR BashingBead™ Lysis tubes from the Quick-DNA™ Fungal/Bacterial Miniprep Kit (Zymo Research). PCR amplification of the ITS fragment was carried out using the following primers: ITS1F (3'-AGGAGAAGTCGTAACAAGGT-5'; 10 μM) and ITS4R (3'-TCCTCCGCTTATTGATATGC-5'; 10 μM). The PCR products were purified using the Agencourt AMPure XP kit according to the manufacturer's instructions. The sequencing reaction was performed using BigDye Terminator technology (Nimagen), and the resulting products were cleaned using the Agencourt CleanSeq kit following the manufacturer's instructions. Sanger sequencing was performed with the Sanger AB3130xl 16-capillary sequencer (Applied Biosystems) on the Bio2Mar platform (Observatoire Oceanologique, Banyuls-sur-Mer, France). ITS gene sequences were trimmed, double checked manually, and dereplicated using the package Staden-GAP4.^143^ Each FASTA file was uploaded to Mycobank (https://www.mycobank.org/ ) or Unite (https://unite.ut.ee/ ) and compared with the cultured fungal strain databases using BLAST.

For the 272 bacterial isolates, DNA was extracted and sequenced by the company Genoscreen. Their two pairs of primers were used: 1040R-1040F and P8-PC535. The results were received as .ab1 and .seq files containing the unassigned sequences. Subsequently, phylogenetic affiliations were manually annotated on EZBioCloud (http://ezbiocloud.net/ using BLAST.

All sequences were submitted to NCBI (ITS sequences, accession numbers Database: OR461711- OR461752, 16S rRNA sequences, accession numbers Database: OR491302-OR491573) in BIOPROJECT: PRJNA1010225. All sequences (fungi and bacteria) were aligned using Muscle implemented in MEGA 11.^155^^,^^156^ The alignments were manually reviewed for any mismatches, and a phylogenetic tree was constructed using a maximum composite likelihood tree with the Kimura 2-parameter Model (K2), G+I model, and nearest-neighbor-interchange and neighbor-joining methods.^156^^,^^157^ The reliability of each node in the tree was assessed by bootstrapping over 500 replicates. The phylogenetic tree was then exported in Newick format and processed on the iTOL online platform, where the biotest data were incorporated.

#### Culture of strains

Bacterial strains were inoculated with 50 μL (from cryotube) in glass tubes containing 5 mL of MB (Marine Broth, BD Difco) for 24 h at 18°C. For the AHL inhibition tests (short chain-homoserine lactones and long chain-homoserine lactones also called short chain-HSL and long chain-HSL), the bacterial strains were cultured with 1 μM (C6-HSL or 3-oxo-C10-HSL, Cayman Chemical, Ann Arbor, MI, USA) corresponding to the specific biotest being conducted. Fungal strains were inoculated with 3 pieces of agar (1 cm^2^) from Petri dish cultures. The fungal strains were grown at 18°C for 7 days in 50 mL of PDB 75 % NSW (Potato Dextrose Broth, BD Difco) and liquid MEA 75 % NSW without agar (see previous).

#### Fungal and bacterial supernatants

Bacterial supernatants were obtained after sampling 2 mL of culture from glass tubes and centrifuged at 10,000 x g for 10 min. The resulting supernatant was stored at -80°C until further use for the biotests. Fungal supernatants were obtained after 7 days of growth, and 25 mL of culture was sampled and centrifuged at 10,000 x g for 30 minutes. The supernatant was then filtered through 4 μm and 0.2 μm filters. The filtered fungal supernatants were stored at -80°C for subsequent analysis.

Fungal metabolomes were extracted from the supernatants by the addition of 25 mL of ethyl acetate to 25 mL of supernatant and were then shaken overnight at 25°C. Subsequently, the ethyl acetate phase was carefully collected using a Pasteur pipette and transferred to preweighed hemolysis tubes. The collected ethyl acetate phase was evaporated to dryness and the resulting dry mass of each sample was recorded. Sterile culture media (liquid MEA and PDB) was also extracted using the same method. The samples were then stored at -20°C until further analysis.

#### LC–MS-based metabolomic analysis of fungal strains

The dried extracts of fungal supernatant were solubilized in methanol at a concentration of 10 mg.mL^-1^ and injected in High Performance Liquide Chromatography coupled to tandem Mass Spectrometry (HPLC-MS/MS). The analysis was performed in one batch and in a random sequence, with five samples followed by three quality controls (QCs), according to established protocols.^30^^,^^61^ The separation was carried out using a C18 Acclaim™ RSLC PolarAdvantage II column (2.1 × 100 mm, 2.2 μm pore size; Thermo Fisher Scientific, United States) connected to a Dionex Ultimate 3000 HPLC system. The column was coupled to a Maxis IITM QTOF mass spectrometer (Bruker, United States) equipped with an electrospray ionization source. The flow rate was set at 300 μL.min^–1^. The MS parameters were 3.5 kV of electrospray voltage, 35 psi of nebulizing gas (N_2_) pressure, drying gas (N_2_) flow rate of 8 L.min^–1^, and 200°C of drying temperature. The mobile phases were water (0.1 % formic acid) and acetonitrile (0.1 % formic acid, solvent B) following a gradient of Solvent B at 5, 50, 90, and 5 % for 0, 9, 15, and 21 min, respectively.

LC-MS/MS data were analyzed using DataAnalysis (version 4.4 Bruker Daltonik GmbH) and converted to “.mzXML”. The LC-MS/MS data were preprocessed on MzMine 3.2.8 (Pluskal et al., 2010; Schmid et al., 2023) with a noise level of 1E3 for MS1 and 1E2 for MS2, and the “ADAP chromatogram” was built with a minimum group size of scan of 2, a group intensity threshold and minimum highest intensity at 3E3.^144^ The chromatograms were resolved with “ADAP Module Disclaimer” with S/N threshold at 10, minimum feature height at 1, coefficient/area threshold at 10, 0.02-1.00 for peak duration range and 0.10-0.50 for RT wavelet range. The data were then deisotoped and filtered to only keep peaks with MS2 scans and a “RANSAC” alignment was performed. After alignment, the compounds in the culture media (MEA and PDB) were subtracted from the matrix using the "blank subtraction" tool. This step eliminates the compounds present in the culture media from the dataset. The data were then exported to Feature-Based Molecular Networking of the platform Global Natural Products Social Molecular Networking (FBMN-GNPS) to build a molecular network with a cosine of 0.7 and 4 minimum matched fragment ions, precursor ion mass tolerance (PIMT) and fragment ion mass tolerance (FIMT) at 0.05 Da.^147^^,^^158^ Metabolites were annotated with various GNPS tools, including Library Search, Dereplicator, Dereplicator +,^159^ and Sirius 5.6.3^160^ using the tools Molecular formula identification, ZODIAC, CSI:FingerID, and CANOPUS.^161^^,^^162^^,^^163^^,^^164^^,^^165^^,^^166^ Furthermore, molecular formulas were deduced from HRMS *m/z* values and subsequently searched and annotated using the natural products databases NPAtlas, MarinLit, and LOTUS.^167^^,^^168^^,^^169^ The annotations were thus added to the network using Cytoscape 3.10.0 software.^148^

#### Quorum sensing bioassay: Detection of production of AI-1 and AI-2

To detect AI-1 and AI-2, a set of biosensors was employed, considering the diversity of quorum sensing compounds. *Pseudomonas putida* F117 (pRK-C12; Kmr; ppuI::npt) was used to detect long acyl chain AHLs (>8 carbons in the acyl side chain),^138^ while *Escherichia coli* MT102 (pJBA132) was utilized for medium-chain AHLs (≤8 carbons in the acyl side chain).^139^ For the detection of short-chain AHLs (ranging from C4-HSL to C8-HSL), *Chromobacterium violaceum* CV026 was employed in liquid media.^140^ *Vibrio harveyi* MM32 (luxN::Cm, luxS::Tn5Kan) was used to detect the presence of AI-2 in bacterial culture supernatants.^61^^,^^141^^,^^170^ The experimental protocols used in this study were routine protocols previously published by our laboratory.^61^^,^^136^^,^^142^^,^^171^

Briefly, the biosensors F117, MT102, and CV026 were precultured in Luria Bertani (LB) medium at 30°C with gentamycin (20 μg.mL^-1^, Sigma-Aldrich), 37°C with tetracycline (25 μg.mL^-1^, Sigma-Aldrich), and 25°C with kanamycin (20 μg.mL^-1^, Sigma-Aldrich), respectively, for 24 hours. In 96-well microplates, 50 μL of bacterial supernatant was added to 150 μL of triplicate biosensor culture (diluted 1/50 in growth media) and incubated for 24 hours at the corresponding preculture temperature. Two negative controls were used for all tests: (i) sterile marine broth media alone and (ii) biosensor supplemented with culture medium. The positive controls for biosensors F117, MT102, and CV026 were two commercial AHLs (C6-HSL and 3-oxo-C10-HSL at 10 μM) diluted in culture medium. After 24 hours, the cell growth (OD_630nm_) and GFP fluorescence (OD_535nm_) of F117 and MT102 were measured using a Victor 3 spectrofluorometer (Pelkin Elmer®). For the CV026 biosensor, violacein production was directly observed after 24 hours, then cell growth (OD_630nm_) and violet coloration (OD_540nm_) were measured.

*V. harveyi* MM32 reporter strain was grown at 30°C in marine broth medium supplemented with kanamycin (30 μg.mL^-1^, Sigma-Aldrich) and chloramphenicol (10 μg.mL-1, Sigma-Aldrich). In a 96-well plate, 180 μL of biosensor (diluted at 1/100 in growth media) was added to wells containing 20 μL of bacterial strain supernatants (in triplicate) for AI-2 production testing. All tests were performed with two negative controls: (i) sterile MB alone and (ii) biosensor supplemented with supernatant culture medium. The positive controls were a culture supernatant from strain BBCC292 (MOLA292) and BBCC707 (MOLA707), known to be a strong AI-2 producer.^136^ After 24 hours at 30°C, cell growth (OD_630nm_) and luminescence (OD_540nm_) of MM32 were measured using a Victor 3 spectrofluorometer (Pelkin Elmer®).

#### Quorum quenching bioassays: Detection of AI-1 and AI-2 inhibition

To measure AI-1 inhibition by culture supernatants or extracts, the biosensor cultures were supplemented with 3 μM AHLs, 3-oxo-C10-HSL was added for F117 and MT102 and C6-HSL for CV026 and MT102, which can detect the inhibition of both types of AHLs. In the microplate, 150 μL (for bacterial test) or 180 μL (for fungal test) of biosensors (diluted at 1/50 in growth media) supplemented with AHLs were added, followed by 50 μL of bacterial culture supernatants or 20 μL of 1 mg.mL^-1^ fungal extract (dissolved in 10% DMSO). The microplates were incubated, and after 24 h, the same measurements as described before were performed. However, the inhibition of the biosensor signal was calculated to determine the percentage of AI-1 inhibition. Two negative controls were used for the tests: (i) biosensors without AHLs with the addition of culture medium or culture medium extract dissolved in dimethylsulfoxyde (DMSO, 10 %) and (ii) biosensors with AHLs and the addition of sterile culture medium or culture medium extract diluted in 10% DMSO. After 24h hour, the same measurements were made as above.

For the detection of AI-2 inhibition, *Vibrio harveyi* MM32 biosensor was used in the presence of 1 μM of 4,5-dihydroxy-2,3-pentanedione (DPD, a precursor of AI-2 from Rita Ventura’s research Group at ITQB, Oeiras, Portugal).^137^ In a microplate, 20 μL of culture supernatant or fungal extract was added to 180 μL of biosensor (diluted at 1/100 in growth media) and DPD. The negative controls were (i) biosensor without DPD and (ii) biosensor with DPD with the addition of the culture medium of the tested supernatants or the extracted culture medium of the extracts. The positive control used was the active extract of the fungal strain *Microsphaeropsis olivacea* AN329T dissolved in DMSO 10%, as highlighted in.^61^ After 24h of incubation, the measurements as in the section Detection of AI-1 and AI-2 production, were performed.

#### Evaluation of bacterial biofilm-forming capacity

The biofilm-forming capacity of bacterial strains in the collection was assessed following previously described protocols.^136^^,^^172^ The strains were grown in microplates at 18°C for 5 days. After measuring the optic density OD_630nm_ with a Victor 3 spectrofluorometer (Perkin Elmer®), the microplates were rinsed with Phosphate Buffered Saline (PBS), dried, and then treated with crystal violet (0.2 % Sigma, diluted in 20 % ethanol) for 15 min in the dark at room temperature. Subsequently, the microplates were rinsed with water (three times) and the wells were decolorized with a decolorizing solution (33 % glacial acetic acid) to reveal the presence of biofilm. The OD_540nm_ was measured with a spectrofluorometer.

#### Biofilm inhibition

To evaluate the capacity of microbial strains to inhibit biofilm formation, two reference strains were used: *Vibrio harveyi* and *Labrenzia* sp. BBCC2184, which were obtained from our laboratory collections. These strains were grown in 48-well microplates (900 μL) at 25°C in MB, in the presence of 100 μL of bacterial supernatants for 48h before biofilms revelation (as described above). For inhibition test of biofilm formation by fungal metabolomes, 950 μL of bacterial strains was incubated with 50 μL of fungal extract at a concentration of 1 mg.mL^-1^ in DMSO (10 %) to limit the potential toxicity of fungal strains. The biofilm inhibition test was performed following the same procedure as that for biofilm formation (as described above).

#### Processing of results, statistical analyses, and graphic representations

A first processing of the data was performed, and an average of triplicates for each strain and extract was obtained at the end of each treatment. To assess biofilm formation for each strain relative to its growth, the ratio **dOD**_**540nm**_**/dOD**_**620nm**_ was calculated with violet staining; on the one hand, **dOD**_**540nm**_ **= OD**_**s 540nm**_ **- OD**_**m 540nm,**_ i.e., the difference between the staining of the strain of interest (**OD**_**s 540nm**_) and the culture medium control (**OD**_**m 540nm**_); and on the other hand, the growth **dOD**_**620nm**_ **= OD**_**s 620nm**_ **- OD**_**m 620nm**_ with the difference in opacity/growth between the strain of interest (**OD**_**s 620nm**_) and the culture medium (**OD**_**m 620nm**_).

For quorum sensing (AI-1 and AI-2), the % induction was calculated as follows for MT102 and F117 fluorescence of GFP, and the calculations were the same for MM32 bioluminescence. Thus, the percentage of fluorescence induction was calculated as follows**: % Fluorescence = (Signal**_**SN /E**_ **- Signal**_**Control**_ **)/Signal**_**Control**_ **∗ 100** with the fluorescence (GFP) signal normalized to OD_630nm_ after subtraction of the culture media, with **Signal**_**SN/E**_ **= dGFP/dOD**_**630nm**_ **= (GFP**_**SN**_ **- GFP**_**media**_**)/(OD**_**SN630nm**_**- OD**_**media630nm**_**)** where **GFP**_**SN**_ and **OD**_**SN630nm**_ correspond to the wells containing the biosensor with the tested supernatant/extract and **GFP**_**media**_ and **OD**_**media630nm**_ correspond to the controls culture medium alone without biosensor (LB or MB) and on the other hand: **Signal**_**Control**_ = **dGFP**_**controle**_ **/ dOD**_**controle630nm**_ **= (GFP**_**Controle**_ **– GFP**_**media**_**)/(OD**_**Controle630nm**_ **- OD**_**media630nm**_**)** where GFP_control_ and OD_control_ correspond to biosensor wells with sterile culture medium of supernatants or solvent of extracts.

To evaluate the inhibition of biofilm formation and quorum sensing, similar calculations were performed as mentioned above. The percentage of inhibition was calculated for purple coloring OD_540nm_ (biofilm test), bioluminescence (MM32) and GFP fluorescence (MT102 and F117). The % inhibition was calculated as follows: **% fluorescence = (Signal**_**Control**_ **- Signal**_**SN/E**_**)/Signal**_**Control**_ **∗ 100.** However, GFP_control_ and OD_control_ correspond to the biosensor with AHLs or DPD in the presence of supernatant culture medium or solvent. The biofilm inhibition test corresponds to the reporter strains (*Vibrio harveyi* or *Labrenzia sp.*) in the presence of supernatant culture medium or solvent.

The results of the biotests were processed separately, and the statistical tests (Mann‒Whitney tests) were performed using GraphPad Prism 8. The results were then transformed into a binary dataset based on significance and added to the phylogenetic trees via the iTOL online platform.
