# Supplementary material for: Importance of quorum sensing crosstalk in the brown alga Saccharina latissima epimicrobiome
Source: iScience. 2024 Feb 9;27(3):109176. doi: 10.1016/j.isci.2024.109176 (PMC10906538; doi:10.1016/j.isci.2024.109176)
Supplement: Document S1. Figure S1 and Tables S1–S7 [file mmc1.pdf]

iScience, Volume *27*

Supplemental information

Importance of quorum sensing crosstalk in the brown alga *Saccharina latissima* epimicrobiome

Emilie Adouane, Camille Mercier, Jeanne Mamelle, Emma Willocquet, Laurent Intertaglia, Bertille Burgunter-Delamare, Catherine Leblanc, Sylvie Rousvoal, Raphaël Lami, and Soizic Prado

SUPPLEMENTAL INFORMATION TITLES AND LEGENDS

Figure S1: Metabolomes of 42 isolated fungal strains in MEA and PBD media, related to Figure 2. Featured-Based Molecular-Networking (obtained on GNPS) with parameters Min Pairs Cos=0.7 and Minimum Matched Fragment Ions=4.

Table S1: Identification of fungal strains, related to Figure 1.

Table S2: Identification of bacterial strains, related to Figure 1.

Table S3: Metabolites within the epimicrobiota of *S. latissima*, related to Figure 2. *MS data after Mzmine 3.2.8 analysis (1442 features).*

Table S4: Putative annotation of fungal metabolomes, related to Figure 2.

Table S5: Binary results of fungal biotests in MEA culture medium, related to Figure 3.

Table S6: Binary results of fungal biotests in PDB culture medium, related to Figure 3.

Table S7: Binary results of bacterial biotests in MB culture medium, related to Figure 4.

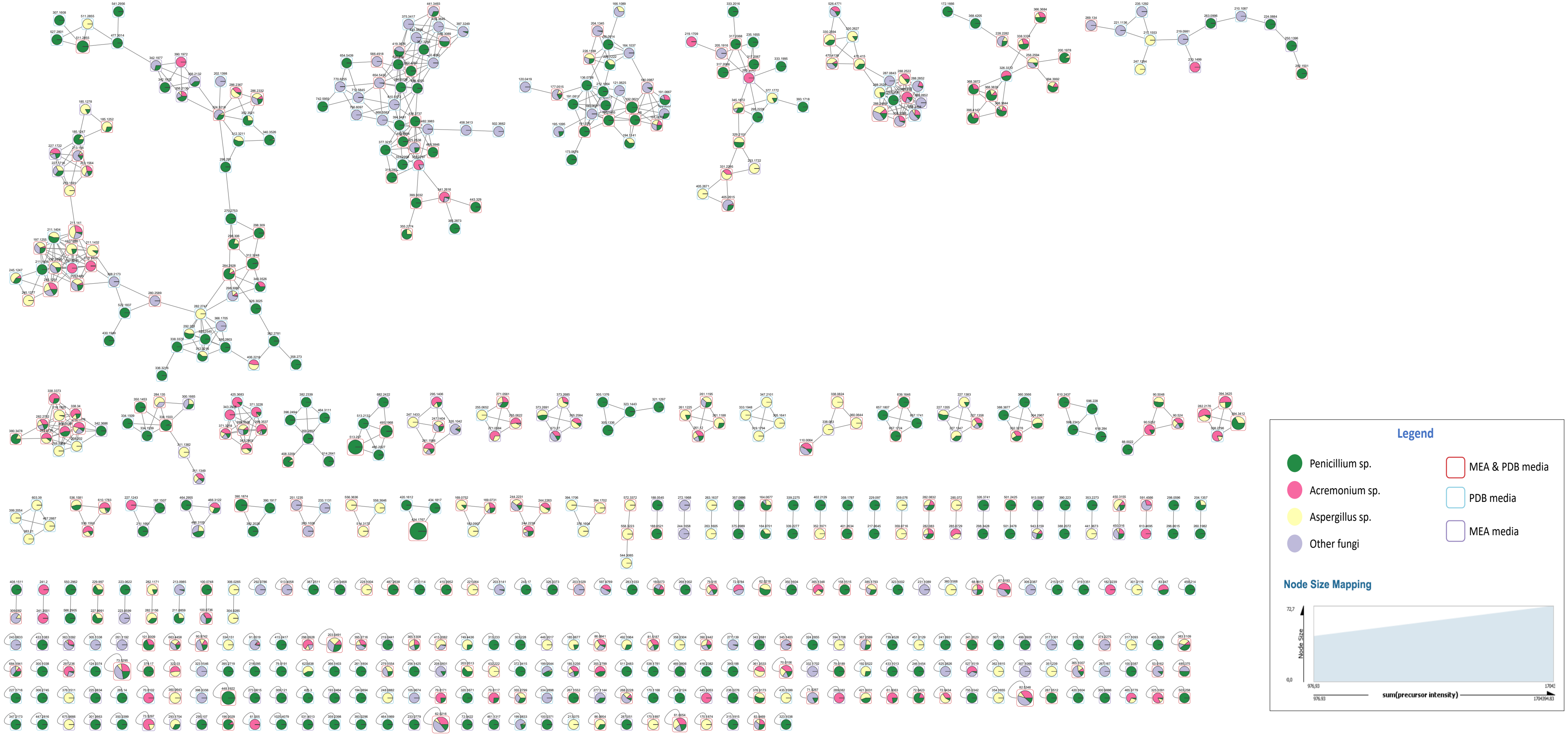
Legend
Penicillium sp.
Acremonium sp.
Aspergillus sp.
Other fungi
MEA & PDB media
PDB media
MEA media
Node Size Mapping
Node Size
sum(precursor intensity)

Name	Putative Identification	% Identity ITS4-R
Li1	Aspergillus versicolor	100
Li2	Aspergillus versicolor	100
Ch1	Aspergillus versicolor	100
Ch2	Aspergillus versicolor	100
Ch3	Aspergillus versicolor	100
Ch4	Aspergillus versicolor	100
Ch5	Aspergillus versicolor	100
Ch6	Geomyces pannorum var. pannorum	100
Ch7	Aspergillus versicolor	100
Ch8	Aspergillus versicolor	100
Ch11	Aspergillus versicolor	100
V1	Penicillium chrysogenum	100
V2	Penicillium atramentosum	100
V3	Penicillium chrysogenum	100
V4	Penicillium chrysogenum	100
V5	Penicillium citreonigrum	100
G1	Penicillium chrysogenum	100
G2	Penicillium chrysogenum	100
G3	Penicillium chrysogenum	100
G4	Penicillium chrysogenum	100
G5	Penicillium chrysogenum	100
G6	Penicillium chrysogenum	100
A1	Acremonium fuci	100
A2	Acremonium fuci	100
A3	Penicillium citreonigrum	100
A4	Acremonium fuci	100
A5	Acremonium hyalinulum (Acremonium sp.)	96,38
A6	Acremonium fuci	100
B1	Penicillium chrysogenum	100
B2	Penicillium chrysogenum	100
B3	Penicillium chrysogenum	100
B4	Penicillium chrysogenum	100
B5	Penicillium chrysogenum	100
F1	Penicillium citreonigrum	100
F2	Penicillium rubens	100
F3	Penicillium citreonigrum	100
F4	Penicillium chrysogenum	100
F5	Penicillium chrysogenum	100
L	Gibberella intricans	99.80
Q1	Penicillium citreonigrum	100
Q2	Penicillium citreonigrum	100
P	Paradendryphiella salina	100

Name	Putative identification	1040F		1040R	
		Top blast	Identity %	Top blast	Identity %
4	*Pseudoalteromonas aliena*	*Pseudoalteromonas aliena*	99,7	*Pseudoalteromonas aliena*	99,85
5	*Pseudoalteromonas aliena*	*Pseudoalteromonas aliena*	99,84	*Pseudoalteromonas aliena*	99,7
12	*Pseudoalteromonas aliena*	*Pseudoalteromonas aliena*	99,85	*Pseudoalteromonas aliena*	99,85
15	*Pseudoalteromonas aliena*	*Pseudoalteromonas aliena*	100	*Pseudoalteromonas aliena*	100
16	*Pseudoalteromonas aliena*	*Pseudoalteromonas aliena*	99,55	*Pseudoalteromonas aliena*	99,85
17b	*Paraglaciecola marina*	*Paraglaciecola marina*	98,51	*Paraglaciecola marina*	98,95
19	*Alkalihalobacillus hwajinpoensis*	*Alkalihalobacillus hwajinpoensis*	100	*Alkalihalobacillus hwajinpoensis*	98,68
29	*Pseudoalteromonas aliena*	*Pseudoalteromonas aliena*	99,85	*Pseudoalteromonas aliena*	99,85
32	*Pseudoalteromonas aliena*	*Pseudoalteromonas aliena*	99,85	*Pseudoalteromonas aliena*	99,85
33	*Pseudoalteromonas aliena*	*Pseudoalteromonas aliena*	100	*Pseudoalteromonas aliena*	100
38	*Metabacillus litoralis*	*Metabacillus litoralis*	99,4	*Metabacillus litoralis*	99,4
41	*Brevibacterium frigoritolerans*	*Brevibacterium frigoritolerans*	100	*Brevibacterium frigoritolerans*	100
44	*Granulosicoccus coccoides*	*Granulosicoccus coccoides*	98,66	*Granulosicoccus marinus*	98,8
48	*Nocardiopsis deserti*	*Nocardiopsis dassonvillei subsp. crassaminis*	95,36	*Nocardiopsis deserti*	98,4
49	*Pseudoalteromonas aliena*	*Pseudoalteromonas aliena*	99,85	*Pseudoalteromonas aliena*	99,85
51	*Granulosicoccus coccoides*	*Granulosicoccus coccoides*	98,65	*Granulosicoccus coccoides*	98,65
53	*Pseudoalteromonas aliena*	*Pseudoalteromonas aliena*	99,85	*Pseudoalteromonas aliena*	99,7
65	*Sulfitobacter donghicola*	*Sulfitobacter indolifex*	86,93	*Sulfitobacter donghicola*	99,07
74	*Paraglaciecola mesophila*	*Paraglaciecola mesophila*	100	*Paraglaciecola mesophila*	100
80	*Loktanella ponticola*	*Loktanella ponticola*	91	*Loktanella ponticola*	99,07
84	*Pseudoalteromonas translucida*	*Pseudoalteromonas translucida*	100	*Pseudoalteromonas translucida*	99,85
85	*Pseudoalteromonas translucida*	*Pseudoalteromonas translucida*	100	*Pseudoalteromonas translucida*	100
86	*Pseudoalteromonas translucida*	*Pseudoalteromonas translucida*	100	*Pseudoalteromonas translucida*	100
87	*Pseudoalteromonas nigrifaciens*	*Pseudoalteromonas nigrifaciens*	99,33	*Pseudoalteromonas nigrifaciens*	100
88	*Vibrio sp.*	*Vibrio splendidus*	100	*Vibrio splendidus*	100

89	*Pseudoalteromonas translucida*	*Pseudoalteromonas translucida*	100	*Pseudoalteromonas translucida*	100
90	*Cytobacillus kochii*	*Bacillus canaveralius*	89,61	*Cytobacillus kochii*	99,85
91	*Pseudoalteromonas aliena*	*Pseudoalteromonas aliena*	100	*Pseudoalteromonas aliena*	96,39
92	*Cytobacillus kochii*	*Cytobacillus kochii*	100	*Cytobacillus kochii*	96,21
93	*Pseudoalteromonas aliena*	*Pseudoalteromonas aliena*	99,85	*Pseudoalteromonas aliena*	99,85
94	*Pseudoalteromonas aliena*	*Pseudoalteromonas aliena*	99,95	*Pseudoalteromonas aliena*	99,95
95	*Pseudoalteromonas aliena*	*Pseudoalteromonas aliena*	99,85	*Pseudoalteromonas aliena*	99,85
96	*Pseudoalteromonas aliena*	*Pseudoalteromonas aliena*	99,85	*Pseudoalteromonas aliena*	99,85
97	*Pseudoalteromonas aliena*	*Pseudoalteromonas aliena*	99,85	*Pseudoalteromonas aliena*	99,85
98	*Brevibacterium frigoritolerans*	*Peribacillus simplex*	91,53	*Brevibacterium frigoritolerans*	99,85
O1	*Brumimicrobium aurantiacum*	*Brumimicrobium aurantiacum*	98,78	*Brumimicrobium aurantiacum*	98,79
O2	*Planococcus maritimus*	*Planococcus maritimus*	94,74	*Planococcus maritimus*	99,85
O3	*Exiguobacterium aurantiacum*	*Exiguobacterium profundum*	96,16	*Exiguobacterium aurantiacum*	99,7
O4	*Planococcus maritimus*	*Planococcus maritimus*	100	*Planococcus maritimus*	100
O5	*Zobellia sp.*	*Zobellia galactanivorans*	98,03	*Zobellia galactanivorans*	97,89
O7	*Alkalihalobacillus algicola*	*Alkalihalobacillus algicola*	100	*Alkalihalobacillus algicola*	99,85
O8	*Nonlabens xylanidelens*	*Nonlabens xylanidelens*	100	*Nonlabens xylanidelens*	98,96
O9	*Shewanella ulleungensis*	*Shewanella ulleungensis*	100	*Shewanella ulleungensis*	100
O10	*Shewanella ulleungensis*	*Shewanella ulleungensis*	100	*Shewanella ulleungensis*	99,85
O11	*Paraglaciecola marina*	*Paraglaciecola marina*	98,51	*Paraglaciecola marina*	98,96
JO1	*Algibacter pacificus*	*Algibacter wandonensis*	98,94	*Algibacter pacificus*	99,24
JO2	*Algibacter miyuki*	*Algibacter wandonensis*	95,51	*Algibacter miyuki*	99,7
JO8	*Maribacter forsetii*	*Maribacter forsetii*	100	*Maribacter forsetii*	100
JO9	*Maribacter caenipelagi*	*Maribacter caenipelagi*	99,85	*Maribacter caenipelagi*	99,7
JO10	*Algibacter pacificus*	*Algibacter wandonensis*	98,79	*Algibacter pacificus*	99,39
JO12	*Winogradskyella eximia*	*Winogradskyella eximia*	94,79	*Winogradskyella eximia*	99,7
JO13	*Algibacter pacificus*	*Algibacter wandonensis*	98,79	*Algibacter pacificus*	99,24
JO14a	*Algibacter miyuki*	*Algibacter miyuki*	96,06	*Algibacter miyuki*	99,85

JO14b	*Algibacter miyuki*	*Algibacter miyuki*	99,85	*Algibacter miyuki*	99,85
JO15	*Algibacter miyuki*	*Algibacter miyuki*	99,39	*Algibacter miyuki*	99,39
JO17a	*Maribacter forsetii*	*Maribacter forsetii*	99,69	*Maribacter forsetii*	98,38
JO17b	*Maribacter forsetii*	*Maribacter forsetii*	100	*Maribacter forsetii*	100
JO20	*Winogradskyella undariae*	*Winogradskyella undariae*	95,65	*Winogradskyella undariae*	99,85
JO21	*Winogradskyella undariae*	*Winogradskyella undariae*	97,13	*Winogradskyella undariae*	99,85
JO22	*Dokdonia donghaensis*	*Dokdonia donghaensis*	99,7	*Dokdonia donghaensis*	99,55
JO23	*Dokdonia donghaensis*	*Dokdonia donghaensis*	99,4	*Dokdonia donghaensis*	99,55
JO25	*Dokdonia donghaensis*	*Dokdonia donghaensis*	99,7	*Dokdonia donghaensis*	99,7
JO26	*Dokdonia donghaensis*	*Dokdonia donghaensis*	99,7	*Dokdonia donghaensis*	99,7
JO27	*Winogradskyella undariae*	*Winogradskyella undariae*	100	*Winogradskyella undariae*	99,85
JO29	*Winogradskyella arenosi*	*Winogradskyella arenosi*	98,94	*Winogradskyella arenosi*	98,94
JO30	*Formosa algae*	*Formosa algae*	99,09	*Formosa algae*	99,09
JO31	*Maribacter forsetii*	*Maribacter forsetii*	100	*Maribacter forsetii*	100
JO32	*Maribacter forsetii*	*Maribacter forsetii*	100	*Maribacter forsetii*	100
JO33	*Algibacter pacificus*	*Algibacter wandonensis*	99,1	*Algibacter pacificus*	99,24
JO35	*Polaribacter sejongensis*	*Polaribacter sejongensis*	99,7	*Polaribacter sejongensis*	99,7
JO36a	*Polaribacter sejongensis*	*Polaribacter sejongensis*	99,7	*Polaribacter sejongensis*	99,7
JO36b	*Algibacter pacificus*	*Algibacter pacificus*	99,39	*Algibacter pacificus*	99,39
JO37	*Algibacter wandonensis*	*Algibacter wandonensis*	99,1	*Algibacter pacificus*	98,94
JO39	*Maribacter spongiicola*	*Maribacter spongiicola*	99,41	*Maribacter spongiicola*	99,23
JO40	*Zobellia sp.*	*Zobellia galactanivorans*	98,48	*Zobellia galactanivorans*	98,2
JO41	*Zobellia sp.*	*Zobellia galactanivorans*	98,05	*Zobellia galactanivorans*	98,18
JO42	*Zobellia sp.*	*Zobellia galactanivorans*	99,1	*Zobellia laminariae*	98,94
JO43	*Winogradskyella undariae*	*Winogradskyella arenosi*	96,41	*Winogradskyella undariae*	99,85
JO44a	*Maribacter forsetii*	*Maribacter forsetii*	100	*Maribacter forsetii*	99,85
JO44b	*Maribacter forsetii*	*Maribacter forsetii*	100	*Maribacter forsetii*	100
JO45	*Maribacter spongiicola*	*Maribacter spongiicola*	99,55	*Maribacter spongiicola*	99,55

JO46	*Formosa sp.*	*Formosa algae*	97,43	*Formosa algae*	97,43
JO47	*Maribacter forsetii*	*Maribacter forsetii*	99,66	*Maribacter forsetii*	100
JO48	*Algibacter miyuki*	*Algibacter miyuki*	99,85	*Algibacter miyuki*	99,85
JO49	*Maribacter forsetii*	*Maribacter forsetii*	100	*Maribacter forsetii*	100
JO50	*Algibacter pectinivorans*	*Algibacter pectinivorans*	100	*Algibacter pectinivorans*	100
JO51	*Algibacter miyuki*	*Algibacter miyuki*	99,55	*Algibacter miyuki*	99,85
JO52	*Algibacter miyuki*	*Algibacter miyuki*	99,39	*Algibacter miyuki*	99,54
Jf2	*Microbacterium diaminobutyricum*	*Microbacterium diaminobutyricum*	99,23	*Microbacterium diaminobutyricum*	99,23
Jf3	*Microbacterium diaminobutyricum*	*Microbacterium diaminobutyricum*	99,24	*Microbacterium diaminobutyricum*	99,24
Jf9	*Lacinutrix undariae*	*Lacinutrix undariae*	99,55	*Lacinutrix undariae*	99,85
Jf16	*Micrococcus endophyticus*	*Micrococcus luteus*	99,84	*Micrococcus endophyticus*	98,95
Jf17	*Microbacterium diaminobutyricum*	*Microbacterium diaminobutyricum*	98,76	*Microbacterium diaminobutyricum*	99,09
Jf18	*Winogradskyella helgolandensis*	*Winogradskyella helgolandensis*	100	*Winogradskyella helgolandensis*	100
Jf19	*Salinibacterium amurskyense*	*Salinibacterium amurskyense*	100	*Salinibacterium amurskyense*	100
Jf20	*Maribacter forsetii*	*Maribacter forsetii*	100	*Maribacter forsetii*	100
Jp1	*Kocuria palustris*	*Kocuria palustris*	100	*Kocuria palustris*	100
Jp3	*Formosa algae*	*Formosa algae*	97,79	*Formosa algae*	99,85
Jp8	*Knoellia subterranea*	*Knoellia subterranea*	99,39	*Knoellia subterranea*	99,85
Jp9	*Winogradskyella undariae*	*Winogradskyella undariae*	96,2	*Winogradskyella undariae*	99,85
Jp10	*Colwellia sp.*	*Colwellia AX746195_s*	99,85	*Colwellia AX746195_s*	88,2
Jp11	*Maribacter forsetii*	*Maribacter forsetii*	100	*Maribacter forsetii*	99,85
Jp12	*Formosa algae*	*Formosa algae*	99,85	*Formosa algae*	99,85
J1	*Winogradskyella sp.*	*Winogradskyella ABHI_s*	94,16	*Winogradskyella ABHI_s*	99,7
J2	*Formosa algae*	*Formosa algae*	99,85	*Formosa algae*	99,85
J3	*Maribacter forsetii*	*Maribacter forsetii*	100	*Maribacter forsetii*	100
J4	*Algibacter miyuki*	*Algibacter miyuki*	97,75	*Algibacter miyuki*	98,33

J5	*Wenyingzhuangia heitensis*	*Wenyingzhuangia heitensis*	98,33	*Wenyingzhuangia heitensis*	98,32
R1	*Shewanella electrodiphila*	*Shewanella electrodiphila*	100	*Shewanella electrodiphila*	100
R2	*Pseudoalteromonas nigrifaciens*	*Pseudoalteromonas nigrifaciens*	99,85	*Pseudoalteromonas nigrifaciens*	100
R3	*Vibrio sp.*	*Vibrio splendidus*	100	*Vibrio splendidus*	100
R4	*Shewanella ulleungensis*	*Shewanella ulleungensis*	100	*Shewanella ulleungensis*	99,85
R5	*Shewanella electrodiphila*	*Shewanella electrodiphila*	100	*Shewanella electrodiphila*	100
R6	*Algoriphagus winogradskyi*	*Algoriphagus winogradskyi*	99,4	*Algoriphagus winogradskyi*	99,55
Mnb2	*Pseudomonas azotoformans*	*Pseudomonas azotoformans*	99,7	*Pseudomonas azotoformans*	99,4
Mnb4	*Oceanobacillus massiliensis*	*Oceanobacillus massiliensis*	92,31	*Oceanobacillus massiliensis*	99,85
Mnb5	*Colwellia sp.*	*Colwellia AX746195_s*	69,47	*Colwellia AX746195_s*	100
Mnb6	*Sulfitobacter donghicola*	*Tateyamaria pelophila*	69,35	*Sulfitobacter donghicola*	99,07
Mnb7	*Sulfitobacter donghicola*	*Sulfitobacter donghicola*	89,6	*Sulfitobacter donghicola*	99,39
Mnb8	*Sulfitobacter donghicola*	*Roseobacter cerasinus*	68,8	*Sulfitobacter donghicola*	99,54
Mnb9	*Sulfitobacter donghicola*	*Tateyamaria pelophila*	69,12	*Sulfitobacter donghicola*	99,07
Mnb10	*Planococcus maritimus*	*Planococcus maritimus*	100	*Planococcus maritimus*	99,85
Mnb11	*Shewanella ulleungensis*	*Shewanella ulleungensis*	100	*Shewanella ulleungensis*	100
Mnb12	*Phaeobacter sp.*	*Phaeobacter sp.*	99	*Phaeobacter sp.*	99,75
Mnb13	*Sulfitobacter donghicola*	*Sulfitobacter porphyrae*	71,1	*Sulfitobacter donghicola*	99,53
Mnb14	*Phaeobacter sp.*	*Phaeobacter sp.*	99	*Phaeobacter sp.*	99,69
Mnb15	*Sulfitobacter donghicola*	*Roseobacter cerasinus*	70,38	*Sulfitobacter donghicola*	99,54
Mnb16	*Pseudomonas azotoformans*	*Pseudomonas azotoformans*	99,7	*Pseudomonas azotoformans*	99,7
Mc1	*Vibrio sp.*	*Vibrio splendidus*	100	*Vibrio splendidus*	100
Mc2	*Shewanella denitrificans*	*Shewanella denitrificans*	100	*Shewanella denitrificans*	100
Mc4	*Paraglaciecola mesophila*	*Paraglaciecola mesophila*	100	*Paraglaciecola mesophila*	100
Mc5	*Pseudoalteromonas nigrifaciens*	*Pseudoalteromonas nigrifaciens*	100	*Pseudoalteromonas nigrifaciens*	100
Mc6	*Pseudoalteromonas aliena*	*Pseudoalteromonas aliena*	99,85	*Pseudoalteromonas aliena*	99,85
Mc7	*Pseudoalteromonas aliena*	*Pseudoalteromonas aliena*	99,85	*Pseudoalteromonas aliena*	99,7
Mc8	*Shewanella denitrificans*	*Shewanella denitrificans*	100	*Shewanella denitrificans*	100

Mc9	*Pseudomonas azotoformans*	*Pseudomonas azotoformans*	99,7	*Pseudomonas azotoformans*	99,85
Mc10	*Phaeobacter sp.*	*Phaeobacter sp.*	99	*Phaeobacter sp.*	99,69
Mc11	*Pseudomonas azotoformans*	*Pseudomonas azotoformans*	99,7	*Pseudomonas azotoformans*	99,7
Mc12	*Pseudomonas japonica*	*Pseudomonas neuropathica*	89,01	*Pseudomonas japonica*	99,7
Mc13	*Pseudomonas azotoformans*	*Pseudomonas azotoformans*	99,7	*Pseudomonas azotoformans*	99,4
Mc14	*Pseudoalteromonas nigrifaciens*	*Pseudoalteromonas nigrifaciens*	99,7	*Pseudoalteromonas nigrifaciens*	100
Mc15	*Pseudomonas japonica*	*Pseudomonas japonica*	98,7	*Pseudomonas japonica*	99,85
Mb1	*Pseudomonas azotoformans*	*Pseudomonas azotoformans*	99,7	*Pseudomonas azotoformans*	99,7
Mb3a	*Pseudomonas azotoformans*	*Pseudomonas azotoformans*	99,7	*Pseudomonas azotoformans*	99,85
Mb3b	*Pseudomonas azotoformans*	*Pseudomonas azotoformans*	99,7	*Pseudomonas azotoformans*	99,85
Mb4	*Pseudomonas azotoformans*	*Pseudomonas azotoformans*	99,7	*Pseudomonas azotoformans*	99,85
Mb5	*Pseudomonas azotoformans*	*Pseudomonas azotoformans*	99,7	*Pseudomonas azotoformans*	99,85
Mb6	*Pseudomonas azotoformans*	*Pseudomonas azotoformans*	99,7	*Pseudomonas azotoformans*	99,55
Mb7	*Pseudomonas azotoformans*	*Pseudomonas azotoformans*	99,7	*Pseudomonas azotoformans*	99,85
Mb10	*Pseudomonas azotoformans*	*Pseudomonas azotoformans*	95,03	*Pseudomonas azotoformans*	99,85
Mb11	*Loktanella ponticola*	*Tateyamaria pelophila*	68,9	*Loktanella ponticola*	99,22
Mb12	*Pseudomonas azotoformans*	*Pseudomonas azotoformans*	99,7	*Pseudomonas azotoformans*	99,85
Mb13	*Psychromonas sp.*	*Psychromonas aquatilis*	98,21	*Psychromonas aquatilis*	98,5
Mb14	*Pseudomonas japonica*	*Pseudomonas japonica*	91,97	*Pseudomonas japonica*	100
Mb16	*Pseudomonas azotoformans*	*Pseudomonas azotoformans*	99,7	*Pseudomonas azotoformans*	99,85
Mb17	*Pseudomonas azotoformans*	*Pseudomonas azotoformans*	99,55	*Pseudomonas azotoformans*	99,85
Mb18	*Pseudomonas azotoformans*	*Pseudomonas azotoformans*	99,7	*Pseudomonas azotoformans*	99,7
Mb20	*Psychrobacter fozii*	*Psychrobacter fozii*	99,85	*Psychrobacter fozii*	100
Mb22	*Pseudomonas azotoformans*	*Pseudomonas azotoformans*	99,7	*Pseudomonas azotoformans*	99,7
Mb24	*Pseudomonas japonica*	*Pseudomonas japonica*	91,72	*Pseudomonas japonica*	100
Mb26	*Pseudoalteromonas nigrifaciens*	*Pseudoalteromonas nigrifaciens*	99,7	*Pseudoalteromonas nigrifaciens*	99,85
Mb27	*Pseudomonas azotoformans*	*Pseudomonas azotoformans*	98,12	*Pseudomonas azotoformans*	99,85
Mb28	*Pseudomonas azotoformans*	*Pseudomonas paracarnis*	98,28	*Pseudomonas azotoformans*	99,7

Mnt2	*Microbulbifer thermotolerans*	*Microbulbifer thermotolerans*	97,89	*Microbulbifer thermotolerans*	98,35
Mnt3	*Pseudoalteromonas nigrifaciens*	*Pseudoalteromonas nigrifaciens*	100	*Pseudoalteromonas nigrifaciens*	99,85
Mnt4	*Pseudoalteromonas aliena*	*Pseudoalteromonas aliena*	99,85	*Pseudoalteromonas aliena*	99,85
Mnc1	*Pseudomonas azotoformans*	*Pseudomonas azotoformans*	99,7	*Pseudomonas azotoformans*	99,7
Mnc2	*Pseudomonas azotoformans*	*Pseudomonas azotoformans*	99,7	*Pseudomonas azotoformans*	99,85
Mnc3	*Loktanella ponticola*	*Loktanella ponticola*	69,58	*Loktanella ponticola*	98,77
b2	*Colwellia sp.*	*Colwellia AX746195_s*	86,73	*Colwellia AX746195_s*	98,79
b4	*Colwellia sp.*	*Colwellia AX746195_s*	71,48	*Colwellia AX746195_s*	98,65
b5	*Colwellia sp.*	*Colwellia AX746195_s*	86,97	*Colwellia AX746195_s*	98,06
b7	*Pseudomonas azotoformans*	*Pseudomonas azotoformans*	99,7	*Pseudomonas azotoformans*	99,85
b8	*Sulfitobacter donghicola*	*Sulfitobacter porphyrae*	88,88	*Sulfitobacter donghicola*	99,53
b9	*Pseudomonas azotoformans*	*Pseudomonas azotoformans*	99,7	*Pseudomonas azotoformans*	99,55
b10	*Pseudomonas azotoformans*	*Pseudomonas paracarnis*	98,41	*Pseudomonas azotoformans*	99,7
b11	*Pseudoalteromonas aliena*	*Pseudoalteromonas aliena*	99,85	*Pseudoalteromonas aliena*	99,85
b12	*Pseudomonas azotoformans*	*Pseudomonas azotoformans*	98,4	*Pseudomonas azotoformans*	99,85
b13	*Vibrio sp.*	*Vibrio splendidus*	100	*Vibrio splendidus*	100
b14	*Cytobacillus firmus*	*Cytobacillus firmus*	99,7	*Cytobacillus firmus*	99,41
b15	*Pseudoalteromonas rhizosphaerae*	*Pseudoalteromonas rhizosphaerae*	99,85	*Pseudoalteromonas rhizosphaerae*	99,7
b16	*Colwellia sp.*	*Colwellia AX746195_s*	70,28	*Colwellia AX746195_s*	100
b17a	*Bacillus altitudinis*	*Bacillus altitudinis*	100	*Bacillus altitudinis*	100
b17b	*Bacillus altitudinis*	*Bacillus altitudinis*	100	*Bacillus altitudinis*	100
b19	*Colwellia sp.*	*Colwellia AX746195_s*	85,62	*Colwellia AX746195_s*	98,98
b20	*Bacillus altitudinis*	*Bacillus pumilus*	92,7	*Bacillus altitudinis*	100
b21	*Psychromonas sp.*	*Psychromonas arctica*	98,52	*Psychromonas arctica*	98,5
b22	*Vibrio sp.*	*Vibrio MCVZ_s*	99,85	*Vibrio MCVZ_s*	98,95
b23	*Sulfitobacter sp.*	*Roseobacter cerasinus*	69,67	*Sulfitobacter donghicola*	97,29
b24	*Pseudomonas azotoformans*	*Pseudomonas azotoformans*	99,84	*Pseudomonas azotoformans*	95,8

b25	*Pseudomonas azotoformans*	*Pseudomonas azotoformans*	99,7	*Pseudomonas azotoformans*	99,7
b26	*Pseudomonas japonica*	*Pseudomonas japonica*	88,08	*Pseudomonas japonica*	100
b27	*Pseudomonas azotoformans*	*Pseudomonas azotoformans*	99,7	*Pseudomonas azotoformans*	99,7
b28	*Pseudomonas azotoformans*	*Pseudomonas azotoformans*	99,7	*Pseudomonas azotoformans*	99,7
b29	*Phaeobacter sp.*	*Phaeobacter sp.*	98,77	*Phaeobacter sp.*	99,07
b30	*Pseudomonas azotoformans*	*Pseudomonas azotoformans*	99,7	*Pseudomonas azotoformans*	96,38
b31	*Pseudomonas azotoformans*	*Pseudomonas azotoformans*	99,5	*Pseudomonas azotoformans*	94,05
b32	*Bacillus pumilus*	*LJIY_s (Bacillus)*	90,13	*Bacillus pumilus*	99,85
b33	*Pseudomonas azotoformans*	*Pseudomonas azotoformans*	99,7	*Pseudomonas azotoformans*	99,85
b34	*Psychrobacter nivimaris*	*Psychrobacter nivimaris*	99,85	*Psychrobacter nivimaris*	99,7
b35	*Cobetia litoralis*	*Cobetia litoralis*	100	*Cobetia litoralis*	99,7
b36	*Pseudoalteromonas aliena*	*Pseudoalteromonas aliena*	99,55	*Pseudoalteromonas aliena*	99,7
b37	*Psychrobacter fozii*	*Psychrobacter fozii*	100	*Psychrobacter fozii*	100
b38	*Nioella sp.*	*AB302375_s (;Rhodobacteraceae;AB302375_g)*	68,6	*Nioella nitratireducens*	97,36
b39	*Pseudomonas azotoformans*	*Pseudomonas azotoformans*	99,7	*Pseudomonas azotoformans*	99,85
b40	*Ruegeria meonggei*	*Ruegeria meonggei*	68,37	*Ruegeria meonggei*	98,04
b41	*Pseudomonas azotoformans*	*Pseudomonas azotoformans*	99,7	*Pseudomonas azotoformans*	99,85
b42	*Pseudoalteromonas aliena*	*Pseudoalteromonas aliena*	99,85	*Pseudoalteromonas aliena*	99,85
b43	*Brevibacterium frigoritolerans*	*Brevibacterium frigoritolerans*	100	*Brevibacterium frigoritolerans*	99,85
b44	*Wenyingzhuangia sp.*	*Wenyingzhuangia heitensis*	98,32	*Wenyingzhuangia heitensis*	98,32
b48	*Paracoccus yeei*	*Paracoccus yeei*	100	*Paracoccus yeei*	100
b50	*Pseudomonas azotoformans*	*Pseudomonas azotoformans*	99,7	*Pseudomonas azotoformans*	99,85
b51	*Phaeobacter sp.*	*Phaeobacter sp.*	98,91	*Phaeobacter sp.*	99,69
b54	*Sulfitobacter donghicola*	*Sulfitobacter donghicola*	95,78	*Sulfitobacter donghicola*	99,38
b56.1	*Pseudomonas japonica*	*Pseudomonas japonica*	100	*Pseudomonas japonica*	100
b57	*Pseudomonas azotoformans*	*Pseudomonas azotoformans*	99,7	*Pseudomonas azotoformans*	99,85
C1	*Vibrio sp.*	*Vibrio splendidus*	100	*Vibrio splendidus*	100

C2	*Pseudoalteromonas nigrifaciens*	*Pseudoalteromonas nigrifaciens*	100	*Pseudoalteromonas nigrifaciens*	100
C3	*Vibrio sp.*	*Vibrio splendidus*	100	*Vibrio splendidus*	100
C4	*Pseudoalteromonas rhizosphaerae*	*Pseudoalteromonas rhizosphaerae*	100	*Pseudoalteromonas rhizosphaerae*	99,85
C5	*Pseudoalteromonas nigrifaciens*	*Pseudoalteromonas nigrifaciens*	100	*Pseudoalteromonas nigrifaciens*	100
C6	*Pseudoalteromonas translucida*	*Pseudoalteromonas translucida*	100	*Pseudoalteromonas translucida*	100
C7	*Pseudomonas azotoformans*	*Pseudomonas azotoformans*	99,69	*Pseudomonas azotoformans*	99,7
C8	*Vibrio sp.*	*Vibrio splendidus*	100	*Vibrio splendidus*	100
i1	*Sulfitobacter donghicola*	*Sulfitobacter donghicola*	94,5	*Sulfitobacter donghicola*	100
i2	*Sulfitobacter donghicola*	*Sulfitobacter donghicola*	94,77	*Sulfitobacter donghicola*	100
i3	*Sulfitobacter donghicola*	*Sulfitobacter donghicola*	98,5	*Sulfitobacter donghicola*	100
i4	*Pseudomonas azotoformans*	*Pseudomonas azotoformans*	99,7	*Pseudomonas azotoformans*	99,85
i5	*Pseudomonas azotoformans*	*Pseudomonas azotoformans*	99,69	*Pseudomonas azotoformans*	99,85
i6	*Pseudomonas japonica*	*Pseudomonas japonica*	100	*Pseudomonas japonica*	100
i7	*Pseudomonas mucoides*	*Pseudomonas azotoformans*	99,7	*Pseudomonas mucoides*	84,09
i9	*Salinibacterium amurskyense*	*Salinibacterium amurskyense*	100	*Salinibacterium amurskyense*	100
N4	*Cobetia litoralis*	*Cobetia litoralis*	100	*Cobetia litoralis*	100
N5	*Cobetia amphilecti*	*Cobetia amphilecti*	100	*Cobetia amphilecti*	99,85
N6	*Cobetia amphilecti*	*Cobetia amphilecti*	100	*Cobetia amphilecti*	100
N7	*Staphylococcus sp.*	*Staphylococcus warneri*	100	*Staphylococcus warneri*	99,85
N8	*Rothia sp.*	*Rothia sp.*	99,85	*Rothia sp.*	100
N9	*Rothia sp.*	*Rothia sp.*	100	*Rothia sp.*	100
N10	*Rothia sp.*	*Rothia sp.*	100	*Rothia sp.*	99,85
N11	*Bacillus licheniformis*	*Bacillus licheniformis*	100	*Bacillus licheniformis*	100
N12	*Cobetia litoralis*	*Cobetia litoralis*	100	*Cobetia litoralis*	100
N13	*Cobetia amphilecti*	*Cobetia amphilecti*	100	*Cobetia amphilecti*	100
N14	*Cobetia amphilecti*	*Cobetia amphilecti*	100	*Cobetia amphilecti*	100
N15.1	*Priestia flexa*	*Priestia flexa*	99,25	*Priestia flexa*	100

N15.2	*Priestia flexa*	*Priestia flexa*	100	*Priestia flexa*	100
N16	*Cobetia amphilecti*	*Cobetia amphilecti*	100	*Cobetia amphilecti*	100
N17	*Cobetia amphilecti*	*Cobetia amphilecti*	100	*Cobetia amphilecti*	99,85
N18	*Dermacoccus sp.*	*Dermacoccus sp.*	99,85	*Dermacoccus sp.*	99,85
N19	*Staphylococcus sp.*	*Staphylococcus sp.*	100	*Staphylococcus epidermidis*	99,85
N20	*Cobetia amphilecti*	*Cobetia amphilecti*	100	*Cobetia amphilecti*	100
N21	*Cobetia amphilecti*	*Cobetia amphilecti*	100	*Cobetia amphilecti*	100
N22	*Cobetia litoralis*	*Cobetia litoralis*	100	*Cobetia litoralis*	100
N24	*Cellulophaga pacifica*	*Cellulophaga pacifica*	99,55	*Cellulophaga pacifica*	99,54
N26	*Cobetia amphilecti*	*Cobetia amphilecti*	100	*Cobetia amphilecti*	99,85
N27	*Cobetia amphilecti*	*Cobetia amphilecti*	100	*Cobetia amphilecti*	100
N28.1	*Pseudomonas sp.*	*Pseudomonas sp.*	99,7	*Pseudomonas sp.*	99,7
N28.2	*Pseudomonas sp.*	*Pseudomonas sp.*	99,7	*Pseudomonas sp.*	99,7
N29	*Cellulophaga algicola*	*Cellulophaga algicola*	100	*Cellulophaga algicola*	100
N30	*Cobetia amphilecti*	*Cobetia amphilecti*	100	*Cobetia amphilecti*	99,7
N31	*Cobetia litoralis*	*Cobetia litoralis*	100	*Cobetia litoralis*	100
N32	*Cobetia amphilecti*	*Cobetia amphilecti*	100	*Cobetia amphilecti*	100
N33	*Cellulophaga pacifica*	*Cellulophaga pacifica*	100	*Cellulophaga pacifica*	99,54
N34	*Rothia sp.*	*Rothia sp.*	100	*Rothia sp.*	100
N36	*Staphylococcus sp.*	*Staphylococcus saprophyticus*	96,4	*Staphylococcus sp.*	99,85
N37.1	*Bacillus licheniformis*	*Bacillus licheniformis*	100	*Bacillus licheniformis*	100
N37.2	*Bacillus licheniformis*	*Bacillus licheniformis*	100	*Bacillus licheniformis*	100
N38	*Staphylococcus sp.*	*Staphylococcus sp.*	100	*Staphylococcus sp.*	99,85
N39.1	*Pseudomonas oryzihabitans*	*Pseudomonas oryzihabitans*	100	*Pseudomonas oryzihabitans*	100
N39.2	*Pseudomonas oryzihabitans*	*Pseudomonas oryzihabitans*	100	*Pseudomonas oryzihabitans*	100
N40.1	*Cellulophaga pacifica*	*Cellulophaga pacifica*	99,7	*Cellulophaga pacifica*	99,54
N40.2	*Cellulophaga pacifica*	*Cellulophaga pacifica*	99,24	*Cellulophaga pacifica*	99,54
N41.1	*Staphylococcus sp.*	*Staphylococcus sp.*	100	*Staphylococcus sp.*	99,71

row ID	row m/z	RT	M-H+	M-Na+	Adduct	Exact Mass	SuperClass	Class	Subclass	Level 5	Pathway	Putative annotation	Chemical formula	Smiles	Organism	Source of annotation	DOI
24	83,0469	2,03	82,0395	60,0669									C3H8O			Sirius	
25	191,0673	2,03	190,0599		H+	190,063	Phenylpropanoids and polyketides	Coumarins and derivatives	Isocoumarins		Shikimates and phenylpropanoids	7-hydroxy-3, 5-dimethyl-isochromen-1-one	C11H10O3	CC1=CC2=C(C=C(C=C2C(=O)O1)O)C	Fungus	Sirius	10.1016/j.fitote.2011.10.013
27	100,0736	2,57	99,0662		H+		Organoheterocyclic compounds				Alkaloids	Azinanone (piperin-4-one)	C5H9NO			Sirius	
31	345,1403	5,41	344,1329	322,1603			Organic acids and derivatives	Carboxylic acids and derivatives	Amino acids, peptides and analogues	Amino acids and derivatives	Amino acids and peptides		C16H18B2N2O4			Sirius	
33	261,1200	5,75	260,1126		H+	260,29	Organic acids and derivatives	Carboxylic acids and derivatives	Amino acids, peptides, and analogues	Amino acids and derivatives	Amino acids and peptides	Cyclo(Pro-Tyr)	C14H16N2O3	C1CC2C(=O)NC(C(=O)N2C1)CC3=CC=C(C=C3)O	Fungus (Aspergillus versicolor)	Sirius	10.3390/md12084326
34	211,1410	6,88	210,1336		H+	210,13	Organic acids and derivatives	Carboxylic acids and derivatives	Amino acids, peptides, and analogues	Amino acids and derivatives	Amino acids and peptides	Cyclo(Leu-Pro)	C11H18N2O2	CC(C)CC1C(=O)N2CCCC2C(=O)N1	Bacteria	GNPS	10.1021/np030233e
36	245,1251	7,46	244,1177		H+	244,29	Organic acids and derivatives	Carboxylic acids and derivatives	Amino acids, peptides, and analogues	Amino acids and derivatives	Amino acids and peptides	Cyclo(Phe-Pro)	C14H16N2O2	C1CC2C(=O)NC(C(=O)N2C1)CC3=CC=CC=C3	Bacteria	GNPS	10.1021/np030233e
38	261,1566	8,89	260,1492		H+	260,33	Organic acids and derivatives	Carboxylic acids and derivatives	Amino acids, peptides, and analogues	Amino acids and derivatives	Amino acids and peptides	Cyclo(Phe-Leu)	C15H20N2O2	CC(C)CC1C(=O)NC(C(=O)N1)CC2=CC=CC=C2	Fungus	GNPS	10.1021/np060315d
39	288,2864	9,73	287,2790		H+		Lipid and lipid-like molecules	Fatty acyls	Fatty amides		Fatty acids		C13H33N7			Sirius	
42	285,0729	10,19	284,0655		H+	284,2641	Phenylpropanoids and polyketides	Isoflavonoids	O-methylated isoflavonoids		Shikimates and Phenylpropanoids	Glycetein	C16H12O5	COC1=C(C=C2C(=C1)C(=O)C(=CO2)C3=CC=C(C=C3)O)O	Fungus	GNPS	10.1021/np100902f
43	255,0622	10,48	254,0548		H+	254,2381	Phenylpropanoids and polyketides	Isoflavonoids	Isoflav-2-enes	Isoflavones	Shikimates and phenylpropanoids	daidzein	C15H10O4	C1=CC(=CC=C1C2=COC3=C(C2=O)C=CC(=C3)O)O	Bacteria	GNPS	10.1515/znb-2003-0713
44	316,3168	10,68	315,3094		H+		Lipid and lipid-like molecules	Fatty acyls	Fatty amides		Fatty acids		C15H37N7			Sirius	
45	285,2045	11,27	284,1971		H+	284,39	Lipid and lipid-like molecules	Fatty acyls	Fatty acids and conjugated	Long-chain fatty acids	Fatty acids	7-Hexadecenedioic acid	C16H28O4	C(CCCC=CCCCCC(=O)O)CCCC(=O)O		GNPS	
48	332,3276	11,70	331,3202		H+	331,5	Organic nitrogen compounds	Organonitrogen compounds	Amines	Tertiary amines	Fatty acids	N-butyl-N-[1-[2-(dibutylamino)ethyl-hydroxyamino]butan-2-yl]hydroxylamine	C18H41N3O2	CCCCN(CCCC)CCN(CC(CC)N(CCCC)O)O		Sirius	
49	271,0584	11,88	270,0510		H+	270,2375	Phenylpropanoids and polyketides	Isoflavonoids	Isoflav-2-enes	Isoflavones	Shikimates and phenylpropanoids	Prunetol	C13H12O5	C1=CC(=CC=C1C2=COC3=CC(=CC(=C3C2=O)O)O)O	Bacteria	GNPS	10.1080/14786419.2016.1269100

49	271,0584	11,88	270,0510		H+	270,2375	Phenylpropanoids and polyketides	Isoflavonoids	Isoflav-2-enes	Isoflavones	Shikimates and phenylpropanoids	Prunetol	C13H12O5	C1=CC(=CC=C1C2=COC3=CC(=CC(=C3C2=O)O)O)O	Bacteria	GNPS	10.1080/14786419.2016.1269100
50	371,3218	12,65	370,3144		H+		Lipid and lipid-like molecules	Prenol lipids					C22H38BN3O			Sirius	
51	288,2522	12,77	287,2448		H+	287,44	Organic acids and derivatives	Carboxylic acids and derivatives	Amino acids, peptides and analogues	Alpha amino acids and derivatives	Fatty acids	N-dodecyl-N-(2-hydroxyethyl)glycine	C16H33NO3	CCCCCCCCCCCCN(CCO)CC(=O)O		Sirius	
52	331,2295	13,07	330,2221		H+	330,4	Organic oxygen compounds	Organooxygen compounds	Carbohydrates and carbohydrate conjugated	Sugar acids and derivatives	Fatty acids	(2E,10R)-10-[(3,6-dideoxy-alpha-L-arabino-hexopyranosyl)oxy]undec-2-enoic acid	C17H30O6	CC1C(CC(C(O1)OC(C)CCCCCCC=CC(=O)O)O)O		GNPS	
53	244,2263	13,10	243,2189		H+	243,39	Lipid and lipid-like molecules	Fatty acyls	Fatty amides		Fatty acids	N-(2-Hydroxyethyl)dodecanamide	C14H29NO2	CCCCCCCCCCCC(=O)NCCO	Fungus	GNPS	10.1111/nph.15672
54	286,2367	13,52	285,2293		H+	285,21	Organic acids and derivatives	Carboxylic acids and derivatives	Amino acids, peptides and analogues		Amino acids and peptides	Acetyl-isoleucine-leucinamide	C14H27N3O3	CCC(C)C(C(=O)NC(CC(C)C)C(=O)N)NC(=O)C	Bacteria	Sirius	10.1016/j.tet.2018.03.028
55	365,1348	14,07	364,1274	342,1548													
58	72,0434	14,37	71,0360	49,0634													
59	445,3053	15,15	444,2979	422,3253													
62	338,3400	16,51	337,3326		H+		Lipid and lipid-like molecules	Fatty acyls	Fatty acids and conjugated		Fatty acids		C22H43NO			Sirius	
64	282,2776	16,94	281,2702		H+	281,48	Lipid and lipid-like molecules	Fatty acyls	Fatty amides		Fatty acids	Octadec-8-enimidic acid	C18H35NO	CCCCCCCCC=CCCCCCCC(=N)O	Eukaryota	Sirius	10.1515/znc-2002-3-407
66	284,2928	17,61	283,2854		H+	283,5	Lipid and lipid-like molecules	Fatty acyls	Fatty amides		Fatty acids	Isooctadecanamide	C18H37NO	CC(C)CCCCCCCCCCCCCC(=O)N		Sirius	
68	368,3872	18,08	367,3798	345,4072			Lipid and lipid-like molecules	Fatty acyls	Fatty amides		Fatty acids					Sirius	
76	610,1783	19,15		587,1983	Na+	587,23	Phenylpropanoids and polyketides	Anthraquinones and anthrones			Polyketides	Nothramicin	C30H37NO11	CC1C(C(CC(O1)OC2C(C(C(C3=CC4=C(C(=C23)O)C(=O)C5=C(C=CC(=C5C4=O)OC)O)O)(C)O)OC)(C)N(C)C)O	Bacteria (Nocardia sp.)	Npatlas	10.7164/antibiotics.51.130
80	312,3248	19,31	311,3174		H+	311,5	Lipid and lipid-like molecules	Fatty acyls	Fatty amides		Fatty acids	Octadecananmide	C20H41NO	CCCCCCCCCCCCCCCCCC(=O)NCC		Sirius	
81	61,0063	19,44	59,9989	38,0263													
82	70,0117	19,44	69,0043	47,0317									F2HNO			Sirius	
83	79,0171	19,44	78,0097	56,0371									C5H2O			Sirius	
85	90,5252	19,44	89,5178	67,5452													
95	62,0216	0,72	61,0142	39,0416									CH4BCl			Sirius	
96	73,5297	0,82	72,5223	50,5497													
97	82,5348	0,72	81,5274	59,5548													
98	363,1092	1,03	362,1018	340,1292			Organoheterocyclic compounds	Indoles and derivatives			Alkaloids		C19H14N4O4			Sirius	
101	86,0941	1,35	85,0867	63,1141													

107	227,1358	5,70	226,1284		H+	226,27	Organic acids and derivatives	Carboxylic acids and derivatives	Amino acids, peptides, and analogues	Amino acids and derivatives	Amino acids and peptides	Cyclo(Leu-trans-4-hydroxy-Pro)	C11H18N2O3	CC(C)CC1C(=O)N2CC(CC2C(=O)N1)O	Fungus (Aspergillus sp.)	Sirius	10.1590/S0103-50532005000800026
108	185,1256	5,95		162,1456	Na+	162,23	Organic nitrogen compounds	Organonitrogen compounds	Amines	Alkanolamines	Fatty amides	Aminopropyldiethanolamine	C7H18N2O2	C(CN)CN(CCO)CCO		Sirius	
109	213,1564	7,71	212,1490		H+	212,15	Organic acids and derivatives	Carboxylic acids and derivatives	Amino acids, peptides, and analogues	Amino acids and derivatives	Amino acids and peptides	Cyclo(Val-Leu)	C11H20N2O2	CC(C)CC1C(=O)NC(C(=O)N1)C(C)C	Fungus (Penicillium sp.)	Npatlas	10.1080/10286020.2013.780349
110	295,1406	9,29		272,1606	Na+	272,34	Organic acids and derivatives	Carboxylic acids and derivatives	Amino acids, peptides, and analogues		Amino acids and peptides	(6R,3Z)-3-benzylidene-6-isobutyl-1-methylpiperazine-2,5-dione	C16H20N2O2	CC(C)CC1C(=O)NC(=CC2=CC=CC=C2)C(=O)N1C	Bacteria	Npatlas	10.1080/14786419.2015.1045509
111	216,1925	9,58	215,1851		H+	216,34	Organic acids and derivatives	Carboxylic acids and derivatives	Amino acids, peptides, and analogues		Fatty acids		C12H25NO2			Sirius	
112	558,3223	9,87		535,3423	Na+	535,328	Organoheterocyclic compounds	Prenol lipids	Diterpenoids		Alkaloids	Drechmerin C	C33H45NO5	CC(=CCOC(C)(C)C1CC(C2(C3CCC4CC5=C(C4(C3(CCC2O1)C)C)NC6=CC=CC=C56)C(=O)O)O)C	Fungus	Npatlas	10.1016/j.phytochem.2018.01.010
114	556,3636	11,30	555,3562		H+	555,31	Organoheterocyclic compounds	Prenol lipids	Diterpenoids		Terpenoids	Drechmerin F	C32H45NO7	CC12CCC3C4(C1(CCC5C2(C6=C(C5)C7=C(C=CC=C7N6)CC(C(C)(C)O)O)C)O)C(O4)C(C(O3)C(C)(C)O)O	Fungus	Npatlas	10.1016/j.phytochem.2018.01.010
116	355,2584	12,01		332,2784	Na+	332,5	Lipid and lipid-like molecules	Steroids and steroid derivatives	Bile acids, alcohols and derivatives	Hydroxy bile acids, alcohols and derivatives	Terpenoids	(8S,9S,10R,13S,14S,17R)-17-[(2S)-1-hydroxypropan-2-yl]-10,13-dimethyl-2,3,4,7,8,9,11,12,14,15,16,17-dodecahydro-1H-cyclopenta[a]phenanthren-3-ol	C22H36O2	CC(CO)C1CCC2C1(CCC3C2CC=C4C3(CCC(C4)O)C)C		Sirius	
117	373,2685	12,01		350,2885	Na+	350,5	Lipid and lipid-like molecules	Steroids and steroid derivatives	Bile acids, alcohols and derivatives	Hydroxy bile acids, alcohols and derivatives	Terpenoids	(3R,10S,12S,13S,17R)-17-[(2S)-1-hydroxypropan-2-yl]-10,13-dimethyl-2,3,4,5,6,7,8,9,11,12,14,15,16,17-tetradecahydro-1H-cyclopenta[a]phenanthrene-3,12-diol	C22H38O3	CC(CO)C1CCC2C1(C(CC3C2CCC4C3(CCC(C4)O)C)O)C		Sirius	
119	415,2062	12,44			M-H2O+H+	415,211	Organoheterocyclic compounds	Naphthofurans			Diterpenoids	Gelomulide N	C24H32O7	CC1=C2C(CC3C4(C(CCC35C2O5)C(C(CC4OC(=O)C)OC(=O)C)(C)C)C)OC1=O	Eukaryota	GNPS	
120	288,2489	12,78	287,2415		H+	287,44	Lipid and lipid-like molecules	Fatty acyls	Fatty amides		Fatty acids	N,N-bis(2-hydroxyethyl)dodecanamide	C12H29N7O	CCCCCCCCCCC(=O)N(CCO)CCO		GNPS	

121	450,3155	12,90	449,3081		H+	449,6	Lipid and lipid-like molecules	Steroids and steroid derivatives	Bile acids, alcohols and derivatives	Glycinated bile acids and derivatives	Terpenoids	cyclopenta[a]phenanthren-17-yl]pentanoyl]amino]acetic acid	C26H43NO5	CC(CCC(=O)NCC(=O)O)C1CCC2C1(C(CC3C2CCC4C3(CCC(C4)O)C)O)C	Bacteria	Sirius	10.3389/fmicb.2018.01587
123	244,2231	13,10	243,2157		H+	243,39	Lipid and lipid-like molecules	Fatty acyls	Fatty amides		Fatty acids	N-(2-Hydroxyethyl)dodecanamide	C14H29NO2	CCCCCCCCCCCC(=O)NCCO	Fungus	GNPS	10.1111/nph.15672
127	365,1308	14,06	364,1234	342,1508													
131	338,3376	16,51	337,3302		H+		Lipid and lipid-like molecules	Fatty acyls	Fatty acids and conjugated		Fatty acids		C22H43NO			Sirius	
132	282,2752	16,94	281,2678		H+	281,48	Lipid and lipid-like molecules	Fatty acyls	Fatty amides		Fatty acids	9-Octadecenamide	C18H35NO	CCCCCCCCC=CCCCCCCCC(=N)O	Eukaryota	Sirius	10.1002/pmic.201700366
135	360,0644	18,30	359,0570	337,0844													
136	338,0830	18,26		315,1030	Na+								C13H17NO8			Sirius	
142	61,0054	19,42	59,9980	38,0254													
143	70,0106	19,43	69,0032	47,0306									F2HNO			Sirius	
144	79,0160	19,42	78,0086	56,0360									C5H2O			Sirius	
145	81,5187	19,42	80,5113	58,5387													
147	90,5240	19,42	89,5166	67,5440													
157	67,0193	0,73	66,0119	44,0393													
158	110,0064	0,73	108,9990		H+								CH3NO5			Sirius	
161	282,0832	0,99		259,1032	Na+		Organic nitrogen compounds	Organonitrogen compounds	Hydrazines and derivatives		Carbohydrates		C8H13N5O5			Sirius	
162	527,1519	1,00		504,1719	Na+	504,4	Organic oxygen compounds	Organooxygen compounds	Carbohydrates and carbohydrate conjugated	Oligosaccharides	Carbohydrates	Maltotriose	C18H32O16	C(C1C(C(C(C(O1)OC2C(OC(C(C2O)O)OC3C(OC(C(C3O)O)O)CO)CO)O)O)O)O	Eukaryota	Sirius	10.1371/journal.pntd.0001618
163	160,0730	1,41	159,0656		H+	159,068	Organoheterocyclic compounds	Indoles and derivatives	Indoles		Alkaloids	2H-Indol-2-one	C10H9NO3	C/C=C\1/C2=CC=CC=C2NC1=O	Fungus	Sirius	10.1007/bf02040097
167	235,1042	4,46	234,0968		H+	234,0892	Benzenoids	Benzene and substituted derivatives	Benzoic acids and derivatives	Hydroxybenzoic acid and derivatives	Polyketides	5,7-dihydroxy-6-(3-methylbut-2-enyl)isobenzofuran-1(3H)-one	C13H14O4	CC(=CCC1=C(C=C2COC(=O)C2=C1O)O)C	Fungus	Sirius	10.1002/cbdv.200690024
168	169,0731	5,39	168,0657		H+	168,195	Organoheterocyclic compounds	Indoles and derivatives	Carbolin alkaloids	β-carboline	Alkaloids	β-carboline	C11H8N2	C1=CC=C2C(=C1)C3=C(N2)C=NC=C3	Bacteria	GNPS	10.1271/bbb1961.51.921
171	311,1349	7,95	310,1275		H+	310,3478	Organic acids and derivatives	Carboxylic acids and derivatives	Amino acids, peptides and analogues	Amino acids and derivatives	Amino acids and peptides	Cyclo(Tyr-Phe)	C18H18N2O3	C1=CC=C(C=C1)CC2C(=O)NC(C(=O)N2)CC3=CC=C(C=C3)O	Bacteria (Bacillus sp.)	Sirius	10.1007/s10600-009-9270-9
172	227,1722	8,74	226,1648		H+	226,168	Organic acids and derivatives	Carboxylic acids and derivatives	Amino acids, peptides, and analogues	Amino acids and derivatives	Amino acids and peptides	Cyclo(Leu-Ile)	C12H22N2O2	CCC(C)C1C(=O)NC(C(=O)N1)CC(C)C	Fungus	Npatlas	10.1016/s0031-9422(01)00470-8
173	177,0515	11,37		154,0715	Na+	154,16	Benzenoids	Phenols	1-hydroxy-2-unsubstituted benzenoids		Shikimates and phenylpropanoids	Phomoaspardiol	C8H10O3	C1=CC(=CC=C1C(CO)O)O	Fungus	Sirius	10.1007/s10600-018-2390-3

176	233,1499	12,82		210,1699	Na+		Lipid and lipid-like molecules	Fatty acyls	Fatty acyls		Fatty acids		C13H22O2			Sirius	
177	286,2332	13,53	285,2258		H+	285,21	Organic acids and derivatives	Carboxylic acids and derivatives	Amino acids, peptides and analogues		Amino acids and peptides	Acetyl-isoleucine-leucinamide	C14H27N3O3	CCC(C)C(C(=O)NC(CC(C)C)C(=O)N)NC(=O)C	Bacteria	Sirius	10.1016/j.tet.2018.03.028
179	341,2616	15,29	340,2542		H+		Lipid and lipid-like molecules	Fatty acyls	Fatty acyls		polyketides		C22H28O3			Sirius	
183	338,3373	17,03	337,3299		H+	337,6	Lipid and lipid-like molecules	Fatty acyls	Fatty amides		Fatty acids	13-Docosenamide	C22H43NO	CCCCCCCCC=CCCCCCCCCCCCC(=O)N	Bacteria	Sirius	10.3389/fmicb.2017.02704
186	536,1593	18,65	535,1519		H+	535,147	Benzenoids	Anthraquinones and anthrones	Anthraquinones		Polyketides	Actinoplanone D	C28H25NO10	CC1=CC2=CC3=C(C4=C5C(C3)OCOC5=C6C(=C4O)C(=O)C7=C(O6)C(C(CC7OC)O)OC)C(=C2C(=O)N1)O	Bacteria	Npatlas	10.7164/antibiotics.41.741
191	325,1091	1,01	324,1017		H+								C19H16O5			Sirius	
192	247,1404	8,22		224,1604	Na+	224,152	Organoheterocyclic compounds	Diazines	Pyrazines		Amino acids and peptides	Aspergillic acid	C12H20N2O2	CCC(C)C1=CN=C(C(=O)N1O)CC(C)C	Fungus (Aspergillus sp.)	Npatlas	10.5281/zenodo.3959483
193	356,2136	8,35	355,2062	333,2336			Organic acids and derivatives	Carboxylic acids and derivatives	Amino acids, peptides and analogues	Amino acids and derivatives	Amino acids and peptides		C15H31N3O5			Sirius	
194	390,1972	8,84		367,2172	Na+	367,18	Organic acids and derivatives	Carboxylic acids and derivatives	Amino acids, peptides and analogues		Amino acids and peptides	Brevianamide E	C21H25N3O3	CC(C)(C=C)C12C(CC3N1C(=O)C4CCCN4C3=O)(C5=CC=CC=C5N2)O	Fungus (Penicillium sp.)	Npatlas	10.1016/0040-4020(72)80014-0
197	466,3122	11,09		443,3322	Na+		Lipid and lipid-like molecules	Steroids and steroid derivatives			Terpenoids		C24H45NO6			Sirius	
199	373,2700	12,01		350,2900	Na+	350,5	Lipid and lipid-like molecules	Steroids and steroid derivatives	Bile acids, alcohols and derivatives	Hydroxy bile acids, alcohols and derivatives	Terpenoids	(3R,10S,12S,13S,17R)-17-[(2S)-1-hydroxypropan-2-yl]-10,13-dimethyl-2,3,4,5,6,7,8,9,11,12,14,15,16,17-tetradecahydro-1H-cyclopenta[a]phenanthrene-3,12-diol	C22H38O3	CC(CO)C1CCC2C1(C(CC3C2CCC4C3(CCC(C4)O)C)O)C		Sirius	
200	943,5159	12,34	942,5085		H+	943,1	Lipid and lipid-like molecules	Prenol lipids	Terpene glycosides	Triterpene glycosides	Terpenoids	Soyasaponin I	C48H78O18	CC1C(C(C(C(O1)OC2C(C(C(OC2OC3C(C(C(OC3OC4CCC5(C(C4(C)CO)CCC6(C5CC=C7C6(CCC8(C7CC(CC8O)(C)C)C)C)C)C)C(=O)O)O)O)CO)O)O)O)O	Eukaryota	Sirius	

201	450,3160	12,88	449,3086		H+	449,6	Lipid and lipid-like molecules	Steroids and steroid derivatives	Bile acids, alcohols and derivatives	Glycinated bile acids and derivatives	Terpenoids	cyclopenta[a]phenanthren-17-yl]pentanoyl]amino]acetic acid	C26H43NO5	CC(CCC(=O)NCC(=O)O)C1CCC2C1(C(CC3C2CCC4C3(CCC(C4)O)C)O)C	Bacteria	Sirius	10.3389/fmicb.2018.01587
203	244,2239	13,10	243,2165		H+	243,39	Lipid and lipid-like molecules	Fatty acyls	Fatty amides		Fatty acids	N-(2-Hydroxyethyl)dodecanamide	C14H29NO2	CCCCCCCCCCCC(=O)NCCO	Fungus	GNPS	10.1111/nph.15672
206	399,3537	14,30		376,3737	Na+		Lipid and lipid-like molecules	Prenol lipids					C26H44BO2			Sirius	
211	361,3533	17,00	360,3459	338,3733													
216	241,2001	7,22	240,1927		H+								C12H24N4O			Sirius	
218	289,0820	11,18		266,1020	Na+	266,0943	Organoheterocyclic compounds	Dihydrofurans	Furanones	Butenolides	Polyketides	Microperfuranone	C17H14O3	C1=CC=C(C=C1)CC2=C(C(=O)OC2O)C3=CC=CC=C3	Fungus (Aspergillus)	Sirius	10.1248/cpb.54.550
220	470,4138	13,65		447,4338	Na+		Lipid and lipid-like molecules				Alkaloids		C32H35B			Sirius	
221	394,3425	17,40	393,3351	371,3625	Na+		Benzenoids	Benzene and substituted derivatives			Alkaloids		C26H45N			Sirius	
223	368,3844	18,09	367,3770	345,4044			Lipid and lipid-like molecules	Fatty acyls	Fatty amides		Fatty acids					Sirius	
229	188,0521	3,15		165,0721	Na+	165,14	Organic acids and derivatives	Carboxylic acids and derivatives	Amino acids, peptides and analogues		Amino acids and peptides	2-amino-3,4,5-trihydroxypentanoic acid	C5H11NO5	C(C(C(C(C(=O)O)N)O)O)O		Sirius	
231	197,1255	5,21	196,1181		H+	196,12	Organic acids and derivatives	Carboxylic acids and derivatives	Amino acids, peptides, and analogues	Amino acids and derivatives	Amino acids and peptides	Cyclo(Pro-Val)	C10H16N2O2	C1CC(N(C1)CC(CO)N)CO	Fungus (Aspergillus sp.)	GNPS	10.7164/antibiotics.54.179
232	227,1355	5,70	226,1281		H+	226,27	Organic acids and derivatives	Carboxylic acids and derivatives	Amino acids, peptides, and analogues	Amino acids and derivatives	Amino acids and peptides	Cyclo(Leu-trans-4-hydroxy-Pro)	C11H18N2O3	CC(C)CC1C(=O)N2CC(CC2C(=O)N1)O	Fungus (Aspergillus sp.)	Sirius	10.1590/S0103-50532005000800026
233	261,1195	5,75	260,1121		H+	260,29	Organic acids and derivatives	Carboxylic acids and derivatives	Amino acids, peptides, and analogues	Amino acids and derivatives	Amino acids and peptides	Cyclo(Pro-Tyr)	C14H16N2O3	C1CC2C(=O)NC(C(=O)N2C1)CC3=CC=C(C=C3)O	Fungus (Aspergillus versicolor)	Sirius	10.3390/md12084326
234	185,1252	5,95	184,1178		H+	184,12	Organic acids and derivatives	Carboxylic acids and derivatives	Amino acids, peptides, and analogues	Amino acids and derivatives	Amino acids and peptides	Cyclo(Ala-Ile)	C9H16N2O2	CCC(C)C1C(=O)NC(C(=O)N1)C	Fungus (Aspergillus sp.)	Npatlas	10.1016/j.jbiotec.2004.07.008
235	192,0621	6,22	191,0547		H+	191,18	Organoheterocyclic compounds	Indoles and derivatives	Indolecarboxylic acids and derivatives		Alkaloids	1-hydroxymethylindole-3-carboxylic acid	C10H9NO3	C1=CC=C2C(=C1)C(=CN2CO)C(=O)O	Bacteria	Sirius	10.1016/j.phytol.2013.06.007
236	211,1405	6,88	210,1331		H+	210,13	Organic acids and derivatives	Carboxylic acids and derivatives	Amino acids, peptides, and analogues	Amino acids and derivatives	Amino acids and peptides	Cyclo(Leu-Pro)	C11H18N2O2	CC(C)CC1C(=O)N2CCCC2C(=O)N1	Bacteria	GNPS	10.1021/np030233e
237	342,1977	7,47	341,1903		H+	341,17	Organic acids and derivatives	Carboxylic acids and derivatives	Amino acids, peptides, and analogues		Amino acids and peptides	14-hydroxyterezine D	C19H23N3O3	CC1C(=O)N(C(C(=O)N1)CC2=CNC3=C(C=CC=C23)CC=C(C)C)O	Fungus (Aspergillus sp.)	Npatlas	10.1021/np700737g

238	190,0465	7,95		167,0665	Na+	167,058	Benzenoids	Benzene and substitudes derivatives	Nitrobenzenes		Shikimates and phenylpropanoids	Benzenemethanol	C8H9NO3	CC1=C(C=C(C=C1)[N+](=O)[O-])CO	Fungus	Sirius	10.1080/10286020600979753
239	356,2132	8,17	355,2058	333,2332			Organic acids and derivatives	Carboxylic acids and derivatives	Amino acids, peptides and analogues	Amino acids and derivatives	Amino acids and peptides		C15H31N3O5			Sirius	
243	101,0009	1,11	99,9935		H+								C4H3ClN			Sirius	
245	282,2176	16,84		259,2376	Na+	259,437	Organoheterocyclic compounds	Quinolines and derivatives	Hydroquinolines		Alkaloids	1,2,3,4-tétrahydro-5-nonylquinoline	C18H29N	CCCCCCCCC1=C2CCCNC2=CC=C1	Bacteria	Npatlas	10.1021/acs.orglett.5b00607
246	663,4458	18,56	662,4384		H+	662,9	Lipid and lipid-like molecules	Prenol lipids	Triterpenoids		Terpenoids	6alpha-(6-O-Acetyl-beta-D-glucopyranosyloxy)dammara-20,24-diene-3beta,12beta-diol	C38H62O9	CC(=CCCC(=C)C1CCC2(C1C(CC3C2(CC(C4C3(CCC(C4(C)C)O)C)OC5C(C(C(C(O5)COC(=O)C)O)O)O)C)O)C)C		Sirius	
249	338,3374	18,70	337,3300	315,3574			Lipid and lipid-like molecules	Fatty acyls	Fatty amides		Fatty acids					Sirius	
270	62,0218	0,71	61,0144	39,0418													
272	90,9742	0,90	89,9668	67,9942													
273	203,0491	0,99		180,0691	Na+	180,16	Organic oxygen compounds	Organooxygen compounds	Carbohydrates and carbohydrate conjugated	Monosaccharides	Carbohydrates	Glucose	C6H12O6	OCC1OC(O)C(O)C(O)C1O		Sirius	
276	365,1007	0,98		342,1207	Na+	342,38	Organic acids and derivatives	Carboxylic acids and derivatives	Amino acids, peptides and analogues		Carbohydrates	(2R,3R,4S,5S)-2-(2,6-diaminopurin-9-yl)-5-(2-hydroxyethylsulfanylmethyl)oxolane-3,4-diol	C12H18N6O4S	C1=NC2=C(N=C(N=C2N1C3C(C(C(O3)CSCCO)O)O)N)N		Sirius	
280	197,1246	5,20	196,1172		H+	196,12	Organic acids and derivatives	Carboxylic acids and derivatives	Amino acids, peptides, and analogues	Amino acids and derivatives	Amino acids and peptides	Cyclo(Pro-Val)	C10H16N2O2	C1CC(N(C1)CC(CO)N)CO	Fungus (Aspergillus sp.)	GNPS	10.7164/antibiotics.54.179
281	227,1347	5,70	226,1273		H+	226,27	Organic acids and derivatives	Carboxylic acids and derivatives	Amino acids, peptides, and analogues	Amino acids and derivatives	Amino acids and peptides	Cyclo(Leu-trans-4-hydroxy-Pro)	C11H18N2O3	CC(C)CC1C(=O)N2CC(CC2C(=O)N1)O	Fungus (Aspergillus sp.)	Sirius	10.1590/S0103-50532005000800026
282	261,1188	5,75	260,1114		H+	260,29	Organic acids and derivatives	Carboxylic acids and derivatives	Amino acids, peptides, and analogues	Amino acids and derivatives	Amino acids and peptides	Cyclo(Pro-Tyr)	C14H16N2O3	C1CC2C(=O)NC(C(=O)N2C1)CC3=CC=C(C=C3)O	Fungus (Aspergillus versicolor)	Sirius	10.3390/md12084326
283	185,1247	5,95	184,1173		H+	184,12	Organic acids and derivatives	Carboxylic acids and derivatives	Amino acids, peptides, and analogues	Amino acids and derivatives	Amino acids and peptides	Cyclo(Ala-Ile)	C9H16N2O2	CCC(C)C1C(=O)NC(C(=O)N1)C	Fungus (Aspergillus sp.)	Npatlas	10.1016/j.jbiotec.2004.07.008
286	227,1716	8,74	226,1642		H+	226,168	Organic acids and derivatives	Carboxylic acids and derivatives	Amino acids, peptides, and analogues	Amino acids and derivatives	Amino acids and peptides	Cyclo(Leu-Ile)	C12H22N2O2	CCC(C)C1C(=O)NC(C(=O)N1)CC(C)C	Fungus	Npatlas	10.1016/s0031-9422(01)00470-8
289	285,0720	10,19	284,0646		H+	284,2641	Phenylpropanoids and polyketides	Isoflavonoids	O-methylated isoflavonoids		Shikimates and Phenylpropanoids	Kakkatin	C16H12O5	COC1=C(C=C2C(=C1)OC=C(C2=O)C3=CC=C(C=C3)O)O	Bacteria	Sirius	10.1515/znb-2003-0713

290	466,3105	11,08		443,3305	Na+		Lipid and lipid-like molecules	Steroids and steroid derivatives			Terpenoids		C24H45NO6			Sirius	
292	373,2691	12,01		350,2891	Na+	350,5	Lipid and lipid-like molecules	Steroids and steroid derivatives	Bile acids, alcohols and derivatives	Hydroxy bile acids, alcohols and derivatives	Terpenoids	(3R,10S,12S,13S,17R)-17-[(2S)-1-hydroxypropan-2-yl]-10,13-dimethyl-2,3,4,5,6,7,8,9,11,12,14,15,16,17-tetradecahydro-1H-cyclopenta[a]phenanthrene-3,12-diol	C22H38O3	CC(CO)C1CCC2C1(C(CC3C2CCC4C3(CCC(C4)O)C)O)C		Sirius	
297	470,4150	13,65		447,4350	Na+		Lipid and lipid-like molecules				Alkaloids		C32H35B			Sirius	
299	399,3526	14,31		376,3726	Na+		Lipid and lipid-like molecules	Prenol lipids					C26H44BO2			Sirius	
302	394,3412	17,40	393,3338	371,3612	Na+		Benzenoids	Benzene and substituted derivatives			Alkaloids		C26H45N			Sirius	
303	408,3209	17,40		385,3409	Na+	385,6	Organic acids and derivatives	Carboxylic acids and derivatives	Amino acids, peptides and analogues	Amino acids and derivatives	Alkaloids	N-[2-[2-methoxyethyl-[(1-methylpyrrol-2-yl)methyl]amino]-2-oxoethyl]-N-propylnonanamide	C23H41N3O3	CCCCCCCCC(=O)N(CCC)CC(=O)N(CCOC)CC1=CC=CN1C		Sirius	
305	368,3835	18,08	367,3761	345,4035			Lipid and lipid-like molecules	Fatty acyls	Fatty amides		Fatty acids					Sirius	
306	338,0824	18,23		315,1024	Na+								C13H17NO8			Sirius	
307	360,0643	18,23	359,0569	337,0843													
310	536,1581	18,64	535,1507		H+	535,147	Organoheterocyclic compounds	Benzopyrans	1-benzopyrans	Xanthenes	Polyketides	Simaomicin	C28H25NO10	CC1=CC2=C(C(=C3C4=C2OCOC4CC5=C3C(=C6C(=C5OC)OC7=C(C6=O)C(CCC7O)O)O)O)C(=O)N1C	Bacteria	Npatlas	10.7164/antibiotics.43.1059
311	684,1941	18,76	683,1867		H+	683,7	Organic acids and derivatives	Carboxylic acids and derivatives	Pentacarboxylic acids and derivatives		Carbohydrates		C27H41NO15S2			Sirius	
324	68,9913	20,28	67,9839	46,0113													
328	434,1767	7,72	433,1693		H+	433,5	Organoheterocyclic compounds	Indoles and derivatives	Pyrrolindoles	Pyridoindolones	Alkaloids	Meleagrin	C23H23N5O4	CC(C)(C=C)C12C=C(C(=O)N3C1(NC(=O)C3=CC4=CN=CN4)N(C5=CC=CC=C25)OC)O	Fungus (Penicillium sp.)	GNPS	10.3923/pjbs.2014.667.674
329	390,1874	8,13	389,1800		H+	389,4	Organoheterocyclic compounds	Indoles and derivatives	Pyrrolindoles	Pyridoindolones	Alkaloids	Roquefortine C	C22H23N5O2	CC(C)(C=C)C12CC3C(=O)NC(=CC4=CN=CN4)C(=O)N3C1NC5=CC=CC=C25	Fungus (Penicillium sp.)	Sirius	10.1016/j.tet.2005.05.026
330	334,1503	9,39		311,1703	Na+	311,1634	Organoheterocyclic compounds	Indoles and derivatives	Indoles	3-alkyindoles	Amino acids and peptides	Trypilepyrazinol	C18H21N3O2	CCC(C)C1=NC(=C(NC1=O)CC2=CNC3=CC=CC=C32)OC	Fungus (Penicillium sp.)	Npatlas	10.3390/molecules24152821

331	513,2070	10,49		490,2270	Na+	490,5	Organic acids and derivatives	Carboxylic acids and derivatives	Amino acids, peptides and analogues	Amino acids and derivatives	Amino acids and peptides	H-Tyr-Tyr-Phe-NH2	C27H30N4O5	C1=CC=C(C=C1)CC(C(=O)N)NC(=O)C(CC2=CC=C(C=C2)O)NC(=O)C(CC3=CC=C(C=C3)O)N		Sirius	
332	193,0464	10,56	192,0390		H+	192,12	Organic acids and derivatives	Carboxylic acids and derivatives	Tricarboxylic acids and derivatives		Fatty acids	Citric Acid	C6H8O7	C(C(=O)O)C(CC(=O)O)(C(=O)O)O	Fungus	GNPS	10.1016/0031-9422(96)00146-X
340	203,0513	0,97		180,0713	Na+	180,16	Organic oxygen compounds	Organooxygen compounds	Carbohydrates and carbohydrate conjugated	Monosaccharides	Carbohydrates	Galactose	C6H12O6	OCC1OC(O)C(O)C(O)C1O		Sirius	
342	273,0815	1,07	272,0741		H+		Organic acids and derivatives	Carboxylic acids and derivatives	Amino acids, peptides and analogues	Amino acids and derivatives	Alkaloids		C9H15ClF2N2O3			Sirius	
343	86,0954	1,35	85,0880	63,1154									C5H11N			Sirius	
344	188,0545	3,14		165,0745	Na+	165,14	Organic acids and derivatives	Carboxylic acids and derivatives	Amino acids, peptides and analogues		Amino acids and peptides	2-amino-3,4,5-trihydroxypentanoic acid	C5H11NO5	C(C(C(C(C(=O)O)N)O)O)O		Sirius	
347	197,1276	5,20	196,1202		H+	196,12	Organic acids and derivatives	Carboxylic acids and derivatives	Amino acids, peptides, and analogues	Amino acids and derivatives	Amino acids and peptides	Cyclo(Pro-Val)	C10H16N2O2	C1CC(N(C1)CC(CO)N)CO	Fungus (Aspergillus sp.)	GNPS	10.7164/antibiotics.54.179
348	227,1383	5,69	226,1309		H+	226,27	Organic acids and derivatives	Carboxylic acids and derivatives	Amino acids, peptides, and analogues	Amino acids and derivatives	Amino acids and peptides	Penicillatide A	C11H18N2O3	CC(C)CC(C(=O)N1CCCC1=O)NC=O	Fungus (Penicillium sp.)	Sirius	10.3390/molecules23020394
349	261,1225	5,74	260,1151		H+	260,29	Organic acids and derivatives	Carboxylic acids and derivatives	Amino acids, peptides, and analogues	Amino acids and derivatives	Amino acids and peptides	Cyclo(Pro-Tyr)	C14H16N2O3	C1CC2C(=O)NC(C(=O)N2C1)CC3=CC=C(C=C3)O	Fungus (Aspergillus versicolor)	Sirius	10.3390/md12084326
350	185,1278	5,94	184,1204		H+	184,12	Organic acids and derivatives	Carboxylic acids and derivatives	Amino acids, peptides, and analogues	Amino acids and derivatives	Amino acids and peptides	Cyclo(Ala-Ile)	C9H16N2O2	CCC(C)C1C(=O)NC(C(=O)N1)C	Fungus (Aspergillus sp.)	Npatlas	10.1016/j.jbiotec.2004.07.008
351	192,0648	6,22	191,0574		H+	191,18	Organoheterocyclic compounds	Indoles and derivatives	Indolecarboxylic acids and derivatives		Alkaloids	1-hydroxymethylindole-3-carboxylic acid	C10H9NO3	C1=CC=C2C(=C1)C(=CN2CO)C(=O)O	Bacteria	Sirius	10.1016/j.phytol.2013.06.007
352	211,1432	6,88	210,1358		H+	210,13	Organic acids and derivatives	Carboxylic acids and derivatives	Amino acids, peptides, and analogues	Amino acids and derivatives	Amino acids and peptides	Cyclo(Leu-Pro)	C11H18N2O2	CC(C)CC1C(=O)N2CCCC2C(=O)N1	Bacteria	GNPS	10.1021/np030233e
353	287,0512	12,39		264,0712	Na+	264,233	Phenylpropanoids and polyketides	Coumarins and derivatives			Shikimates and Phenylpropanoids	isocoumarin NM-3	C13H12O6	CC(C1=CC2=CC(=CC(=C2C(=O)O1)O)OC)C(=O)O	Bacteria	Sirius	10.7164/antibiotics.52.426
358	73,5295	0,72	72,5221	50,5495													
359	219,0441	1,06	218,0367		H+		Organic acids and derivatives	Carboxylic acids and derivatives	Amino acids, peptides and analogues	Amino acids and derivatives			C7H10N2O4S			Sirius	
365	174,0517	6,22		151,0717	Na+	151,063	Benzenoids	Benzene and substituted derivatives	Phenylacetamides		Shikimates and phenylpropanoids	P-Hydroxyphenyl-acetamide	C8H9NO2	C1=CC(=CC=C1CC(=O)N)O	Fungus	Sirius	10.1021/np020019a

366	448,1922	7,88	447,1848		H+	447,5	Organoheterocyclic compounds	Indoles and derivatives	Pyrrolindoles	Pyridoindolones	Alkaloids	Oxaline	C24H25N5O4	CC(C)(C=C)C12C=C(C(=O)N3C1(NC(=O)C3=CC4=CN=CN4)N(C5=CC=CC=C25)OC)OC	Fungus (Penicillium sp.)	Sirius	10.1039/C39830000560
367	196,0029	9,37		173,0229	Na+		Organic acids and derivatives	Carboxylic acids and derivatives	Amino acids, peptides and analogues	Amino acids and derivatives	Amino acids and peptides		C6H7NO3S			Sirius	
383	169,0752	5,37	168,0678		H+	168,2	Organoheterocyclic compounds	Indoles and derivatives	Carbolin alkaloids	β-carboline	Alkaloids	β-carboline	C11H8N2	C1=CC=C2C(=C1)C3=C(N2)C=NC=C3	Bacteria	GNPS	10.1271/bbb1961.51.921
384	245,1277	7,46	244,1203		H+	244,29	Organic acids and derivatives	Carboxylic acids and derivatives	Amino acids, peptides, and analogues	Amino acids and derivatives	Amino acids and peptides	Cyclo(Phe-Pro)	C14H16N2O2	C1CC2C(=O)NC(C(=O)N2C1)CC3=CC=CC=C3	Bacteria	GNPS	10.1021/np030233e
385	213,1591	7,71	212,1517		H+	212,15	Organic acids and derivatives	Carboxylic acids and derivatives	Amino acids, peptides, and analogues	Amino acids and derivatives	Amino acids and peptides	Cyclo(Val-Leu)	C11H20N2O2	CC(C)CC1C(=O)NC(C(=O)N1)C(C)C	Fungus (Penicillium sp.)	Npatlas	10.1080/10286020.2013.780349
386	311,1382	7,94	310,1308		H+	310,3478	Organic acids and derivatives	Carboxylic acids and derivatives	Amino acids, peptides and analogues	Amino acids and derivatives	Amino acids and peptides	Cyclo(Tyr-Phe)	C18H18N2O3	C1=CC=C(C=C1)CC2C(=O)NC(C(=O)N2)CC3=CC=C(C=C3)O	Bacteria (Bacillus sp.)	Sirius	10.1007/s10600-009-9270-9
387	247,1433	8,22	246,1359		H+	246,3	Organic acids and derivatives	Carboxylic acids and derivatives	Amino acids, peptides, and analogues	Amino acids and derivatives	Amino acids and peptides	Cyclo(Phe-Val)	C14H18N2O2	CC(C)C1C(=O)NC(C(=O)N1)CC2=CC=CC=C2	Fungus (Aspergillus sp.)	Npatlas	10.1016/j.phytol.2018.01.007
389	170,1897	9,57	169,1823		H+								C11H23N			Sirius	
390	233,1722	10,05	232,1648	210,1922	Na+	210,19	Organic oxygen compounds	Organooxygen compounds	Carbonyl compounds	Alpha beta-unsatured carbonyl compounds	Fatty acids	2-Methyltridec-2-en-4-one	C14H26O	CCCCCCCCC(=O)C=C(C)C	Bacteria	Npatlas	10.1002/cbic.200500174
392	304,2967	10,80	303,2893		H+	303,48	Organic nitrogen compounds	Organonitrogen compounds	Amines	Tertiary amines	Fatty acids	4-[4-[4-(4-aminobutoxy)butylamino]butoxy]butan-1-amine	C16H37N3O2	CCN(CC)CCNCCN(CCOCC)CCOCC		Sirius	
397	355,2799	14,57	354,2725	332,2999													
398	345,1872	14,90	344,1798		H+	344,18	Organic acids and derivatives	Carboxylic acids and derivatives	Tricarboxylic acids and derivatives		Fatty acids	(3S,4S)-3-hydroxytetradec-13-ene-1,3,4-tricarboxylic acid	C17H28O7	C=CCCCCCCCCC(C(=O)O)C(CCC(=O)O)(C(=O)O)O	Fungus (Penicillium sp.)	Npatlas	10.1021/np200528n
399	385,1793	14,90	384,1719		H+	384,1937	Lipid and lipid-like molecules	Fatty acyls	Fatty alcohols	Azaphilones	Terpenoids	9-Decanoyl-3,6a-dimethylfuro[2,3-h]isochromene-6,8-dione	C23H28O5	CCCCCCCCCC(=O)C1=C2C3=COC(=CC3=CC(=O)C2(OC1=O)C)C	Fungus (Aspergillus sp.)	Npatlas	10.1021/acs.jnatprod.5b00436
406	383,1109	0,99	382,1035		H+		Organic oxygen compounds	Organooxygen compounds	Carbohydrates and carbohydrate conjugated	Disaccharides	Carbohydrate		C21H18O7			GNPS	
410	221,0640	1,43		198,0840	Na+								C7H10N4O3			Sirius	
411	322,0300	1,44		299,0500	Na+		Organic oxygen compounds	Organooxygen compounds	Carbohydrates and carbohydrate conjugated		Amino acids and peptides		C7H13N3O8S			Sirius	

412	282,1156	1,69	281,1082		H+	281,2683	Organic acids and derivatives	Carboxylic acids and derivatives	Amino acids, peptides and analogues	Amino acids and derivatives		Pyrizinostatin	C11H15N5O4	CC(=O)CC12N=C(O)N(C)N=C1N(C)C(=O)N(C)C2=O	Bacteria	Npatlas	10.7164/antibiotics.45.1795
418	359,0716	9,50		336,0916	Na+	336,29	Organoheterocyclic compounds	Benzopyrans	1-benzopyrans		Polyketides	Methyl 6,7-dimethoxy-3-(2-methoxy-2-oxoethyl)-4-oxochromene-2-carboxylate	C16H16O8	COC1=C(C=C2C(=C1)C(=O)C=C(O2)C(=O)OC)CC(=O)OC)OC	Fungus	Sirius	10.1016/0021-9673(93)80481-m
420	514,3173	10,15		491,3373	Na+	491,33	Lipid and lipid-like molecules	Steroids and steroid derivatives	Ergostane steroids		Terpenoids	Ergosterimide	C32H45NO3	CC(C)C(C)C=CC(C)C1CC2C3C(C4C=C5CC(CCC5(C6C4=C2C1(CC6)C)C)O)C(=O)NC3=O	Fungus (Aspergillus sp.)	Npatlas	10.1016/j.steroids.2007.05.009
423	441,3673	18,47	440,3599	418,3873			Lipid and lipid-like molecules	Fatty acyls	Glycerolipids		Fatty acids					Sirius	
424	90,5048	19,42	89,4974	67,5248													
435	300,1665	8,79	299,1591		H+	299,15	Organoheterocyclic compounds	Indoles and derivatives	Carbobazoles	Amino acids and derivatives	Alkaloids	Streptoverticillin	C18H21NO3	CC1=C(C2=C(C3=CC=CC=C3N2)C(=C1OC)OC)C[C@@H](C)O	Bacteria	Sirius	10.1038/ja.2007.19
436	350,1453	9,48		327,1653	Na+	327,4	Organic acids and derivatives	Carboxylic acids and derivatives	Amino acids, peptides and analogues		Amino acids and peptides	Brevianamide X	C18H21N3O3	COCN1C=C(C[C@@H]2NC(=O)[C@@H]3CCCN3C2=O)C2=CC=CC=C21	Fungus (Aspergillus sp.)	Npatlas	10.3389/fmicb.2017.01284
437	495,1968	10,49		472,2168	Na+	472,5	Organic acids and derivatives	Carboxylic acids and derivatives	Amino acids, peptides and analogues	Amino acids and derivatives	Amino acids and peptides	N-[1-[[2-[(3-amino-3-oxo-1-phenylpropyl)amino]-2-oxoethyl]amino]-1-oxo-3-phenylpropan-2-yl]benzamide	C27H28N4O4	C1=CC=C(C=C1)CC(C(=O)NCC(=O)NC(CC(=O)N)C2=CC=CC=C2)NC(=O)C3=CC=CC=C3		Sirius	
439	287,0510	11,82		264,0710	Na+	264,23	Organoheterocyclic compounds	Benzopyrans	2-benzopyrans		Shikimates and Phenylpropanoids	6-hydroxy-8-methoxy-3a-methyl-3a,9b-dihydro-3h-furo[3,2-c]isochromene-2,5-dione	C13H12O6	CC12CC(=O)OC1C3=C(C(=CC(=C3)OC)O)C(=O)O2	Fungus	Sirius	10.1016/j.fitote.2011.10.013
441	639,1646	13,24	638,1572		H+	638,6	Organoheterocyclic compounds	Benzopyrans	1-benzopyrans	Xanthenes	Polyketides	Secalonic acid F	C32H30O14	CC1CC(=O)C2=C(C3=C(C=CC(=C3O)C4=C(C5=C(C=C4)OC6(C(C(CC(=O)C6=C5O)C)O)C(=O)OC)O)OC2(C1O)C(=O)OC)O	Fungus	Sirius	10.1021/np9003728
442	330,2594	13,71	329,2520		H+		Organic acids and derivatives	Carboxylic acids and derivatives	Amino acids, peptides and analogues	Amino acids and derivatives	Alkaloids		C19H31BN2O2			Sirius	

443	200,1978	13,75	199,1904		H+	199,33	Lipid and lipid-like molecules	Fatty acyls	Fatty amides		Fatty acids	Dodecanamide	C12H25NO	CCCCCCCCCCCC(=O)N	Eukaryota	GNPS	10.1016/S0031-9422(00)00183-7
447	514,2641	17,70		491,2841	Na+	491,6	Benzenoids	Benzene and substituted derivatives	Benzoyl derivatives	1-benzoylpiperidines	Alkaloids	8-[4-[4-(2-Oxo-3,4-dihydroquinolin-1-yl)piperidine-1-carbonyl]phenoxy]octanamide	C29H37N3O4	C1CN(CCC1N2C(=O)CCC3=CC=CC=C32)C(=O)C4=CC=C(C=C4)OCCCCCCCC(=O)N		Sirius	
452	88,0022	19,38	86,9948	65,0222									C2HNO3			Sirius	
455	211,1405	7,09	210,1331		H+	210,13	Organic acids and derivatives	Carboxylic acids and derivatives	Amino acids, peptides, and analogues	Amino acids and derivatives	Amino acids and peptides	Cyclo(Leu-Pro)	C11H18N2O2	CC(C)CC1C(=O)N2CCCC2C(=O)N1	Bacteria	GNPS	10.1021/np030233e
463	164,0677	6,72	163,0603		H+	163,17	Benzenoids	Benzene and substituted derivatives	Styrenes		Shikimates and phenylpropanoids	N-(4-hydroxystyryl) formamide	C9H9NO2	C1=CC(=CC=C1/C=C\NC=O)O	Fungus (Aspergillus sp.)	Sirius	10.7164/antibiotics.37.469
464	284,1350	7,98	283,1276		H+	283,32	Organic acids and derivatives	Carboxylic acids and derivatives	Amino acids, peptides and analogues		Alkaloids	Brevianamide F (Trp-Pro)	C16H17N3O2	C1CC2C(=O)NC(C(=O)N2C1)CC3=CNC4=CC=CC=C43	Fungus (Aspergillus versicolor)	GNPS	10.1002/anie.200800106
466	394,1708	8,98	393,1634	371,1908													
475	394,1702	11,64		371,1902	Na+	371,39	Nucleosides, nucleotides, and analogues	Pyrimidine nucleosides				(2S)-2,6-diamino-N-[1-[(2R,3R,4S,5R)-3,4-dihydroxy-5-(hydroxymethyl)oxolan-2-yl]-2-oxopyrimidin-4-yl]hexanamide	C15H25N5O6	C1=CN(C(=O)N=C1NC(=O)C(CCCCN)N)C2C(C(C(O2)CO)O)O		Sirius	
477	268,2228	13,53		245,2428	Na+		Organic nitrogen compounds	Organonitrogen compounds	Amines	Secondary amines	Fatty acids		C14H31NO2			Sirius	
478	293,1704	13,86	292,1630		H+	292,33	Alkaloids and derivatives	Ergoline and derivatives	Lysergic acids and derivatives		Amino acids and peptides	5-methoxy-2-[2-(2-methoxyethoxy)ethylcarbamoylamino]pentanoic acid	C12H24N2O6	COCCCC(C(=O)O)NC(=O)NCCOCCOC		Sirius	
479	228,1004	14,20		205,1204	Na+		Benzenoids	Benzene and substituted derivatives	Nitrobenzenes		Alkaloids		C12H15NO2			Sirius	
480	675,6666	16,51			2M+H	337,6	Lipid and lipid-like molecules	Fatty acyls	Fatty amides		Fatty acids	13-Docosenamide	C22H43NO	CCCCCCCCC=CCCCCCCCCCCCC(=O)N	Bacteria	GNPS	10.3389/fmicb.2017.02704/full
483	343,2903	11,22	342,2829	320,3103	Na+		Lipid and lipid-like molecules	Prenol lipids			Terpenoids		C20H38BO2			Sirius	
484	394,3992	18,21	393,3918	371,4192			Lipid and lipid-like molecules	Fatty acyls	Fatty amides		Fatty acids					Sirius	
486	191,0780	6,47	190,0706		H+	190,07	Organoheterocyclic compounds	Diazanaphthalenes	Benzodiazines	Quinazolines	Alkaloids	2-(1-hydroxyethyl)-4(3H)quinazoline	C8H12N2O2	CC(C1=NC2=CC=CC=C2C(=O)N1)O	Fungus	Sirius	10.1139/v93-176
491	72,0422	1,10	71,0348	49,0622													
492	100,0371	1,10	99,0297		H+		Organic nitrogen compounds	Organonitrogen compounds	Imines		Alkaloids		C4H5NO2			Sirius	

494	433,1013	1,38	432,0939		H+	432,1056	Phenylpropanoids and polyketides	Isoflavonoids	Isoflavonoid O-glycosides		Shikimates and phenylpropanoids	5,7-dihydroxy-3-{4-hydroxy-3-[(3,4,5-trihydroxy-6-methyloxan-2-yl)oxy]phenyl}chromen-4-one	C21H20O10	CC1C(C(C(C(O1)OC2=C(C=CC(=C2)C3=COC4=CC(=CC(=C4C3=O)O)O)O)O)O)O	Bacteria	Sirius	10.1038/ja.2005.100
496	461,1317	1,61	460,1243		H+	460,13	Phenylpropanoids and polyketides	Isoflavonoids	Isoflavonoid O-glycosides		Shikimates and phenylpropanoids	7-[3,4-Dihydroxy-6-(hydroxymethyl)-5-methoxyoxan-2-yl]oxy-3-(4-hydroxyphenyl)-6-methoxychromen-4-one	C23H24O10	COC1C(OC(C(C1O)O)OC2=C(C=C3C(=C2)OC=C(C3=O)C4=CC=C(C=C4)O)OC)CO	Fungus	Sirius	10.1016/j.foodchem.2009.02.055
499	301,0663	8,35		278,0863	Na+	278,26	Phenylpropanoids and polyketides	Coumarins and derivatives			Polyketides	2-(5,7-dimethoxy-4-methyl-2-oxochromen-3-yl)acetic acid	C14H14O6	CC1=C(C(=O)OC2=C1C(=CC(=C2)OC)OC)CC(=O)O		Sirius	
509	250,1396	7,78		227,1596	Na+		Lipid and lipid-like molecules	Fatty acyls	Fatty amides				C12H21NO3			Sirius	
510	401,2634	10,30		378,2834	Na+	378,5463	Lipid and lipid-like molecules	Steroids and steroid derivatives	Bile acids, alcohols and derivatives	Hydroxy bile acids, alcohols and derivatives	Terpenoids	3-hydroxy-3-(3-hydroxy-10,13-dimethyl-2,3,4,5,6,7,8,9,11,12,14,15,16,17-tetradecahydro-1H-cyclopenta[a]phenanthren-17-yl)butanoic acid	C23H38O4	CC(O)(CC(=O)O)C1CCC2C3CCC4CC(O)CCC4(C)C3CCC21C	Eukaryota	Sirius	10.1016/0031-9422(89)85019-8
511	292,1501	10,89		269,1701	Na+		Organic acids and derivatives	Carboxylic acids and derivatives	Amino acids, peptides and analogues	Amino acids and derivatives			C14H23NO4			Sirius	
516	487,2638	12,97	486,2564		H+	486,6	Lipid and lipid-like molecules	Steroids and steroid derivatives	Steroid esters		Terpenoids	Andrastin A	C28H38O7	CC1C(=O)C2(C(=CC3C(C2(C1=O)C(=O)OC)(CCC4C3(CCC(C4(C)C)OC(=O)C)C=O)C)C)C	Fungus (Penicillium sp.)	GNPS	
519	342,1972	7,28	341,1898		H+	341,17	Organic acids and derivatives	Carboxylic acids and derivatives	Amino acids, peptides, and analogues		Amino acids and peptides	14-hydroxyterezine D	C19H23N3O3	CC1C(=O)N(C(C(=O)N1)CC2=CNC3=C(C=CC=C23)CC=C(C)C)O	Fungus (Aspergillus sp.)	Npatlas	10.1021/np700737g
526	309,1210	7,89		286,1410	Na+	286,33	Organic acids and derivatives	Carboxylic acids and derivatives	Amino acids, peptides and analogues	Amino acids and derivatives	Alkaloids	(3Z,6E)-1-N-methyl-3-benzylidene-6-(2R-methyl-3-hydroxypropylidene)piperazine-2,5-dione	C14H22O6	CC(CO)C=C1C(=O)NC(=CC2=CC=CC=C2)C(=O)N1C	Bacteria	Npatlas	10.3390/md11041035
529	343,2681	18,33	342,2607	320,2881													
531	396,4147	18,59	395,4073	373,4347			Lipid and lipid-like molecules	Fatty acyls	Fatty amides		Fatty acids					Sirius	
536	597,4769	18,02	596,4695	574,4969													
538	526,4771	19,05		503,4971	Na+		Lipid and lipid-like molecules				Alkaloids		C36H61B			Sirius	

544	682,2422	11,56	681,2348		H+	681,6	Organic acids and derivatives	Carboxylic acids and derivatives	Amino acids, peptides and analogues		Amino acids and peptides	2-(3-((S)-5-((S)-1-amino-3-(3-hydroxy-4-nitrophenyl)-1-oxopropan-2-ylamino)-4-(3,5-dimethoxybenzamido)-5-oxopentylcarbamoyl)phenoxy)acetic acid	C32H35N5O12	COC1=CC(=CC(=C1)C(=O)NC(CCCNC(=O)C2=CC(=CC=C2)OCC(=O)O)C(=O)NC(CC3=CC(=C(C=C3)[N+](=O)[O-])O)C(=O)N)OC		Sirius	
547	71,0267	0,74	70,0193	48,0467									C2H5F			Sirius	
548	79,0189	1,10	78,0115	56,0389									C5H2O			Sirius	
558	323,1002	7,99		300,1202	Na+	300,111	Organic acids and derivatives	Carboxylic acids and derivatives	Amino acids, peptides and analogues	Amino acids and derivatives	Alkaloids	Vertilecanin B Methy ester	C16H16N2O4	COC(=O)CNC(=O)C1=NC=C(C=C1)C(C2=CC=CC=C2)O	Fungus	Sirius	10.1021/np000094q
559	305,1336	8,75		282,1536	Na+	282,158	Organoheterocyclic compounds	Pyranones and derivatives	Pyrrolines		Polyketides	Cladosin A	C14H22N2O4	CC(CC(CC(=N)C1=C(C(=C(C)C)NC1=O)O)OC)O	Fungus	Sirius	10.1021/np400833x
560	323,1443	8,76	322,1369		H+		Organic acids and derivatives	Carboxylic acids and derivatives	Amino acids, peptides and analogues	Amino acids and derivatives			C19H18N2O3			Sirius	
561	321,1297	10,72	320,1223		H+	320,1195	Organoheterocyclic compounds	Pyrroles	Substituted pyrroles		Amino acids and peptides	Reductiline	C16H20N2O3S	CSCCCN1C=CC(=C1)/C=C/C(=O)NC2=C(CCC2=O)O	Bacteria	Natlas	10.1016/s0040-4039(00)85765-2
563	235,1655	11,71		212,1855	Na+		Lipid and lipid-like molecules	Fatty acyls	Fatty acyls		Fatty acids		C13H24O2			Sirius	
564	501,2425	12,02	500,2351		H+	500,24	Lipid and lipid-like molecules	Steroids and steroid derivatives	Steroids esters		Terpenoids	Andrastone C	C28H36O8	CC1=CC2C3(C(C(C4C2(CCC(C4(C)C)OC(=O)C)C=O)O)OC5=C(C(=O)C1(C53C(=O)OC)C)C)C	Fungus (Penicillium sp.)	GNPS	10.3390/md18050258
565	359,1787	12,80	358,1713	336,1987			Lipid and lipid-like molecules	Steroids and steroid derivatives	Cholane steroids		Terpenoids					Sirius	
567	340,3526	16,44	339,3452	317,3726			Organic nitrogen compounds	Organonitrogen compounds	Guanidines		Alkaloids		C20H43N4			Sirius	
568	405,3209	16,62	404,3135	382,3409													
572	340,3526	18,10	339,3452	317,3726			Organic nitrogen compounds	Organonitrogen compounds	Guanidines		Alkaloids		C20H43N4			Sirius	
578	446,2017	10,69	445,1943		H+	445,5	Organoheterocyclic compounds	Benzopyrans	2-benzopyrans		Alkaloids	(1S,19S)-9,9,16,16-tetramethyl-14-oxido-8-oxa-23,25-diaza-14-azoniaheptacyclohexacosa-2,4(13),5,7(12),10,14-hexaene-24,26-dione	C26H27N3O4	CC1(C=CC2=C(O1)C=CC3=C2[N+](=C4C3=CC56C(C4(C)C)CC7(CCCN7C5=O)C(=O)N6)[O-])C	Fungus (Aspergillus sp.)	GNPS	

579	432,2220	11,48	431,2146		H+	431,5	Organoheterocyclic compounds	Quinolines and derivatives	Pyrroloquinolines		Alkaloids	Stephacidin A	C26H29N3O3	CC1(C=CC2=C(O1)C=CC3=C2NC4=C3CC56C(C4(C)C)CC7(CCCN7C5=O)C(=O)N6)C	Fungus (Aspergillus sp.)	GNPS	
584	282,0830	1,06		259,1030	Na+	259.22	Organic oxygen compounds	Organooxygen compounds	Carbohydrates and carbohydrate conjugated	Glycosyl compounds	Carbohydrates	4,6-Diamino-1-[3,4-dihydroxy-5-(hydroxymethyl)oxolan-2-yl]-1,3,5-triazin-2-one	C8H13N5O5	C(C1C(C(C(O1)N2C(=NC(=NC2=O)N)N)O)O)O		Sirius	
585	180,0987	6,23	179,0913		H+	179,0946	Organoheterocyclic compounds	Pyridines and derivatives	Pyridine alkaloids	Pyridinecarboxylic acids	Alkaloids	Fusaric acid	C10H13NO2	CCCCC1=CN=C(C(=O)O)C=C1	Fungus	Sirius	10.1080/14786419.2017.1415897
588	332,1702	10,71	331,1628	309,1902													
591	205,1916	11,63		182,2116	Na+		Lipid and lipid-like molecules	Fatty acyls	Fatty acyls		Fatty acids		C13H26			Sirius	
592	374,2275	15,03	373,2201	351,2475													
597	268,1002	1,36	267,0928		H+	267,24	Nucleosides, nucleotides, and analogues	Purine nucleoside			Carbohydrates	Xylosyladenine	C10H13N5O4	C1=NC(=C2C(=N1)N(C=N2)C3C(C(C(O3)CO)O)O)N	Fungus	Sirius	10.1021/np100902f
605	295,1070	5,57	294,0996	272,1270									C15H16N2O3			Sirius	
607	610,2437	7,46	609,2363		H+								C33H47N5O6			Sirius	
608	433,1083	8,53	432,1009		H+	432,1056	Organoheterocyclic compounds	Azaphilones	Chromanes		pokyketides	Purpurquinone B	C21H20O10	CC=CC1=CC2=CC(=O)C(C(=O)C2(C(O1)O)O)(C)OC(=O)C3=C(C(=C(C=C3O)O)O)C	Fungus (Penicillium sp.)	Npatlas	10.1021/np2004769
610	464,2955	10,18		441,3155	Na+		Lipid and lipid-like molecules	Steroids and steroid derivatives			Terpenoids		C24H43NO6			Sirius	
612	236,0276	11,38		213,0476	Na+	213.3	Organoheterocyclic compounds	Azoles	Thiazoles			N-(2-Thiazolyl)pyrrolidine-1-thiacarboxamide	C8H11N3S2	C1CCN(C1)C(=S)NC2=NC=CS2		Sirius	
615	317,2068	14,41		294,2268	Na+	294,43	Lipid and lipid-like molecules	Fatty acyls	Lineolic acids and derivatives		Fatty acids	11-Keto-9(E),12(E)-octadecadienoic acid	C18H30O3	CCCCC/C=C/C(=O)/C=C/CCCCCCCC(=O)O	Fungus	Sirius	10.7164/antibiotics.52.171
616	300,9886	15,13		278,0086	Na+	278,36	Organosulfur compounds	Thioethers	Aryl thioethers		Pseudoalkaloids	N''-acetyl-2-[(5-methylsulfanyl-1,3,4-thiadiazol-2-yl)sulfanyl]acetohydrazide	C7H10N4O2S3	CC(=O)NNC(=O)CSC1=NN=C(S1)SC		Sirius	
617	755,0342	17,21	754,0268	732,0542													
618	219,0468	1,09	218,0394		H+		Organoheterocyclic compounds	Azoles	Triazoles		Alkaloids		C6H11N4OPS			Sirius	
619	100,0387	1,10	99,0313		H+		Organic nitrogen compounds	Organonitrogen compounds	Imines		Alkaloids		C4H5NO2			Sirius	
625	282,1171	1,59	281,1097		H+	281,2683	Nucleosides, nucleotides, and analogues	Purine nucleoside			Carbohydrates	2-O-Methyladenosine	C11H15N5O4	COC1C(C(OC1N2C=NC3=C(N=CN=C32)N)CO)O	Fungus	GNPS	10.1021/np100902f

630	100,0748	2,56	99,0674		H+		Organoheterocyclic compounds				Alkaloids	Azinanone (piperin-4-one)	C5H9NO			Sirius	
638	263,0996	6,01		240,1196	Na+	240,09	Organoheterocyclic compounds	Benzopyrans	2-benzopyrans		Polyketides	3S,4R-3,8-dimethoxy-3-methylisochromane-4,6-diol	C12H16O5	CC1(C(C2=C(CO1)C(=CC(=C2)O)OC)O)OC	Fungus (Aspergillus sp.)	Sirius	10.3390/molecules23071709
639	277,1144	7,22	276,1070		H+	276,099	Benzenoids	Benzene and substituted derivatives	Diphenylmethanes		Shikimates and phenylpropanoids	Terrestrol H	C15H16O5	COCC1=C(C=C(C(=C1)O)CC2=C(C=CC(=C2)O)O)O	Fungus (Penicillium sp.)	Npatlas	10.1021/np070421v
641	233,0779	8,96		210,0979	Na+	210,23	Organic oxygen compounds	Organooxygen compounds	Carboxyl compounds	Ketones	Polyketides	O-Methylxanthoxylin	C11H14O4	CC(=O)C1=C(C=C(C=C1OC)OC)OC		Sirius	
647	279,1554	14,21	278,1480		H+	278,34	Benzenoids	Benzene and substituted derivatives	Benzoic acids and derivatives	Benzoic acid esters	Shikimates and phenylpropanoids	Dibutyl Phthalate	C16H22O4	CCCCOC(=O)C1=CC=CC=C1C(=O)OCCCC	Eukaryota	GNPS	
649	366,3684	17,01	365,3610	343,3884			Lipid and lipid-like molecules	Fatty acyls	Fatty amides		Fatty acids					Sirius	
652	61,0050	0,73	59,9976		H+								C5			Sirius	
653	70,0102	0,73	69,0028	47,0302									F2HNO			Sirius	
655	72,0784	1,03	71,0710	49,0984													
688	298,0928	3,53	297,0854		H+	297,34	Nucleosides, nucleotides, and analogues	5'-deoxyribonucleosides	5'-deoxy-5'-thionucleosides		Carbohydrates	5'-Deoxy-5'-methylthioadenosine	C11H15N5O3S	CSCC1C(C(C(O1)N2C=NC3=C(N=CN=C32)N)O)O	Bacteria	GNPS	10.1111/1462-2920.12899
694	338,2796	18,19	337,2722	315,2996	Na+		Benzenoids	Benzene and substituted derivatives			Alkaloids		C22H37N			Sirius	
695	465,3779	18,93	464,3705	442,3979													
703	170,1874	9,58	169,1800		H+	169,31	Organic nitrogen compounds	Organonitrogen compounds	Cyclohexylamines		Terpenoids		C11H23N	CCNC1CCCC1C(C)C		Sirius	
704	572,3372	10,42	571,3298		H+	571,79	Organoheterocyclic compounds	Naphthopyrans	Diterpenoids		Alkaloids	21,22-Diprenylpaxilline	C37H49NO4	CC(=CCC1=CC2=C(C=C1CC=C(C)C)NC3=C2CC4C3(C5(CCC6C(=CC(=O)C(O6)C(C)(C)O)C5(CC4)O)C)C)C	Fungus (Penicillium sp.)	Npatlas	10.1021/np400304q
705	309,0820	10,47		286,1020	Na+		Organic acids and derivatives	Carboxylic acids and derivatives	Amino acids, peptides and analogues	Amino acids and derivatives	Amino acids and peptides		C10H14N4O6			Sirius	
706	352,1971	10,72	351,1897		H+	351,4	Organic acids and derivatives	Carboxylic acids and derivatives	Amino acids, peptides and analogues	Amino acids and derivatives	Amino acids and peptides	Trypostatin B	C21H25N3O2	CC(=CCC1=C(C2=CC=CC=C2N1)CC3C(=O)N4CCCC4C(=O)N3)C	Fungus (Aspergillus sp.)	GNPS	10.7164/antibiotics.49.534
710	355,2774	15,02	354,2700		H+		Lipid and lipid-like molecules	Fatty acyls	Fatty acyls		Fatty acids		C19H30O6			Sirius	
712	300,1038	1,03		277,1238	Na+		Organic acids and derivatives	Carboxylic acids and derivatives	Amino acids, peptides and analogues	Amino acids and derivatives	Amino acids and peptides		C12H14BFN3O3			Sirius	

713	355,2799	14,48		332,2999	Na+	332,4	Lipids and lipid-like molecules	Prenol lipids	Diterpenoids	Gibberellins	Terpenoids	Gibberellin A12	C20H28O4	CC12CCCC(C1C(C34C2CCC(C3)C(=C)C4)C(=O)O)(C)C(=O)O	Fungus	GNPS	10.1016/j.phytochem.2007.08.026
715	317,2067	12,33		294,2267	Na+	294,43	Lipid and lipid-like molecules	Fatty acyls	Lineolic acids and derivatives		Fatty acids	11-Keto-9(E),12(E)-octadecadienoic acid	C18H30O3	CCCCC/C=C/C(=O)/C=C/CCCCCCCC(=O)O	Fungus	Sirius	10.7164/antibiotics.52.171
716	317,2068	13,05		294,2268	Na+	294,43	Lipid and lipid-like molecules	Fatty acyls	Lineolic acids and derivatives		Fatty acids	11-Keto-9(E),12(E)-octadecadienoic acid	C18H30O3	CCCCC/C=C/C(=O)/C=C/CCCCCCCC(=O)O	Fungus	Sirius	10.7164/antibiotics.52.171
717	215,0127	1,48		192,0327	Na+	214,3	Organic nitrogen compounds	Organonitrogen compounds	N-arylamides	N-acetylarylamines	Alkaloids	Holomycin	C7H6N2O2S2	CC(=O)NC1=C2C(=CSS2)NC1=O	Bacteria	Sirius	10.7164/antibiotics.30.334
718	79,0191	1,50	78,0117	56,0391									C5H2O			Sirius	
726	352,3521	17,44	351,3447		H+		Lipid and lipid-like molecules	Fatty acyls	Fatty amides		N-acyl amines		C21H43BNO2			Sirius	
729	225,0834	1,42		202,1034	Na+	202,21	Organic acids and derivatives	Carboxylic acids and derivatives	Amino acids, peptides and analogues		Amino acids and peptides	3,6-bis(1-hydroxyethyl)piperazine-2,5-dione	C8H14N2O4	CC(C1C(=O)NC(C(=O)N1)C(C)O)O		Sirius	
741	375,0989	5,69		352,1189	Na+	352,115	Organoheterocyclic compounds	Naphthopyrans	Naphthopyranones		Polyketides	O-ethylhydroxydihydrofusarubin	C17H20O8	CCOC1(CC2C(=O)C3=C(C(=CC(=C3O)OC)O)C(=O)C2(CO1)O)C	Fungus	Sirius	10.7164/antibiotics.32.685
744	357,0886	6,59		334,1086	Na+	334,105	Benzenoids	Anthraquinones and anthrones	Anthraquinones	Hydroxyanthraquinones	Polyketides	Aspetritone B	C17H18O7	CC1(CC2=C(CC1O)C(=O)C3=CC(=C(C(=C3C2=O)O)OC)OC)O	Fungus (Aspergillus sp.)	Sirius	10.3390/md15110348
749	267,1552	7,83		244,1752	Na+	244,33	Organic acids and derivatives	Tetrahydrofurans			Polyketides	(2S)-2-[(2R,5S)-5-[(2S,3S)-3-hydroxyhexan-2-yl]oxolan-2-yl]propanoic Acid	C13H24O4	CCCC(C(C)C1CCC(O1)C(C)C(=O)O)O		Sirius	
754	158,1515	10,77		135,1715	Na+	135,21	Organic acids and derivatives	Carboxylic acids and derivatives	Amino acids, peptides and analogues	Amino acids and derivatives	Alkaloids	N-methyl-1-phenylethylamine	C9H13N	CNC(C)C1=CC=CC=C1		Sirius	
758	329,2101	12,81		306,2301	Na+		Lipid and lipid-like molecules	Fatty acyls	Fatty acids and conjugated		Fatty acids		C20H34O2			Sirius	
775	367,2569	15,89	366,2495	344,2769													
777	380,3478	16,34		357,3678	Na+	357,6	Organic nitrogen compounds	Organonitrogen compounds	Amines	Alkanolamines	Fatty amides	20-(2-Hydroxyethylamino)icosan-1-ol	C22H47NO2	C(CCCCCCCCCCO)CCCCCCCCCNCCO		Sirius	
779	421,3031	16,55	420,2957	398,3231													
781	410,3952	17,78	409,3878	387,4152													
783	376,3173	18,52	375,3099		H+		Lipid and lipid-like molecules	Fatty acyls	Fatty amides		Fatty acids		C24H41NO2			Sirius	
785	298,3090	18,76	297,3016		H+	297,4	Lipid and lipid-like molecules	Fatty acyls	Fatty amides		Fatty acids	Nonadecanamide	C19H39NO	CCCCCCCCCCCCCCCCCCC(=O)N		Sirius	
786	364,3534	19,02		341,3734	Na+	341,6	Organic nitrogen compounds	Organonitrogen compounds	N-organohydroxylamines		N-acyl amines	N-Hydroxydocosan-1-amine	C22H47NO	CCCCCCCCCCCCCCCCCCCCCCNO		Sirius	
788	53,0162	0,72	52,0088		H+								CH5Cl			Sirius	

794	223,0599	10,99	222,0525		H+		Organic acids and derivatives	Carboxylic acids and derivatives	Dicarboxylic acids and derivatives		Shikimates		C8H11FO6			Sirius	
795	204,1345	11,10	203,1271		H+	203,24	Organoheterocyclic compounds	Imidazopyrimidines	Purines and purines derivatives	6-aminopurines	Alkaloids	6-isopentenyladenine	C10H13N5	CC(=CCNC1=NC=NC2=C1NC=N2)C	Bacteria (Pseudomonas sp.)	Sirius	10.1016/S0031-9422(00)98049-X
798	395,2716	14,51	394,2642	372,2916													
800	591,4566	18,41	590,4492		H+		Lipid and lipid-like molecules	Fatty acyls	Fatty amides				C30H60N6O4			Sirius	
801	513,4058	18,47	512,3984	490,4258													
802	566,4918	18,92	565,4844		H+		Organic acids and derivatives	Carboxylic acids and derivatives	Amino acids, peptides and analogues	Amino acids and derivatives	Fatty acids		C33H63N3O4			Sirius	
808	511,2855	8,89	510,2781		H+	510,284	Organic acids and derivatives	Carboxylic acids and derivatives	Amino acids, peptides and analogues		Amino acids and peptides	Belaid A	C28H38N4O5	CC(C)C(C(=O)NC(C(C)C)C(=O)NC(CC1=CC=CC=C1)C(=O)O)NC(=O)C(CC2=CC=CC=C2)N	Fungus (Penicillium sp.)	Npatlas	10.1073/pnas.1908662116
809	379,1700	9,56	378,1626	356,1900													
812	224,0884	10,89	223,0810		H+	223,22	Organic acids and derivatives	Carboxylic acids and derivatives	Amino acids, peptides and analogues	Amino acids and derivatives	Shikimates and phenylpropanoids		C11H13NO4			Sirius	
813	246,1454	10,89		223,1654	Na+	223.31	Lipid and lipid-like molecules	Fatty acyls	Fatty amides	N-acyl amines	Alkaloids	2-cyclopent-2-en-1-yl-N-[(2-hydroxycyclopentyl)methyl]acetamide	C13H21NO2	C1CC(C(C1)O)CNC(=O)CC2CCC=C2		Sirius	
815	315,1915	12,69	314,1841		H+	314,5	Benzenoids	Phenols	Benzenediols	Resorcinols	Polyketides	Cardoltriene	C21H30O2	C=CCC=CCC=CCCCCCCCC1=CC(=CC(=C1)O)O	Eukaryota	GNPS	
822	208,0931	7,57	207,0857		H+	207,23	Organic acids and derivatives	Carboxylic acids and derivatives	Amino acids, peptides and analogues	Amino acids and derivatives	Amino acids and peptides	N-Acetyl-phenylalanine	C11H13NO3	CC(=O)NC(CCCC=C)C(=O)O	Fungus	GNPS	
825	350,1604	9,56	349,1530	327,1804													
828	342,3686	10,80	341,3612		H+		Organic nitrogen compounds	Organonitrogen compounds	Cyclohexylamines		Alkaloids		C20H45N4			Sirius	
829	298,3428	11,25	297,3354	275,3628													
831	229,9970	11,62	228,9896		H+		Organic nitrogen compounds	Organonitrogen compounds	Amines		Shikimates and phenylpropanoids		C9H9BrFN			Sirius	
832	227,9991	11,62		205,0191	Na+	206,0800	Organoheterocyclic compounds	Azolidines	Isoxazolines		Shikimates and phenylpropanoids	4-bromo-2-ethyl-3,5-dimethyl-3H-1,2-oxazole	C7H12BrNO	CCN1C(C(=C(O1)C)Br)C		Sirius	
833	326,3741	12,02	325,3667	303,3941													
834	197,1507	12,15		174,1707	Na+	174,28	Lipids and lipid-like molecules	Fatty acyls	Fatty alcohols		Fatty acids	2,6-dimethyloctane-1,8-diol	C10H22O2	CC(CO)CCCC(C)CCO	Eukaryota	Sirius	10.1007/s10600-010-9598-1

835	211,1661	12,39	210,1587		H+		Organoheterocyclic compounds	Azoles			Alkaloids		C11H20N3O			Sirius	
836	226,1188	12,55		203,1388	Na+	225,11	Organoheterocyclic compounds	Pyridines and derivatives			Alkaloids	4-phenyl-3-(pyridine-2-yl)but-2-en-1-ol	C15H15NO	C1=CC=C(C=C1)C/C(=C\CO)/C2=CC=CC=N2	Bacteria	Sirius	10.1016/j.bmcl.2006.08.015
837	913,5067	12,61	912,4993	890,5267		913,1	Lipid and lipid-like molecules	Prenol lipids	Terpene glycosides	Triterpene glycosides	Terpenoids	(2S,3S,4S,5R,6R)-6-[[(3S,6aR,6bS,8aR,9R,14bS)-9-hydroxy-14b-(hydroxymethyl)-4,4,6a,6b,8a,11,11-heptamethyl-1,2,3,4a,5,6,7,8,9,10,12,12a,14,14a-tetradecahydropicen-3-yl]oxy]-5-[(2S,3R,4S,5R)-4,5-dihydroxy-3-[(2S,3R,4R,5R,6S)-3,4,5-trihydroxy-6-methyloxan-2-yl]oxyoxan-2-yl]oxy-3,4-dihydroxyoxane-2-carboxylic acid	C47H79O17	CC1C(C(C(C(O1)OC2C(C(COC2OC3C(C(C(OC3OC4CCC5(C(C4(C)C)CCC6(C5CC=C7C6(CCC8(C7CC(CC8O)(C)C)C)C)CO)C(=O)O)O)O)O)O)O)O)O		Sirius	
844	468,3846	14,28	467,3772		H+		Lipid and lipid-like molecules	Fatty acyls	Fatty acids		Fatty acids		C27H49NO5			Sirius	
845	319,2802	14,52		296,3002	Na+		Lipid and lipid-like molecules	Fatty acyls	Fatty acids		Fatty acids		C19H36O2			Sirius	
847	341,2623	14,52	340,2549	318,2823													
848	228,2282	15,38	227,2208		H+	227,39	Lipid and lipid-like molecules	Fatty acyls	Fatty amides		Fatty acids	Tetradecanamide	C14H29NO	CCCCCCCCCCCCCC(=O)N	Eukaryota	GNPS	
849	275,2538	15,43		252,2738	Na+		Lipid and lipid-like molecules	Fatty acyls	Fatty acyls		Fatty acids		C18H36			Sirius	
850	297,2360	15,44		274,2560	Na+		Organic acids and derivatives	Carboxylic acids and derivatives	Carboxylic acid derivatives	Carboxylic acid amides	Alkaloids		C12H30N6O			Sirius	
851	361,3263	17,03	360,3189		H+		Lipid and lipid-like molecules	Fatty acyls	Fatty acyls		Polyketides		C24H40O2			Sirius	
852	383,3089	17,02	382,3015		H+								C22H38O5			Sirius	
853	485,3750	17,11	484,3676	462,3950													
854	419,3676	17,40	418,3602		H+		Lipid and lipid-like molecules	Fatty acyls	Fatty acyls		Fatty acyls		C31H46			Sirius	
855	436,3940	17,40	435,3866		H+		Lipid and lipid-like molecules	Steroids and steroid derivatives			Terpenoids		C26H49N3O2			Sirius	
856	441,3493	17,40		418,3693	Na+		Lipid and lipid-like molecules	Fatty acyls	Fatty acyls		Fatty acyls		C31H46			Sirius	
858	494,4355	17,78	493,4281		H+		Lipid and lipid-like molecules	Steroids and steroid derivatives	Azasteroids and derivatives		Terpenoids		C29H55N3O3			Sirius	
859	499,3909	17,78	498,3835	476,4109													
860	531,4013	17,89	530,3939	508,4213													
861	596,5026	17,89		573,5226	Na+		Lipid and lipid-like molecules	Fatty acyls	Sphingolipids		Fatty acids		C37H67NO3			Sirius	

863	359,2897	17,96	358,2823		H+	358,5	Lipid and lipid-like molecules	Fatty acyls	Fatty amides		Fatty acids	N-[2-[2-Carboxyethyl(2-hydroxyethyl)amino]ethyl]dodecanamide	C19H38N2O4	CCCCCCCCCCCC(=O)NCCN(CCC(=O)O)CCO		Sirius	
864	552,4767	18,13	551,4693		H+		Lipid and lipid-like molecules	Fatty acyls	Sphingolipids		Fatty acids		C34H65NO4			Sirius	
868	654,5432	18,22		631,5632	Na+		Lipid and lipid-like molecules	Fatty acyls	Fatty amides		Fatty acids		C35H73N3O6			Sirius	
869	326,3373	18,66	325,3299	303,3573													
870	298,3060	18,76	297,2986		H+	297,4	Lipid and lipid-like molecules	Fatty acyls	Fatty amides		Fatty acids	Nonadecanamide	C19H39NO	CCCCCCCCCCCCCCCCCCC(=O)N		Sirius	
873	469,3806	18,87	468,3732	446,4006													
882	192,0522	0,97		169,0722	Na+		Organic acids and derivatives	Boronic acid derivatives			Amino acids		C4H9B2N2O2S			Sirius	
884	657,1724	11,63		634,1924	Na+	634,63	Benzenoids	Anthracenes	Anthraquinones		Polyketides	1-(4,5-Dihydroxy-2-methoxy-7-methyl-9,10-dioxoanthracen-1-yl)-4,5-dihydroxy-2-methoxy-7-methyl-3-(3-methylbut-1-enyl)anthracene-9,10-dione	C37H30O10	CC1=CC2=C(C(=C1)O)C(=O)C3=C(C2=O)C(=C(C=C3O)OC)C4=C(C(=C(C5=C4C(=O)C6=C(C5=O)C(=CC(=C6)C)O)O)C=CC(C)C)OC		Sirius	
885	503,2580	11,50	502,2506		H+	502,25	Lipid and lipid-like molecules	Steroids and steroid derivatives	Steroids esters		Terpenoids	Andrastone C	C28H38O8	CC1=CC2C3(C(C(C4C2(CCC(C4(C)C)OC(=O)C)C=O)O)OC5=C(C(=O)C1(C53C(=O)OC)C)C)C	Fungus (Penicillium sp.)	Sirius	10.1016/j.fitote.2018.12.022
886	408,1511	12,50	407,1437	385,1711			Organic acids and derivatives	Carboxylic acids and derivatives	Amino acids, peptides and analogues	Amino acids and derivatives	Amino acids and peptides		C15H23N5O7			Sirius	
889	72,0423	14,39	71,0349	49,0623													
890	482,3994	14,79	481,3920		H+								C27H51N3O4			Sirius	
891	438,3737	14,85		415,3937	Na+								C26H45N3O			Sirius	
892	443,3290	14,87	442,3216		H+		Lipid and lipid-like molecules	Fatty acyls	Fatty acid esters		Polyketides		C28H42O4			Sirius	
893	399,3032	14,92	398,2958		H+								C27H42O2			Sirius	
898	263,1605	8,73		240,1805	Na+		Lipid and lipid-like molecules	Prenol lipids			Terpenoids		C14H24O3			Sirius	
899	435,1599	11,62		412,1799	Na+	412,4	Organoheterocyclic compounds	Lactone	Gamme butyrolactones		Sesquiterpenoids	5,9,10-trihydroxy-3'-(1-hydroxyethyl)-8-methoxy-7-methyl-10-propan-2-ylspiro[3-oxatricyclo[5.3.1.0^{4,11}]undec-1(11)-ene-6,5'-oxolane]-2,2'-dione	C20H28O9	CC(C)C1(C(C(C2(C3=C1C(=O)OC3C(C24CC(C(=O)O4)C(C)O)O)C)OC)O)O	Eukaryota	Sirius	
906	91,0519	8,21	90,0445	68,0719									C5H8			Sirius	
915	83,0470	2,02	82,0396	60,0670									C3H8O			Sirius	

917	191,0667	2,03	190,0593		H+	190,063	Phenylpropanoids and polyketides	Coumarins and derivatives	Isocoumarins		Shikimates and phenylpropanoids	7-hydroxy-3, 5-dimethyl-isochromen-1-one	C11H10O3	CC1=CC2=C(C=C(C=C2C(=O)O1)O)C	Fungus	Sirius	10.1016/j.fitote.2011.10.013
929	197,1255	5,20	196,1181		H+	196,12	Organic acids and derivatives	Carboxylic acids and derivatives	Amino acids, peptides, and analogues	Amino acids and derivatives	Amino acids and peptides	Cyclo(Pro-Val)	C10H16N2O2	C1CC(N(C1)CC(CO)N)CO	Fungus (Aspergillus sp.)	GNPS	10.7164/antibiotics.54.179
932	241,2000	7,23	240,1926		H+								C12H24N4O			Sirius	
933	182,0239	7,46		159,0439	Na+		Organic acids and derivatives	Carboxylic acids and derivatives	Amino acids, peptides and analogues	Amino acids and derivatives	Amino acids and peptides		C6H9NO2S			Sirius	
934	245,1247	7,46	244,1173		H+	244,29	Organic acids and derivatives	Carboxylic acids and derivatives	Amino acids, peptides, and analogues	Amino acids and derivatives	Amino acids and peptides	Cyclo(Phe-Pro)	C14H16N2O2	C1CC2C(=O)NC(C(=O)N2C1)CC3=CC=CC=C3	Bacteria	GNPS	10.1021/np030233e
935	213,1560	7,71	212,1486		H+	212,15	Organic acids and derivatives	Carboxylic acids and derivatives	Amino acids, peptides, and analogues	Amino acids and derivatives	Amino acids and peptides	Cyclo(Val-Leu)	C11H20N2O2	CC(C)CC1C(=O)NC(C(=O)N1)C(C)C	Fungus (Penicillium sp.)	Npatlas	10.1080/10286020.2013.780349
939	288,2852	9,73	287,2778		H+		Lipid and lipid-like molecules	Fatty acyls	Fatty amides		Fatty acids		C13H33N7			Sirius	
943	343,2908	11,22	342,2834	320,3108	Na+		Lipid and lipid-like molecules	Prenol lipids			Terpenoids		C20H38BO2			Sirius	
945	219,1709	11,65		196,1909	Na+	196,33	Organic oxygen compounds	Organooxygen compounds	Carbonyl compounds	Ketones	Fatty acids	Cyclohexanone	C13H24O	CCCCCCCC1CCC(=O)CC1		Sirius	
946	405,2615	12,04	404,2541		H+	404,25	Lipid and lipid-like molecules	Steroids and steroid derivatives	Bile acids, alcohols and derivatives	Hydroxy bile acids, alcohols and derivatives	Terpenoids	12α-Hydroxy-3,7-diketocholanic acid	C24H36O5	CC(CCC(=O)O)C1CCC2C1(C(CC3C2C(=O)CC4C3(CCC(=O)C4)C)O)C	Bacteria (Psychrobacter sp.)	Npatlas	10.1007/s12272-009-1607-
947	227,1243	12,40		204,1443	Na+	204,26	Organic acid and derivatives	Hydroxy acids and derivatives	Medium-chain hydroxy acids and derivatives		Fatty acids	3,5-dihydroxydecanoic acid	C10H20O4	CCCCCC(CC(CC(=O)O)O)O	Fungus	Sirius	10.3390/molecules24224051
949	371,3228	12,65	370,3154		H+		Lipid and lipid-like molecules	Prenol lipids					C22H38BN3O			Sirius	
950	288,2494	12,78	287,2420		H+	287,44	Lipid and lipid-like molecules	Fatty acyls	Fatty amides		Fatty acids	N,N-bis(2-hydroxyethyl)dodecanamide	C12H29N7O	CCCCCCCCCCCC(=O)N(CCO)CCO		GNPS	
958	390,2442	14,58	389,2368	367,2642													
959	425,3683	14,55		402,3883	Na+		Lipid and lipid-like molecules	Prenol lipids					C24H42B3N3			Sirius	
960	319,2797	15,29		296,2997	Na+		Lipid and lipid-like molecules	Fatty acyls	Fatty acids		Fatty acids		C19H36O2			Sirius	
962	613,4695	18,17	612,4621		H+		Organic acids and derivatives	Carboxylic acids and derivatives	Amino acids, peptides and analogues	Amino acids and derivatives			C36H60N4O4			Sirius	
963	298,3062	18,38	297,2988		H+	297,4	Lipid and lipid-like molecules	Fatty acyls	Fatty amides		Fatty acids	Nonadecanamide	C19H39NO	CCCCCCCCCCCCCCCCCCC(=O)N		Sirius	
964	326,3372	18,40	325,3298		H+	325,5	Lipid and lipid-like molecules	Fatty acyls	Fatty amides		Fatty acids	14-oxoicosanamide	C20H39NO2	CCCCCCC(=O)CCCCCCCCCCCCC(=O)N		Sirius	

965	406,2216	18,59	405,2142	383,2416			Organoheterocyclic compounds	Diazanaphthalenes	Benzodiazines		Alkaloids		C21H29N5O2			Sirius	
966	199,0544	1,09	198,0470		H+		Organic acids and derivatives	Organic sulfuric acids and derivatives	Sulfuric acid diamides				C5H11FN2O3S			Sirius	
969	194,0894	9,53		171,1094	Na+		Organic acids and derivatives	Carboxylic acids and derivatives	Amino acids, peptides and analogues	Amino acids and derivatives	Amino acids and peptides		C7H13N3O2			Sirius	
970	292,2230	15,76	291,2156		H+		Organic acids and derivatives	Carboxylic acids and derivatives	Amino acids, peptides and analogues	Amino acids and derivatives	Amino acids and peptides		C13H29N3O4			Sirius	
973	270,2753	17,12	269,2679		H+	269,27	Organic acids and derivatives	Carboxymidic acids and derivatives	Carboxymic acids			Bacillamidin G	C17H35NO	CCC(C)CCCCCCCCCCCC(=O)N	Bacteria	Sirius	10.3390/md16090326
975	312,3211	18,45	311,3137	289,3411			Lipid and lipid-like molecules	Fatty acyls	Fatty amides		N-acyl amines					Sirius	
983	269,1340	8,51		246,1540	Na+		Lipid and lipid-like molecules	Fatty acyls	Fatty acids and conjugated		Fatty acids		C15H1803			Sirius	
987	280,2589	9,53		257,2789	Na+		Organic acids and derivatives	Carboxylic acids and derivatives	Amino acids, peptides and analogues	Amino acids and derivatives	Amino acids and peptides		C16H25NO3			Sirius	
988	231,1089	9,69		208,1289	Na+	208,1099	Benzenoids	Phenols	Benzenediols	Cathechols	Polyketides	5-(Hydroxymethyl)-3-(3-methylbut-2-enyl)benzene-1,2-diol	C12H16O3	CC(=CCC1=C(C(=CC(=C1)CO)O)O)C	Fungus	Npatlas	10.1016/0031-9422(95)00165-4
989	307,1066	9,69	306,0992		H+	306,1103	Organoheterocyclic compounds	Benzopyrans	1-benzopyrans	Butenolides	polyketides	Asperscleroti orone F	C16H18O6	CC1CC2=C(C(=C(C=C2OC13C(=CC(=O)O3)OC)OC)O)C	Fungus (Aspergillus sp.)	Npatlas	10.1016/j.phytochem.2017.02.008
990	203,1029	9,79		180,1229	Na+	180,23	Organoheterocyclic compounds	Azoles	Imidazoles		Alkaloids	2-amino-1-(1H-imidazol-5-yl)-4-methylpentan-3-one	C9H14N3O	CC(C)C(=O)C(CC1=CN=CN1)[NH]		Sirius	
991	251,1235	9,81		228,1435	Na+	228,28	Phenylpropanoids and polyketides	Macrolides and analogues			Monoterpenoids	(10S,12S)-10-hydroxy-12-methyloxacyclododecane-2,5-dione	C12H20O4	CC1CC(CCCCC(=O)CCC(=O)O1)O	Fungus	Sirius	10.1002/hlca.200690055
992	288,2852	9,93	287,2778		H+		Lipid and lipid-like molecules	Fatty acyls	Fatty amides		Fatty acids		C13H33N7			Sirius	
993	265,1028	10,26	264,0954	242,1228	Na+		Organic oxygen compounds	Organooxygen compounds	Carbonyl compounds		Monoterpenoids		C13H16F2O2			Sirius	
994	261,1192	10,82		238,1392	Na+	260,1049	Phenylpropanoids and polyketides	Coumarins and derivatives	Hydroxycoumarins	Hydroxycoumarins		7-(gamma,gamma-Dimethylallyloxy)-6-hydroxy-4-methylcoumarin	C15H16O4	CC1=CC(=O)OC2=CC(=C(C=C12)O)OCC=C(C)C	Fungus	GNPS	10.1007/s10600-015-1252-5
996	244,1658	11,75	243,1584		H+	221,34	Organoheterocyclic compounds	Quinolines and derivatives	Quinolones and derivatives	Hydroquinolones	Alkaloids	2-heptylquinolin-4(1H)-one	C16H21NO	CCCCCCCC1=CC(=O)C2=CC=CC=C2N1	Bacteria	GNPS	10.3390/md17020133
997	272,1968	13,40	271,1894		H+	271,4	Organoheterocyclic compounds	Quinolines and derivatives	Hydroquinolines		Alkaloids	2-nonylquinolin-4(1H)-one	C18H25NO	CCCCCCCCCC1=CC(=O)C2=CC=CC=C2N1	Bacteria (Pseudomonas sp.)	GNPS	10.1007/PL00011792
998	256,2594	16,88	255,2520		H+	255,43	Lipid and lipid-like molecules	Fatty acyls	Fatty amides		Fatty acids	Hexadecanamide	C16H33NO	CCCCCCCCCCCCCCCC(=O)N	Fungus	GNPS	10.1016/0031-9422(81)85182-5

999	324,3218	18,92	323,3144	301,3418			Organic acids and derivatives	Carboxylic acids and derivatives	Amino acids, peptides and analogues	Amino acids and derivatives	Amino acids and peptides					Sirius	
1003	199,0833	5,46	198,0759		H+	198,22	Organoheterocyclic compounds	Quinolines and derivatives	Isoquinolones and derivatives		Alkaloids	3-methyl-1H-pyrazolo[1,5-b]isoquinolin-9-one	C12H10N2O	CC1=CNN2C1=CC3=CC=CC=C3C2=O	Bacteria	Npatlas	10.7164/antibiotics.49.700
1005	544,3065	9,26			H+		Organoheterocyclic compounds	Naphthopyrans	Diterpenoids		Alkaloids					Sirius	
1007	377,1772	10,13	376,1698		H+		Lipid and lipid-like molecules	Fatty acyls	Fatty acids and conjugated		Fatty acids		C21H28O6			Sirius	
1008	248,0882	10,89		225,1082	Na+								C11H15NO4			Sirius	
1010	354,1655	11,77	353,1581	331,1855													
1016	183,0907	5,98	182,0833		H+	182,22	Organoheterocyclic compounds	Indoles and derivatives	Indoles	Beta carboline	Alkaloids	3-methyl-β-carboline	C12H10N2	CC1=CC2=C(C=N1)NC3=CC=CC=C32	Bacteria (Nocardia sp.)	GNPS	10.1271/bbb1961.51.921
1017	164,0701	6,72	163,0627		H+	163,17	Benzenoids	Benzene and substituted derivatives	Styrenes		Shikimates and phenylpropanoids	N-(4-hydroxystyryl) formamide	C9H9NO2	C1=CC(=CC=C1/C=C\NC=O)O	Fungus (Aspergillus sp.)	Sirius	10.7164/antibiotics.37.469
1018	263,1637	8,74	262,1563		H+	262,3447	Lipid and lipid-like molecules	Prenol lipids	Sesquiterpenoids		Terpenoids	Methyl 2-[(1r,4ar,6s,8ar)-6-hydroxy-4,7-dimethyl-1,2,4a,5,6,8a-hexahydronaphthalen-1-yl]prop-2-enoate	C16H22O3	C=C(C(=O)OC)C1CC=C(C)C2CC(O)C(C)=CC12	Eukaryota	Sirius	10.1016/S0040-4020(01)88464-7
1022	359,0760	9,52		336,0960	Na+	336,9	Polycyclic aromatic polyketides	Anthraquinones and anthrones	Anthroquinones		Polyketides	Methyl 3,6,8-trihydroxy-4-methoxy-1-methyl-9,10-dioxoanthracene-2-carboxylate	C16H16O8	CC1=C2C(=C(C(=C1C(=O)OC)O)OC)C(=O)C3=C(C2=O)C(=CC(=C3)O)O	Fungus	Sirius	10.1016/0021-9673(93)80481-m
1023	216,1954	9,57	215,1880		H+	215,3329	Lipid and lipid-like molecules	Fatty acyls	Fatty acids and conjugated		Fatty acids	12-aminododecanoic acid	C12H25NO2	NCCCCCCCCCCCC(=O)O	Bacteria (Pseudomonas sp.)	Sirius	10.1111/1751-7915.12278
1024	288,2528	9,75	287,2454		H+	287,44	Organic acids and derivatives	Carboxylic acids and derivatives	Amino acids, peptides and analogues	Alpha amino acids and derivatives	Fatty acids	N-dodecyl-N-(2-hydroxyethyl)glycine	C16H33NO3	CCCCCCCCCCCCN(CCO)CC(=O)O		Sirius	
1025	255,0652	10,48	254,0578		H+	254,2381	Phenylpropanoids and polyketides	Isoflavonoids	Isoflav-2-enes	Isoflavones	Shikimates and phenylpropanoids	daidzein	C15H10O4	C1=CC(=CC=C1C2=COC3=C(C2=O)C=CC(=C3)O)O	Bacteria	GNPS	10.1515/znb-2003-0713
1027	211,0859	11,36	210,0785		H+	210,23	Organoheterocyclic compounds	Indoles and derivatives	Carboline alkaloids	β-carboline	Alkaloids	1-acetyl-β-carboline	C13H10N2O	CC(=O)C1=NC=CC2=C1NC3=CC=CC=C23	Bacteria	Sirius	10.1002/abio.370100405
1031	405,2671	12,05	404,2597		H+	404,25	Lipid and lipid-like molecules	Steroids and steroid derivatives	Bile acids, alcohols and derivatives	Hydroxy bile acids, alcohols and derivatives	Terpenoids	12α-Hydroxy-3,7-diketocholanic acid	C24H36O5	CC(CCC(=O)O)C1CCC2C1(C(CC3C2C(=O)CC4C3(CCC(=O)C4)C)O)C	Bacteria (Psychrobacter sp.)	Npatlas	10.1007/s12272-009-1607-
1032	226,1222	12,56	225,1148		H+	225,11	Organoheterocyclic compounds	Pyridines and derivatives			Alkaloids	4-phenyl-3-(pyridine-2-yl)but-2-en-1-ol	C15H15NO	C1=CC=C(C=C1)C/C(=C\CO)/C2=CC=CC=N2	Bacteria	Sirius	10.1016/j.bmcl.2006.08.015

1034	330,2627	13,72	329,2553		H+		Organic acids and derivatives	Carboxylic acids and derivatives	Amino acids, peptides and analogues	Amino acids and derivatives	Alkaloids		C18H35NO4			Sirius	
1042	180,0622	7,06	179,0548		H+	179,058	Organic acids and derivatives	Carboxylic acids and derivatives	Amino acids, peptides and analogues	Amino acids and derivatives	Shikimates and phenylpropanoids	Erbstatin	C9H9NO3	CC(=CC(=O)NCC(=O)O)C	Bacteria	Sirius	10.7164/antibiotics.39.170
1044	657,1741	11,92		634,1941	Na+	634,63	Benzenoids	Anthracenes	Anthraquinones		Polyketides	1-(4,5-Dihydroxy-2-methoxy-7-methyl-9,10-dioxoanthracen-1-yl)-4,5-dihydroxy-2-methoxy-7-methyl-3-(3-methylbut-1-enyl)anthracene-9,10-dione	C37H30O10	CC1=CC2=C(C(=C1)O)C(=O)C3=C(C2=O)C(=C(C=C3O)OC)C4=C(C(=CC5=C4C(=O)C6=C(C5=O)C(=CC(=C6)C)O)O)C=CC(C)OC		Sirius	
1046	368,4205	14,37	367,4131	345,4405			Lipid and lipid-like molecules	Fatty acyls	Fatty amides		Fatty acids					Sirius	
1050	351,2390	10,57	350,2316	328,2590													
1052	556,3646	11,15	555,3572		H+	555,31	Organoheterocyclic compounds	Naphthopyrans			Terpenoids	(1S,2R,5S,7S,8R,9R,11S,12S,15S)-21-(2,3-dihydroxy-3-methylbutyl)-7-(2-hydroxypropan-2-yl)-1,2-dimethyl-6,10-dioxa-24-azaheptacyclo[13.10.0.02,12.05,11.09,11.017,25.018,23]pentacosa-17(25),18(23),19,21-tetraene-8,12-diol	C32H45NO7	CC12CCC3C4(C1(CCC5C2(C6=C(C5)C7=C(N6)C=C(C=C7)CC(C(C)(C)O)O)C)O)C(O4)C(C(O3)C(C)(C)O)O	Fungus	Npatlas	10.1002/anie.200603821
1053	334,1510	11,46	333,1436	311,1710													
1054	399,2054	12,25		376,2254	Na+	377	Lipid and lipid-like molecules	Steroids and steroid derivatives	Pregnane steroids		Terpenoids	1-(6-Chloro-17-ethyl-3-hydroxy-10,13-dimethyl-1,2,3,8,9,11,12,14,15,16-decahydrocyclopenta[a]phenanthren-17-yl)ethanone	C23H33ClO2	CCC1(CCC2C1(CCC3C2C=C(C4=CC(CCC34C)O)Cl)C)C(=O)C		Sirius	
1055	204,1357	12,53		181,1557	Na+		Alkaloids and derivatives	Quinolizidine alkaloids			Alkaloids		C11H19NO			Sirius	
1056	305,1641	13,16	304,1567		H+	304,4	Organoheterocyclic compounds	Indoles and derivatives	Carboline alkaloids	β-carboline	Alkaloids	4-Hydroxymethyl-3-(2,4-dimethyl- 7-indolyl)-2-methylindole	C20H20N2O	CC1=C2C=C(NC2=C(C=C1)C3=C(NC4=CC=CC(=C43)CO)C)C	Fungus	Sirius	10.3891/acta.chem.scand.50-0303
1058	319,1794	13,98	318,1720		H+	318,413	Organoheterocyclic compounds	Indoles and derivatives	Carboline alkaloids	β-carboline	Alkaloids	4-(methoxymethyl)-2-methyl-3-[(2-methyl-1h-indol-4-yl)methyl]-1h-indole	C21H22N2O	CC1=CC2=C(C=CC=C2N1)CC3=C(NC4=CC=CC(=C43)COC)C	Fungus	Sirius	10.3891/acta.chem.scand.50-0303
1060	467,2667	14,36		444,2867	Na+	445,5	Lipid and lipid-like molecules	Steroids and steroid derivatives	Steroids esters		Terpenoids	[(3S,8R,9S,10R,13S,14S,17R)-17-(2-chloroethynyl)-13-ethyl-1,2,3,6,7,8,9,10,11,12,14,15,16,17-tetradecahydrocyclopenta[a]phenanthren-	C28H41ClO2	CCCCCCC(=O)OC1CCC2C3CCC4(C(CCC4C3CCC2=C1)C#CCl)CC		Sirius	

												3-yl] heptanoate					
1062	383,2100	14,53	382,2026	360,2300			Lipid and lipid-like molecules	Steroids and steroid derivatives	Hydroxysteroids		Terpenoids					Sirius	
1063	333,1948	14,78	332,1874		H+	332,1889	Organoheterocyclic compounds	Diazanaphthalenes	Benzodiazines	Quinoxalines	alkaloids	Lavanducyanin	C22H24N2O	CC1=C(CCC(C1)(C)C)CN2C3=CC=CC=C3N=C4C2=CC=CC4=O	Bacteria	Sirius	10.7164/antibiotics.42.1196
1064	301,2119	14,80	300,2045		H+	300,4	Lipid and lipid-like molecules	Prenol lipids	Diterpenoids		Terpenoids	dehydroabietic acid	C20H28O2	CC(C)C1=CC2=C(C=C1)C3(CCCC(C3CC2)(C)C(=O)O)C	Eukaryota	GNPS	10.1016/j.phymed.2008.02.020
1066	347,2101	15,56		324,2301	Na+	324,5	Lipid and lipid-like molecules	Fatty acyls	Fatty alcohols		Polyketides	Pironetin	C19H32O4	CCC1C=CC(=O)OC1CC(C(C)C(C(C)CC=CC)OC)O	Bacteria	Sirius	10.7164/antibiotics.47.697
1069	360,1568	16,30	359,1494	337,1768													
1071	376,1511	16,33	375,1437	353,1711													
1075	282,2747	16,44		259,2947	Na+								C16H33B2N			Sirius	
1078	603,3900	17,80	602,3826	580,4100			Lipid and lipid-like molecules	Steroids and steroid derivatives	Hydroxysteroids		Terpenoids					Sirius	
1082	623,6380	19,32	622,6306	600,6580													
1086	185,0677	9,56		162,0877	Na+	162,13	Organoheterocyclic compounds	Diazines	Pyrazines		Alkaloids	1,5-dimethyl-6H-pyrrolo[2,3-c]pyridin-7-one	C9H10N2O	CC1=CC2=C(C(=O)N1)N(C=C2)C		Sirius	
1091	247,1294	9,58		224,1494	Na+	224,14	Organoheterocyclic compounds	Pyrans	Pyranones and derivatives		Polyketides	Nipyrone A	C13H20O3	CCC(C)CC(C)C1=CC(=C(C(=O)O1)C)O	Fungus (Aspergillus sp.)	Npatlas	10.3390/md17060344
1094	217,1553	12,12		194,1753	Na+	194,16	Lipid and lipid-like molecules	Prenol lipids	Sesquiterpenoids		Terpenoids	Dihydro-b-ionone	C13H22O	CC(=O)CCC1=C(C)CCCC1(C)C	Bacteria	Npatlas	10.2166/wst.2004.0531
1095	304,0285	14,06		281,0485	Na+		Benzenoids	Benzene and substituted derivatives	Benzoyl derivatives		Alkaloids		C13H16BrNO			Sirius	
1096	306,0265	14,08	305,0191		H+		Benzenoids	Phenol ethers	Anisoles		Alkaloids		C12H13Cl2NO4			Sirius	
1104	265,1400	8,73	264,1326	242,1600													
1106	393,1718	11,01		370,1918	Na+		Lipid and lipid-like molecules	Steroids and steroid derivatives	Terpenoids		Terpenoids		C19H30O5S			Sirius	
1107	172,1666	11,05	171,1592		H+	171,28	Lipid and lipid-like molecules	Fatty acyls	Fatty amides		Fatty acids	Decanamide	C10H21NO	CCCCCCCCCC(=O)N		GNPS	
1115	387,2511	12,04	386,2437	364,2711													
1121	358,2730	12,75	357,2656	335,2930			Lipid and lipid-like molecules	Fatty acyls	Fatty amides		Fatty acids		C23H32NO2			Sirius	
1131	315,1920	14,05	314,1846		H+	314,4	Lipid and lipid-like molecules	Prenol lipids	Diterpenoids		Terpenoids	1,4a-dimethyl-9-oxo-7-propan-2-yl-3,4,10,10a-tetrahydro-2H-phenanthrene-1-	C20H26O3	CC(C)C1=CC2=C(C=C1)C3(CCCC(C3CC2=O)(C)C(=O)O)C	Eukaryota	GNPS	

												carboxylic acid					
1136	326,3025	15,47	325,2951	303,3225			Lipid and lipid-like molecules	Fatty acyls	Fatty amides		Fatty acids		C14H37N7			Sirius	
1137	362,2781	15,51	361,2707	339,2981			Lipid and lipid-like molecules	Fatty acyls	Fatty amides		Fatty acids		C19H39NO5			Sirius	
1139	359,2398	16,27	358,2324	336,2598													
1141	749,4436	17,47	748,4362	726,4636													
1143	397,3249	17,71	396,3175		H+		Lipid and lipid-like molecules	Fatty esters	Lactonase		Fatty acids		C24H44O4			Sirius	
1144	312,3216	17,93	311,3142		H+	311,5	Lipid and lipid-like molecules	Fatty acyls	Fatty amides		N-acyl amines	Icosanamide	C20H41NO	CCCCCCCCCCCCCCCCCCCC(=O)N		Sirius	
1145	350,3399	18,99	349,3325	327,3599													
1149	372,1140	1,00	371,1066	349,1340									C13H17N5O8			Sirius	
1165	320,2545	16,62	319,2471		H+		Organoheterocyclic compounds	Triazines	Aminotriazines		Alkaloids		C16H29N7			Sirius	
1166	296,2910	17,17	295,2836		H+		Organic nitrogen compounds	Organonitrogen compounds	Guanidines		Alkaloids		C17H35N4			Sirius	
1169	338,3373	18,52	337,3299	315,3573			Lipid and lipid-like molecules	Fatty acyls	Fatty amides		N-acyl amines					Sirius	
1176	256,1425	14,35		233,1625	Na+		Benzenoids	Benzene and substituted derivatives	Benzoic acids and derivatives	Benzamides	Alkaloids		C13H19N3O			Sirius	
1182	377,3217	14,92		354,3417	Na+								C25H38O			Sirius	
1183	394,3481	14,95		371,3681	Na+		Lipid and lipid-like molecules	Fatty acyls	Fatty amides		Fatty acids		C23H46FNO			Sirius	
1184	333,2958	15,01		310,3158	Na+		Lipid and lipid-like molecules	Fatty acyls	Fatty acids		Fatty acids		C20H38O2			Sirius	
1188	124,0374	1,35	123,0300		H+	123,11	Organoheterocyclic compounds	Pyridines and derivatives	Pyridinecarboxylic acids and derivatives	Pyridinecarboxylic acids	Alkaloids	Pyridine-3-carboxylic acid	C6H5NO2	C1=CC(=CN=C1)C(=O)O	Fungus	GNPS	10.1021/jf052890d
1191	195,1095	6,72	194,1021		H+	194,094	Organoheterocyclic compounds	Coumarins and derivatives			Shikimates and phenylpropanoids	2,3,4-Trimethyl-5,7-dihydroxy-2,3-dihydrobenzofuran	C11H14O3	CC1C(OC2=C(C=C(C(=C12)C)O)O)C	Fungus (Penicillium sp.)	Npatlas	10.1021/np010605o
1194	366,1403	8,65	365,1329	343,1603													
1195	464,1869	9,05		441,2069	Na+	441,47	Organoheterocyclic compounds	Indoles and derivatives	Pyridoindoles	Beta carboline	Alkaloids	Cyclotryprostatin F	C23H27N3O6	CC(=CC1C2=C(C(C3(N1C(=O)C4(CCCN4C3=O)O)O)OC)C5=C(N2)C=C(C=C5)OC)C	Fungus (Penicillium sp.)	Npatlas	10.3390/md17090514
1196	292,2591	9,91		269,2791	Na+	269,47	Lipid and lipid-like molecules	Fatty acyls	Fatty amides		Fatty acids	N-(13-methyltetradecyl)ethanimidic acid	C17H35NO	CC(O)=NCCCCCCCCCCCC(C)C	Eukaryota	Sirius	
1197	306,2745	10,36		283,2945	Na+	283,287	Lipid and lipid-like molecules	Fatty acyls	Fatty amides		Fatty acids	N-isopentyltridecanamide	C18H37NO	CCCCCCCCCCCCC(=O)NCCC(C)C	Bacteria	Npatlas	10.1007/s00284-015-0780-3

1200	########	10,49	1024,4005		H+		Organic acids and derivatives	Carboxylic acids and derivatives	Amino acids, peptides and analogues	Amino acids and derivatives	Amino acids and peptides		C40H68N2O28			GNPS	
1202	277,1390	11,83	276,1316		H+	276,28	Organoheterocyclic compounds	Coumarins and derivatives	Coumarines		Coumarines	Visamminol	C15H16O5	CC1=CC(=O)C2=C(C3=C(C=C2O1)OC(C3)C(C)(C)O)O	Eukaryota	GNPS	10.1016/0031-9422(95)00625-7
1206	324,2855	15,36	323,2781		H+	323,5	Organic acids and derivatives	Carboxymidic acids and derivatives	Carboximic acids		Fatty acids	Linoleylethanolamide	C20H37NO2	CCCCCC=CCC=CCCCCCCCC(=O)NCCO	Eukaryota	GNPS	
1207	339,2275	15,91		316,2475	Na+	316,5	Benzenoids	Phenols	Benzenediols	Resorcinols	Polyketides	2,4-Bis(3-methylbut-2-enyl)-5-pentylbenzene-1,3-diol	C21H32O2	CCCCCC1=CC(=C(C(=C1CC=C(C)C)O)CC=C(C)C)O		Sirius	
1209	739,4528	16,32	738,4454	716,4728													
1211	339,2277	17,41		316,2477	Na+	316,5	Benzenoids	Phenols	Benzenediols	Resorcinols	Polyketides	2,4-Bis(3-methylbut-2-enyl)-5-pentylbenzene-1,3-diol	C21H32O2	CCCCCC1=CC(=C(C(=C1O)CC=C(C)C)O)CC=C(C)C		Sirius	
1215	425,3000	18,30	424,2926	402,3200	Na+		Lipid and lipid-like molecules	Prenol lipids	Diterpenoids		Terpenoids		C26H42O3			Sirius	
1216	447,2816	18,30	446,2742		H+	446,6	Organic acids and derivatives	Carboxylic acids and derivatives	Amino acids, peptides and analogues	Peptides	Amino acids and peptides	Sclerotiotide C	C24H38N4O4	CC=CC=CC=CC(=O)NC1CCCCNC(=O)C(N(C(=O)C(N(C1=O)C)C(C)C)C)C	Fungus (Aspergillus sp.)	Npatlas	10.1021/np100198h
1217	492,1964	18,50	491,1890	469,2164													
1249	211,1404	6,60	210,1330		H+	210,13	Organic acids and derivatives	Carboxylic acids and derivatives	Amino acids, peptides and analogues	Amino acids and derivatives	Amino acids and peptides	Cyclo(Leu-Pro)	C11H18N2O2	CCC(C)C1C(=O)N2CCCC2C(=O)N1	Bacteria	GNPS	10.1021/np030233e
1250	166,1089	9,05	165,1015		H+	165,11	Benzenoids	Phenols ethers	Aminiophenyl ethers		Alkaloids	N-methyl-2-(2-methylpropoxy)aniline	C10H15NO	CC(C)COC1=CC=CC=C1N	Bacteria (Brevibacterium sp.)	Sirius	10.3389/fmicb.2015.01235
1251	376,1604	11,04		353,1804	Na+	353,4	Organoheterocyclic compounds	Diazanaphtalenes	Benzodiazines	Quinazolines	Amino acids and peptides	3-(2-(1-(2-methoxyethyl)-2,5-dimethyl-1H-pyrrol-3-yl)-2-oxoethyl)-2-methylquinazolin-4(3H)-one	C20H33N3O3	CC1=CC(=C(N1CCOC)C)C(=O)CN2C(=NC3=CC=CC=C3C2=O)C		Sirius	
1252	394,1706	11,04		371,1906	Na+	371,39	Organoheterocyclic compounds	Imidazopyrimidines	Purines and purines derivatives	Purinones		1-(2,2-Diethoxyethyl)-9-[(1,3-dihydroxy-2-propoxy)methyl]guanine	C15H25N5O6	CCOC(CN1C(=O)C2=C(N=C1N)N(C=N2)COC(CO)CO)OCC		Sirius	
1259	319,1351	9,60	318,1277		H+	318,32	Phenylpropanoids and polyketides	Isochromanequinones	Benzoisochromanequinones		Polyketides	Astropaquinone B	C17H18O6	CC1CC2=C(C(O1)OC)C(=O)C3=C(C2=O)C=C(C=C3OC)OC	Fungus	Npatlas	10.1055/s-0029-1185684
1268	393,0296	1,55	392,0222	370,0496													
1273	213,0985	5,99	212,0911		H+	212,25	Alkaloids and derivatives	Harmala alkaloids			Alkaloids	Cordysinin C	C13H12N2O	CC(C1=NC=CC2=C1NC3=CC=CC=C23)O	Fungus	Lotus	10.1021/np100902f
1274	218,0950	6,37		195,1150	Na+	195,21	Benzenoids	Benzene and substituted derivatives	Benzoic acids and derivatives	Hydroxybenzoic acid derivatives	Amino acids and peptides	2-hydroxy-N-(1-hydroxypropan-2-yl)benzamide	C10H13NO3	CC(CO)NC(=O)C1=CC=CC=C1O	Bacteria	Npatlas	10.1016/j.phytochem.2004.06.010

1277	392,2028	7,17	391,1954		H+	391,5	Organoheterocyclic compounds	Indoles and derivatives	Pyrrolindoles	Pyridoindolones	Alkaloids	Roquefortine D	C22H25N5O2	CC(C)(C=C)C12CC3C(=O)NC(C(=O)N3C1NC4=CC=CC=C24)CC5=CN=CN5	Fungus (Penicillium sp.)	Sirius	10.1016/j.tet.2005.05.026
1278	420,1612	7,36	419,1538		H+	419,3	Organoheterocyclic compounds	Indoles and derivatives	Pyrrolindoles	Pyridoindolones	Alkaloids	Glandicoline B	C22H21N5O4	CC(C)(C=C)C12C=C(C(=O)N3C1(NC(=O)C3=CC4=CN=CN4)N(C5=CC=CC=C25)O)O	Fungus (Penicillium sp.)	GNPS	10.1016/j.chembiol.2011.08.012
1281	527,2801	8,09	526,2727		H+	526,27	Organic acids and derivatives	Carboxylic acids and derivatives	Amino acids, peptides and analogues		Amino acids and peptides	Bilaid B/C (phenylalanyl--valyl-valyl-tyrosine)	C28H38N4O6	CC(C)C(C(=O)NC(C(C)C)C(=O)NC(CC1=CC=C(C=C1)O)C(=O)O)NC(=O)C(CC2=CC=CC=C2)N	Fungus (Penicillium sp.)	Npatlas	10.1073/pnas.1908662116
1282	566,2905	8,24		543,3105	Na+		Lipid and lipid-like molecules	Steroids and steroid derivatives	Terpenoids		Alkaloids		C31H35NO9			Sirius	
1284	541,2956	8,47		518,3156	Na+	518,6	Organic acids and derivatives	Carboxylic acids and derivatives	Amino acids, peptides and analogues	Amino acids and derivatives	Amino acids and peptides	Lysyl-alanyl-threonyl-threonyl-valine	C22H42N6O8	CC(C)C(C(=O)O)NC(=O)C(C(C)O)NC(=O)C(C(C)O)NC(=O)C(C)NC(=O)C(CCCCN)N		Sirius	
1285	477,3014	8,61		454,3214	Na+	476,26	Organic acids and derivatives	Carboxylic acids and derivatives	Amino acids, peptides and analogues	Amino acids and derivatives	Amino acids and peptides	Violaceomide A	C24H36N4O6	CCC(C)C1C(=O)N(C(C(=O)NC(C(=O)NC(C(=O)N1)C(C)O)CC2=CC=C(C=C2)OC)C)C	Fungus (Aspergillus sp.)	Npatlas	10.3389/fchem.2018.00226
1286	550,2962	9,01	549,2888		H+		Lipid and lipid-like molecules	Steroids and steroid derivatives	Terpenoids		Alkaloids		C32H39NO7			Sirius	
1287	367,1280	9,76		344,1480	Na+	344,4	Organic acids and derivatives	Carboxylic acids and derivatives	Amino acids, peptides and analogues	Alpha amino acids and derivatives	Amino acids and peptides	Penicillin G	C16H18N2O4S	CC1(C(N2C(S1)C(C2=O)NC(=O)CC3=CC=CC=C3)C(=O)O)C	Fungus	GNPS	10.1128/aem.60.6.1705-1710.1994
1290	334,1509	12,21	333,1435		H+	333,1477	Organoheterocyclic compounds	Benzodiazepines	1,4-benzodiazepines	Amino acids and derivatives	Amino acids and peptides	Tilivalline	C20H19N3O2	C1CC2C(NC3=C(C=CC=C3O)C(=O)N2C1)C4=CNC5=CC=CC=C54	Bacteria	Npatlas	10.1016/0040-4020(82)85058-8
1291	402,2129	12,21		379,2329	Na+	379,4	Organic acids and derivatives	Carboxylic acids and derivatives	Amino acids, peptides and analogues		Amino acids and peptides	(±)-7,8-epoxy-brevianamide Q	C21H21N3O4	CC(C)(C=C)C1=C(C2=CC=CC=C2N1)C=C3C(=O)N4CC5C(C4(C(=O)N3)O)O5	Fungus (Aspergillus versicolor)	Npatlas	10.3390/md17050262

1292	303,2280	12,37		280,2480	Na+	280,4	Lipid and lipid-like molecules	Fatty acyls	Lineolic acids and derivatives		Fatty acids	Linoleic Acid	C18H32O2	CCCCCC=CCC=CCCCCCCCC(=O)O	Fungus (Aspergillus sp.)	GNPS	10.3109/13880209509065375
1294	385,2873	15,19		362,3073	Na+		Lipid and lipid-like molecules				Polyketides		C23H38O3			Sirius	
1298	742,5955	17,60	741,5881		H+		Lipid and lipid-like molecules	Fatty acyls	Sphingolipids		Fatty acids		C48H75N3O3			Sirius	
1299	654,5439	18,11	653,5365		H+		Organic acids and derivatives	Carboxylic acids and derivatives	Amino acids, peptides and analogues	Amino acids and derivatives	Fatty acids		C37H71N3O6			Sirius	
1301	336,3216	18,31	335,3142	313,3416			Lipid and lipid-like molecules	Fatty acyls	Fatty amides		N-acyl amines					Sirius	
1309	596,2280	7,27	595,2206		H+								C34H31N5O4			Sirius	
1311	307,1608	9,18	306,1534		H+	306,158	Benzenoids	Phenols	Methoxyphenols		Amino acids and peptides	3-(3-hydroxy-4-methoxybenzyl)-6-isobutyl-2,5-diketopiperazine	C16H22N2O4	CC(C)CC1C(=O)NC(C(=O)N1)CC2=CC(=C(C=C2)OC)O	Bacteria	GNPS	10.1080/14786419.2015.1137570
1313	390,1880	11,18	389,1806	367,2080													
1314	390,2230	11,52	389,2156	367,2430									C20H33NO5			Sirius	
1315	416,2382	11,90	415,2308	393,2582													
1316	388,2072	12,54	387,1998	365,2272									C20H31NO5			Sirius	
1325	395,2719	14,68	394,2645	372,2919													
1326	295,2226	14,70	294,2152		H+	294,4	Lipid and lipid-like molecules	Fatty acyls	Lineolic acids and derivatives		Fatty acids	13-keto-9Z,11E-octadecadienoic acid	C18H30O3	CCCCCC(=O)C=CC=CCCCCCCCC(=O)O	Eukaryota	GNPS	10.1021/jf801905y
1331	390,2803	18,83	389,2729	367,3003			Lipid and lipid-like molecules	Fatty acyls	Fatty acids		Fatty acids		C21H37NO4			Sirius	
1338	353,2273	12,05	352,2199		H+	352,515	Lipid and lipid-like molecules	Fatty acyls	Lineolic acids and derivatives		Fatty acids	2,3-dihydroxypropyl octadeca-9,12,15-trienoate	C21H36O4	CCC=CCC=CCC=CCCCCCCCC(=O)OCC(O)CO	Eukaryota	Sirius	
1341	333,2016	12,98		310,2216	Na+		Lipid and lipid-like molecules	Fatty acyls	Fatty acyls		Fatty acids		C18H30O4			Sirius	
1342	347,2173	13,93	346,2099	324,2373													
1362	170,1168	7,01	169,1094	147,1368	H+		Organic acids and derivatives	Carboxylic acids and derivatives					C9H15NO2			Sirius	
1363	434,1817	7,72	433,1743		H+	433,5	Organoheterocyclic compounds	Indoles and derivatives	Pyrrolindoles	Pyridoindolones	Alkaloids	Meleagrin	C23H23N5O4	CC(C)(C=C)C12C=C(C(=O)N3C1(NC(=O)C3=CC4=CN=CN4)N(C5=CC=CC=C25)OC)O	Fungus (Penicillium sp.)	GNPS	10.3923/pjbs.2014.667.674
1364	323,1038	7,94	322,0964		H+	322,3	Organic acids and derivatives	Carboxylic acids and derivatives	Amino acids, peptides, and analogues	Amino acids and derivatives	Alkaloids	6'-[(2-hydroxyphenyl)methylidene]spiro[3H-1-benzofuran-2,3'-piperazine]-2',5'-dione	C18H14N2O4	C1C2=CC=CC=C2OC13C(=O)NC(=CC4=CC=CC=C4O)C(=O)N3	Fungus (Penicillium sp.)	Sirius	10.1021/acs.orglett.6b02620
1367	390,1917	8,12	389,1843		H+	389,4	Organoheterocyclic compounds	Indoles and derivatives	Pyrrolindoles	Pyridoindolones	Alkaloids	Roquefortine C	C22H23N5O2	CC(C)(C=C)C12CC3C(=O)NC(=CC4=CN=CN4)C(=O)N3C1NC5=CC=CC=C25	Fungus (Penicillium sp.)	Sirius	10.1016/j.tet.2005.05.026

1368	136,0758	8,29	135,0684		H+	135,068	Organic oxygen compounds	Organooxygen compounds	Carboxyl compounds	Ketones	Alkaloids	2-Aminoacetophenone	C8H9NO	CC(=O)C1=CC=CC=C1N	Bacteria	Sirius	10.1007/bf00408715
1369	305,1376	8,74		282,1576	Na+	282,16	Organoheterocyclic compounds	Pyranones and derivatives	Pyrrolines		Polyketides	Cladosin A	C14H22N2O4	CC(CC(CC(=N)C1=C(C(=C(C)C)NC1=O)O)OC)O	Fungus	Sirius	10.1080/10286020.2014.940330
1372	334,1539	9,38	333,1465		H+	333,4	Organic acids and derivatives	Carboxylic acids and derivatives	Amino acids, peptides and analogues	Amino acids and derivatives	Amino acids and peptides	Isorugulosuvine (Cyclo(Trp-Phe)	C20H19N3O2	C1=CC=C(C=C1)CC2C(=O)NC(C(=O)N2)CC3=CNC4=CC=CC=C43	Fungus (Aspergillus sp.)	Sirius	10.1007/s10600-009-9433-8
1374	150,0914	9,56	149,0840		H+	149,19	Organoheterocyclic compounds	Pyridines and derivatives			Alkaloids	4-methyl-1,5,6,7-tetrahydrocyclopenta[b]pyridin-2-one	C9H11NO	CC1CC(C2=C1C=NC=C2)O	Bacteria	GNPS	10.1007/BF00567900
1375	223,0622	10,99	222,0548		H+		Organic acids and derivatives	Carboxylic acids and derivatives	Dicarboxylic acids and derivatives		Shikimates		C8H11FO6			Sirius	
1380	296,0615	14,57		273,0815	Na+	274,2	Organohalogen compounds	Aryl halides	Aryl bromides		Alkaloids	3-Bromo-2-butan-2-yl-1-(2-methoxyethyl)-5-methylpyrrole	C12H20BrNO	CCC(C)C1=C(C=C(N1CCOC)C)Br		Sirius	
1381	298,0596	14,57	297,0522		H+	298,16	Organoheterocyclic compounds	Azolidines	Oxazolidines		Alkaloids	[2-(4-Ethyl-2,2-dimethyl-1,3-oxazolidin-3-yl)-2-oxoethyl] 2,2-dichloroacetate	C11H17Cl2NO4	CCC1COC(N1C(=O)COC(=O)C(Cl)Cl)(C)C		Sirius	
1382	260,1982	14,59	259,1908	237,2182	Na+		Organic nitrogen compounds	Organonitrogen compounds			Alkaloids		C15H27NO			Sirius	
1383	382,2339	15,53		359,2539	Na+	359,5	Lipid and lipid-like molecules	Fatty acyls	Fatty amides	N-acyl amines		N-[5-[acetyl(hydroxy)amino]pentyl]-N'-hexyl-N'-hydroxybutanediamide	C17H33N3O5	CCCCCCN(C(=O)CCC(=O)NCCCCCN(C(=O)C)O)O		Sirius	
1386	396,2494	16,01		373,2694	Na+	373,5	Organoheterocyclic compounds	Piperidines	Piperidinecarboxylic acids and derivatives	Piperidinecarboxylic acids	Polyketides	Isonipecotic acid	C23H35NO3	CCCCCCCCOC(=O)C1CCN(CC1)C(=O)CCC2=CC=CC=C2		Sirius	
1388	464,3111	17,22		441,3311	Na+	441,6	Organic acids and derivatives	Carboxylic acids and derivatives	Amino acids, peptides and analogues	Amino acids and derivatives	Amino acids and peptides	Octanoyl-Gly-Ile-Leu-OMe	C23H43N3O5	CCCCCCCC(=O)NCC(=O)N[C@@H]([C@@H](C)CC)C(=O)N[C@@H](CC(C)C)C(=O)OC		Sirius	
1389	213,0750	1,43	212,0676		H+		Organic acids and derivatives	Carboxylic acids and derivatives	Amino acids, peptides and analogues	Amino acids and derivatives	Amino acids and peptides		C9H12N2O2S			Sirius	
1392	511,2855	8,72		488,3055	Na+	510,284	Organic acids and derivatives	Carboxylic acids and derivatives	Amino acids, peptides and analogues	Amino acids and derivatives	Amino acids and peptides	Phe-Val-Val-Phe	C28H38N4O5	CC(C)C(C(=O)NC(C(C)C)C(=O)NC(CC1=CC=CC=C1)C(=O)O)NC(=O)C(CC2=CC=CC=C2)N	Fungus (Penicillium sp.)	Npatlas	10.1021/acs.jafc.9b00388

1393	194,1141	9,12	193,1067		H+	193,11	Organoheterocyclic compounds	Pyridines and derivatives	Pyridine alkaloids	Pyridinecarboxylic acids	Alkaloids	Methyl fusarate	C11H15NO2	CCCCC1=CN=C(C=C1)C(=O)OC	Fungus	Sirius	10.1016/0031-9422(95)00716-4
1394	451,2129	9,36	450,2055	428,2329													
1395	362,1815	12,91		339,2015	Na+		Benzenoids	Benzene and substituted derivatives			Alkaloids		C20H25N3O2			Sirius	
1397	258,2020	13,47	257,1946		H+	235,36	Organic nitrogen compounds	Organonitrogen compounds	Amines	Secondary amines	Alkaloids	N-(1,1-difluoropropan-2-yl)decan-1-amine	C13H27F2N	CCCCCCCCCCNC(C)C(F)F		Sirius	
1402	358,2304	16,03	357,2230	335,2504													
1405	317,1093	16,60	316,1019		H+	316,094	Phenylpropanoids and polyketides	Coumarins and derivatives	Hydroxycoumarins	7-hydroxycoumarins	Shikimates and phenylpropanoids	Aspyranochromenone B	C17H16O6	CC1CC(=O)C=C(O1)C2=CC3=C(C=C(C(=C3CO)C)O)OC2=O	Fungus (Aspergillus sp.)	Npatlas	10.1016/j.tet.2020.131525
1426	121,0625	6,23		98,0825	Na+		Organic acids and derivatives	Carboxylic acids and derivatives	Amino acids, peptides and analogues	Amino acids and derivatives	Alkaloids					Sirius	
1427	210,1087	6,49		187,1287	Na+		Organic acids and derivatives	Carboxylic acids and derivatives	Amino acids, peptides and analogues	Amino acids and derivatives	Amino acids and peptides		C9H17NO3			Sirius	
1433	164,1037	8,03	163,0963		H+	163,17	Organic oxygen compounds	Organooxygen compounds	Carbohydrates and carbohydrates conjugated	Monosaccharides	Alkaloids	Mycosamine	C6H13NO4	CC(O)C(O)C(N)C(O)C=O	Bacteria	Sirius	10.1099/00221287-148-1-51
1434	105,0674	8,06	104,0600		H+	104,11	Benzenoids	Benzene and substituted derivatives	Styrenes		Shikimates and phenylpropanoids	Styrene	C8H8	C=CC1=CC=CC=C1	Fungus (Aspergillus sp.)	Npatlas	
1436	243,0833	8,44	242,0759		H+	242,23	Organoheterocyclic compounds	Pteridines and derivatives	Alloxazines and isoalloxazines	Flavins	Alkaloids	Riboflavin lumichrome	C12H10N4O2	CC1=CC2=C(C=C1C)N=C3C(=N2)C(=O)NC(=O)N3	Fungus	GNPS	10.1021/np000204t
1437	334,2898	8,59	333,2824	311,3098			Organic acids and derivatives	Boronic acid derivatives					C19H35BN2O2			Sirius	
1440	203,1141	8,74	202,1067		H+	202,1106	Organoheterocyclic compounds	Indoles and derivatives	Indoles	3-alkylindoles	Alkaloids	Nb-acetyltryptamine	C12H14NO2	CC(=O)NCCC1=CNC2=CC=CC=C21	Fungus	GNPS	10.1007/BF03179925.
1444	398,3356	9,13	397,3282	375,3556													
1445	323,1546	9,26	322,1472	300,1746													
1450	625,3826	10,29	624,3752	602,4026													
1454	317,1301	11,83		294,1501	Na+	294,34	Organoheterocyclic compounds	Coumarins and derivatives	Isocoumarins	Dihydroisocoumarines	polyketides	Aspergimarin D	C16H22O5	CC(CCCC1CC2=C(C(=C(C=C2)OC)OC)C(=O)O1)O	Fungus (Aspergillus sp.)	Npatlas	10.3389/fmicb.2019.02846
1456	267,1670	13,33		244,1870	Na+	244,33	Lipid and lipid-like molecules	Fatty acyls	Fatty acids and conjugated	Medium-chain fatty acids	Fatty acids	6-(6-aminohexylamino)-6-oxohexanoic acid	C12H24N2O3	C(CCCNC(=O)CCCCC(=O)O)CCN		Sirius	
1457	502,3662	14,56	501,3588		H+		Lipid and lipid-like molecules	Fatty acyls	Fatty amides		Fatty acids		C23H43N5O7			Sirius	
1458	458,3413	14,63		435,3613	Na+		Lipid and lipid-like molecules	Fatty acyls	Fatty acyls		Fatty acids		C23H49NO6			Sirius	

1460	482,3983	15,71	481,3909		H+		Organic acids and derivatives	Carboxylic acids and derivatives	Amino acids, peptides and analogues	Amino acids and derivatives	Amino acids and peptides		C27H51N3O4			Sirius	
1461	756,6097	18,38	755,6023		H+		Organic acids and derivatives	Peptidomimetics	Depsipeptides		Oligopeptides		C42H81N3O8			Sirius	
1462	610,5173	18,51	609,5099		H+		Organic acids and derivatives	Carboxylic acids and derivatives	Amino acids, peptides and analogues	Amino acids and derivatives	Amino acids and peptides		C35H67N3O5			Sirius	
1463	712,5845	18,59	711,5771		H+		Organic acids and derivatives	Carboxylic acids and derivatives	Amino acids, peptides and analogues	Amino acids and derivatives	Fatty amides		C43H75N4O4			Sirius	
1467	668,5583	18,91	667,5509		H+		Organic acids and derivatives	Peptidomimetics	Depsipeptides		Oligopeptides		C38H73N3O6			Sirius	
1468	770,6255	19,00		747,6455	Na+		Lipid and lipid-like molecules		Triterpenoids		Terpenoids		C44H77NO8			Sirius	
1470	173,0676	6,47		150,0876	Na+	150,079	Benzenoids	Benzene and substituted derivatives	Benzoic acids and derivatives	Aminobenzoic acids and derivatives	Amino acids and peptides	4-(Methylamino)benzamide	C5H8N4O3	CNC1=CC=C(C=C1)C(=O)N	Bacteria	Sirius	10.1021/acs.jnatprod.6b00948
1472	538,1781	7,92	537,1707	515,1981													
1474	618,2840	8,71	617,2766		H+								C35H43N3O7			Sirius	
1477	420,1604	11,14	419,1530		H+	419,4	Organic acids and derivatives	Carboxylic acids and derivatives	Amino acids, peptides and analogues	Alpha amino acids and derivatives	pokyketides	Sorbicillactone B	C21H25NO8	CC=CCCC(=O)C1=C(C(=C(C2(C1C(C(=O)O2)(C)NC(=O)C=CC(=O)O)C)O)C)O	Fungus (Penicillium sp.)	NpAtlas	10.1016/j.tet.2005.05.026
1487	191,0811	6,47	190,0737		H+	190,07	Organoheterocyclic compounds	Diazanaphthalenes	Benzodiazines	Quinazolines	Alkaloids	2-(1-hydroxyethyl)-4(3H)quinazoline	C8H12N2O2	CC(C1=NC2=CC=CC=C2C(=O)N1)O	Fungus	Sirius	10.1139/v93-176
1488	211,1436	6,60	210,1362		H+	210,13	Organic acids and derivatives	Carboxylic acids and derivatives	Amino acids, peptides, and analogues	Amino acids and derivatives	Amino acids and peptides	Cyclo(Leu-Pro)	C11H18N2O2	CC(C)CC1C(=O)N2CCCC2C(=O)N1	Bacteria	GNPS	10.1021/np030233e
1489	596,2341	7,27	595,2267		H+								C31H45N7O5			Sirius	
1493	495,2027	10,49	494,1953		H+	494,5	Organic acids and derivatives	Carboxylic acids and derivatives	Amino acids, peptides and analogues	Amino acids and derivatives	Amino acids and peptides	2-[(4E)-4-[(1-benzyl-2-methylindol-3-yl)methylidene]-2,5-dioxoimidazolidin-1-yl]-N-(2-methoxyphenyl)acetamide	C29H26N4O4	CC1=C(C2=CC=CC=C2N1CC3=CC=CC=C3)/C=C/4\C(=O)N(C(=O)N4)CC(=O)NC5=CC=CC=C5OC		Sirius	
1494	513,2132	10,49		490,2332	Na+	490,5	Benzenoids	Benzene and substituted derivatives	Phenethylamines		Amino acids and peptides	N~3~-[(2S)-1-hydroxy-3-phenylpropan-2-yl]-N~2~-(2-{[(2S)-1-hydroxy-3-phenylpropan-2-yl]amino}-2-oxoethyl)pyridine-2,3-dicarboxamide	C27H30N4O5	C1=CC=C(C=C1)CC(CO)NC(=O)CNC(=O)C2=C(C=CC=N2)C(=O)NC(CC3=CC=CC=C3)CO		Sirius	
1496	657,1807	11,64		634,2007	Na+	634,63	Lipid and lipid-like molecules	Prenol lipids	Diterpenoids		Polyketides	2-Anthracene butanoic acid	C37H30O10	C1=CC=C2C=C3C=C(C=CC3=CC2=C1)CCCC(=O)O		Sirius	

1497	212,1064	11,76	211,0990		H+	211,084	Benzenoids	Benzene and substituted derivatives	Anilides	Acetanilides	Alkaloids	2,5-Dihydroxy-4-methoxymethyl-acetanilide	C14H13NO	CC(=O)NC1=C(C=C(C(=C1)O)COC)O	Bacteria	Sirius	10.7164/antibiotics.55.635
1498	501,2478	12,01	500,2404		H+	500,588	Lipid and lipid-like molecules	Steroids and steroid derivatives	Steroids esters		Terpenoids	Citreohybridonol	C28H36O8	CC1=CC2C(CC3C4C2(CCC(C4(C)C)OC(=O)C)C(=O)O3)(C5(C1(C(=C(C5=O)C)O)C)C(=O)OC)C	Fungus (Penicillium sp.)	GNPS	10.1016/s0040-4039(00)74810-6
1504	217,0645	1,07		194,0845	Na+	194,09	Organoheterocyclic compounds	Benzopyrans	1-benzopyrans		Polyketides	5-methoxy-2-methyl-3,4-dihydro-2h-1-benzopyran-4-ol	C11H14O3	CC1CC(C2=C(O1)C=CC=C2OC)O	Fungus (Penicillium sp.)	Npatlas	10.1080/14786419.2015.1072712
1520	320,1671	6,01		297,1871	Na+	297,157	Organic oxygen compounds	Organooxygen compounds	Carbonyl compounds	Ketones	Polyketides	Atpenin B	C15H23NO5	CCC(C)CC(C)C(=O)C1=C(C(=C(NC1=O)OC)OC)O	Fungus (Penicillium sp.)	Npatlas	10.7164/antibiotics.41.1769
1525	511,2483	9,84	510,2409		H+	510,5	Organoheterocyclic compounds	Indoles and derivatives	Indoles	beta carboline	Alkaloids	6-hydroxymetatacarboline G	C324H30N4O7	CCC(C)C(C(=O)O)NC(=O)C1=NC(=C2C(=C1)C3=C(N2)C=CC(=C3)O)C)CC(=O)N4CCCC4C(=O)O	Fungus	GNPS	10.1021/np300455a
1526	241,0931	10,00	240,0857	218,1131	Na+	218,25	Organoheterocyclic compounds	Diazanaphtalenes	Benzodiazines	Quinoxalines	Alkaloids	(2R)-3-(3-methylquinoxalin-2-yl)propane-1,2-diol	C12H14N2O2	CC1=NC2=CC=CC=C2N=C1CC(CO)O		Sirius	
1527	283,1033	10,01	282,0959		H+	282,25	Organoheterocyclic compounds	Imidazopyrimidines	Purines and purines derivatives	Xanthines	Alkaloids	2-[3-(2-methoxyethyl)-1-methyl-2,6-dioxo-7H-purin-8-yl]acetic acid	C11H14N4O5	CN1C(=O)C2=C(N=C(N2)CC(=O)O)N(C1=O)CCOC		Sirius	
1532	333,1995	13,25		310,2195	Na+		Lipid and lipid-like molecules	Fatty acyls	Fatty acyls		Fatty acids		C18H30O4			Sirius	
1533	313,2330	13,89	312,2256		H+		Organic nitrogen compounds	Organonitrogen compounds	Amines		Polyamines		C9H28N8O4			Sirius	
1546	360,3566	12,57	359,3492		H+		Organic acids and derivatives	Organonitrogen compounds	Polyamines		Fatty acids		C20H45N3O2			Sirius	
1550	388,3877	13,40	387,3803		H+		Organic acids and derivatives	Organonitrogen compounds	Polyamines		Fatty acids		C22H49N3O2			Sirius	
1551	459,2140	13,75	458,2066	436,2340													
1557	430,1949	9,31	429,1875	407,2149			Organic acids and derivatives	Carboxylic acids and derivatives	Amino acids, peptides and analogues	Amino acids and derivatives	Amino acids and peptides		C23H25N3O4			Sirius	
1558	522,1837	9,58	521,1763	499,2037			Organic acids and derivatives	Carboxylic acids and derivatives	Amino acids, peptides and analogues	Amino acids and derivatives	Amino acids and peptides		C26H27N5O7			Sirius	
1560	243,1700	12,31		220,1900	Na+		Lipid and lipid-like molecules	Prenol lipids	Sesquiterpenoids		Terpenoids		C15H24O			GNPS	

1561	261,1804	12,31		238,2004	Na+	238,193	Lipid and lipid-like molecules	Prenol lipids	Sesquiterpenoids		Terpenoids	Penicieudesmol A	C15H26O2	CC1CC(C(C2(C1CC(CC2)C(=C)C)C)O)O	Fungus (Penicillium sp.)	Sirius	10.3390/md16040108
1562	415,2417	12,41	414,2343	392,2617													
1563	372,3415	12,44	371,3341	349,3615													
1567	214,2124	14,13	213,2050	191,2324									C8H23B2N3O2			Sirius	
1573	229,0970	5,93		206,1170	Na+	206,09	Organoheterocyclic compounds	Benzopyrans			Polyketides	8-methoxy-3,5-dimethyl-3,4-dihydroisochromen-6-one	C12H14O3	CC1CC2=C(C(=O)C=C(C2=CO1)OC)C	Fungus (Penicillium sp.)	Npatlas	10.1021/np0498859
1575	227,1716	8,59	226,1642		H+	226,168	Organic acids and derivatives	Carboxylic acids and derivatives	Amino acids, peptides and analogues	Amino acids and derivatives	Amino acids and peptides	Cyclo(Leu-Ile)	C12H22N2O2	CCC(C)C1C(=O)NC(C(=O)N1)CC(C)C	Fungus	Npatlas	10.1016/s0031-9422(01)00470-8
1580	366,1705	1,08	365,1631		H+		Organic acids and derivatives	Carboxylic acids and derivatives	Amino acids, peptides and analogues	Amino acids and derivatives	Amino acids and peptides		C16H23N5O5			Sirius	
1597	308,2173	7,33		285,2373	Na+		Organic acids and derivatives	Carboxylic acids and derivatives	Amino acids, peptides and analogues	Amino acids and derivatives	Amino acids and peptides		C16H31NO3			Sirius	
1602	202,1398	8,74	201,1324		H+		Organic acids and derivatives	Carboxylic acids and derivatives	Amino acids, peptides and analogues	Amino acids and derivatives	Amino acids and peptides					Sirius	
1604	180,0621	8,87	179,0547		H+	157,07	Organic acids and derivatives	Carboxylic acids and derivatives	Amino acids, peptides and analogues	Amino acids and derivatives	Amino acids and peptides	N-Acetyl-hydroxyvaline lactone	C7H11NO3	CC1COC(=O)C1NC(=O)C	Bacteria	Npatlas	10.1021/np990507r
1606	120,0419	8,89		97,0619	Na+	97,1154	Organoheterocyclic compounds	Pyridines and derivatives	Hydropyridines		Alkaloids	5,6-Dihydro-2(1H)-pyridinone	C5H7NO	OC1=NCCC=C1	Eukaryota	Sirius	
1609	233,1131	9,54		210,1331	Na+	210,27	Phenylpropanoids and polyketides	Macrolides and analogues			Monoterpenoids	Patulolide B	C12H18O3	CC1CCCCCCC(=O)C=CC(=O)O1	Fungus (Penicillium sp.)	Sirius	10.7164/antibiotics.41.1649
1610	287,0843	9,80	286,0769		H+	286,2783	Organic oxygen compounds	Organooxygen compounds	Carbohydrates and carbohydrate conjugated	Glycosyl compounds	Shikimates and Phenylpropanoids	salicin	C13H18O7	C1=CC=C(C(=C1)CO)OC2C(C(C(C(O2)CO)O)O)O	Eukaryota	GNPS	10.1021/np0505216
1612	305,1338	10,40		282,1538	Na+		Phenylpropanoids and polyketides	Macrolides and analogues			Polyketides		C16H16O6			Sirius	
1613	221,1136	12,28		198,1336	Na+	198,13	Lipid and lipid-like molecules	Fatty acyls	Fatty alcohols		Polyketides	Streptenol F	C11H18O3	CC=CC=CC(=O)CC(CCO)OC	Bacteria	Npatlas	10.1021/acs.jnatprod.6b01057
1614	219,0981	12,44		196,1181	Na+	196,11	Organoheterocyclic compounds	Pyrans	Pyranones and derivatives		Polyketides	10-hydroxymucidone	C11H16O3	CCC1=CC=C(CC(C)(C)O)C(=O)O1	Bacteria	Npatlas	10.1021/acs.jnatprod.6b00175
1615	235,1292	12,67		212,1492	Na+	234,29	Lipid and lipid-like molecules	Prenol lipids	Monoterpenoids	Acyclic monoterpenoids	Terpenoids	5,9-dimethyl-10-oxododeca-2,4,6,8-tetraenoic acid	C14H18O3	CCC(=O)/C(=C\C=C\C(=C/C=C/C(=O)O)\C)/C	Bacteria	Npatlas	10.1007/s10600-014-0970-4
1616	292,0786	14,29		269,0986	Na+		Benzenoids	Benzene and substituted derivatives	Phenoxyacetic acid and derivatives				C12H15NO6			Sirius	

1617	306,2387	14,69		283,2587	Na+		Lipid and lipid-like molecules	Fatty acyls	Fatty amides		Fatty acids		$C_{17}H_{33}NO_2$			Sirius	
1621	375,3417	17,71		352,3617	Na+		Lipid and lipid-like molecules	Fatty acyls	Unsatured fatty acids		Fatty acids		$C_{22}H_{40}O_3$			Sirius	
1625	433,3829	18,07		410,4029	Na+		Lipid and lipid-like molecules		Triterpenoids	Acyclic triterpenoids	Terpenoids		$C_{30}H_{50}$			Sirius	
1626	450,4092	18,07		427,4292	Na+		Lipid and lipid-like molecules	Fatty acyls	Fatty esters		Fatty acids		$C_{25}H_{49}NO_4$			Sirius	
1627	455,3645	18,07		432,3845	Na+		Lipid and lipid-like molecules	Fatty acyls					$C_{32}H_{48}$			Sirius	

row ID	row m/z	RT	M-H+	M-Na+	Adduct	Exact Mass	SuperClass	Class	Subclass	Level 5	Putative annotation	Chemical formula	Smiles	Organism	Activity	DOI activity
25	191,0673	2,03	190,0599		H+	190,063	Phenylpropanoids and polyketides	Coumarins and derivatives	Isocoumarins		7-hydroxy-3, 5-dimethyl-isochromen-1-one	C11H10O3	CC1=CC2=C(C=C(C=C2C(=O)O1)O)C	Fungus	Antifungal	10.1016/j.fitote.2011.10.013
33	261,1200	5,75	260,1126		H+	260,29	Organic acids and derivatives	Carboxylic acids and derivatives	Amino acids, peptides, and analogues	Amino acids and derivatives	Cyclo(Pro-Tyr)	C14H16N2O3	C1CC2C(=O)NC(C(=O)N2C1)CC3=CC=C(C=C3)O	Fungus	AntiQS	10.1016/j.isci.2023.107048.
34	211,1410	6,88	210,1336		H+	210,13	Organic acids and derivatives	Carboxylic acids and derivatives	Amino acids, peptides, and analogues	Amino acids and derivatives	Cyclo(Leu-Pro)	C11H18N2O2	CC(C)CC1C(=O)N2CCCC2C(=O)N1	Bacteria	AntiQS (diketopiperazines)	10.4014/jmb.1907.07030
36	245,1251	7,46	244,1177		H+	244,29	Organic acids and derivatives	Carboxylic acids and derivatives	Amino acids, peptides, and analogues	Amino acids and derivatives	Cyclo(Phe-Pro)	C14H16N2O2	C1CC2C(=O)NC(C(=O)N2C1)CC3=CC=CC=C3	Bacteria	AntiQS	10.1128/mBio.00366-13
38	261,1566	8,89	260,1492		H+	260,33	Organic acids and derivatives	Carboxylic acids and derivatives	Amino acids, peptides, and analogues	Amino acids and derivatives	Cyclo(Phe-Leu)	C15H20N2O2	CC(C)CC1C(=O)NC(C(=O)N1)CC2=CC=CC=C2	Fungus	Antimicrobial	10.3390/polym14214554
76	610,1783	19,15		587,1983	Na+	587,23	Phenylpropanoids and polyketides	Anthraquinones and anthrones			Nothramicin	C30H37NO11	CC1C(C(CC(O1)OC2C(C(C(C3=CC4=C(C(=C23)O)C(=O)C5=C(C=CC(=C5C4=O)OC)O)O)(C)O)OC)(C)N(C)C)O	Bacteria	Antibacterial	10.7164/antibiotics.51.130
107	227,1358	5,70	226,1284		H+	226,27	Organic acids and derivatives	Carboxylic acids and derivatives	Amino acids, peptides, and analogues	Amino acids and derivatives	Cyclo(Leu-trans-4-hydroxy-Pro)	C11H18N2O3	CC(C)CC1C(=O)N2CC(CC2C(=O)N1)O	Fungus	Antimicrobial (Algicide)	10.1038/srep14720
109	213,1564	7,71	212,1490		H+	212,15	Organic acids and derivatives	Carboxylic acids and derivatives	Amino acids, peptides, and analogues	Amino acids and derivatives	Cyclo(Val-Leu)	C11H20N2O2	CC(C)CC1C(=O)NC(C(=O)N1)C(C)C	Fungus	Probiotic	10.1038/s41598-020-64374-w.
112	558,3223	9,87		535,3423	Na+	535,328	Organoheterocyclic compounds	Prenol lipids	Diterpenoids		Drechmerin C	C33H45NO5	CC(=CCOC(C)(C)C1CC(C2(C3CCC4CC5=C(C4(C3(CCC2O1)C)C)NC6=CC=CC=C56)C(=O)O)O)C	Fungus	Antimicrobial	10.1016/j.phytochem.2018.01.010
114	556,3636	11,30	555,3562		H+	555,31	Organoheterocyclic compounds	Prenol lipids	Diterpenoids		Drechmerin F	C32H45NO7	CC12CCC3C4(C1(CCC5C2(C6=C(C5)C7=C(C=CC=C7N6)CC(C(C)(C)O)O)C)O)C(O4)C(C(O3)C(C)(C)O)O	Fungus	Antimicrobial	10.1016/j.phytochem.2018.01.010
163	160,0730	1,41	159,0656		H+	159,068	Organoheterocyclic compounds	Indoles and derivatives	Indoles		2H-Indol-2-one	C10H9NO3	C/C=C\1/C2=CC=CC=C2NC1=O	Fungus	Antimicrobial	10.1007/bf02040097
167	235,1042	4,46	234,0968		H+	234,0892	Benzenoids	Benzene and substituted derivatives	Benzoic acids and derivatives	Hydroxybenzoic acid and derivatives	5,7-dihydroxy-6-(3-methylbut-2-enyl)isobenzofuran-1(3H)-one	C13H14O4	CC(=CCC1=C(C=C2COC(=O)C2=C1O)O)C	Fungus	Antimicrobial (isobenzofuranone)	10.1016/j.phytochem.2014.04.006
168	169,0731	5,39	168,0657		H+	168,195	Organoheterocyclic compounds	Indoles and derivatives	Carbolin alkaloids	β-carboline	β-carboline	C11H8N2	C1=CC=C2C(=C1)C3=C(N2)C=NC=C3	Bacteria	AntiQS	10.1080/08927014.2021.1874942
171	311,1349	7,95	310,1275		H+	310,3478	Organic acids and derivatives	Carboxylic acids and derivatives	Amino acids, peptides and analogues	Amino acids and derivatives	Cyclo(Tyr-Phe)	C18H18N2O3	C1=CC=C(C=C1)CC2C(=O)NC(C(=O)N2)CC3=CC=C(C=C3)O	Bacteria	Cyclopeptides	10.3390/biom10020298
172	227,1722	8,74	226,1648		H+	226,168	Organic acids and derivatives	Carboxylic acids and derivatives	Amino acids, peptides, and analogues	Amino acids and derivatives	Cyclo(Leu-Ile)	C12H22N2O2	CCC(C)C1C(=O)NC(C(=O)N1)CC(C)C	Fungus	Antifungal	10.1016/S1875-5364(12)60016-8
186	536,1593	18,65	535,1519		H+	535,147	Benzenoids	Anthraquinones and anthrones	Anthraquinones		Actinoplanone D	C28H25NO10	CC1=CC2=CC3=C(C4=C5C(C3)OCOC5=C6C(=C4O)C(=O)C7=C(O6)C(C(CC7OC)O)OC)C(=C2C(=O)N1)O	Bacteria	Antimicrobien	10.7164/antibiotics.41.741
192	247,1404	8,22		224,1604	Na+	224,152	Organoheterocyclic compounds	Diazines	Pyrazines		Aspergillic acid	C12H20N2O2	CCC(C)C1=CN=C(C(=O)N1O)CC(C)C	Fungus	Antibacterial	10.1016/S0021-9258(19)77398-0
218	289,0820	11,18		266,1020	Na+	266,0943	Organoheterocyclic compounds	Dihydrofurans	Furanones	Butenolides	Microperfuranone	C17H14O3	C1=CC=C(C=C1)CC2=C(C(=O)OC2O)C3=CC=CC=C3	Fungus	AntiQS	10.1007/978-3-319-14836-6_1
231	197,1255	5,21	196,1181		H+	196,12	Organic acids and derivatives	Carboxylic acids and derivatives	Amino acids, peptides, and analogues	Amino acids and derivatives	Cyclo(Pro-Val)	C10H16N2O2	C1CC(N(C1)CC(CO)N)CO	Fungus	AntiQS	10.3390/biom10020298
232	227,1355	5,70	226,1281		H+	226,27	Organic acids and derivatives	Carboxylic acids and derivatives	Amino acids, peptides, and analogues	Amino acids and derivatives	Cyclo(Leu-trans-4-hydroxy-Pro)	C11H18N2O3	CC(C)CC1C(=O)N2CC(CC2C(=O)N1)O	Fungus	Antimicrobial (Algicide)	10.1038/srep14720
233	261,1195	5,75	260,1121		H+	260,29	Organic acids and derivatives	Carboxylic acids and derivatives	Amino acids, peptides, and analogues	Amino acids and derivatives	Cyclo(Pro-Tyr)	C14H16N2O3	C1CC2C(=O)NC(C(=O)N2C1)CC3=CC=C(C=C3)O	Fungus	AntiQS	10.1016/j.isci.2023.107048.
234	185,1252	5,95	184,1178		H+	184,12	Organic acids and derivatives	Carboxylic acids and derivatives	Amino acids, peptides, and analogues	Amino acids and derivatives	Cyclo(Ala-Ile)	C9H16N2O2	CCC(C)C1C(=O)NC(C(=O)N1)C	Fungus	Antimicrobial (Antiviral)	10.1016/j.jep.2018.01.005
236	211,1405	6,88	210,1331		H+	210,13	Organic acids and derivatives	Carboxylic acids and derivatives	Amino acids, peptides, and analogues	Amino acids and derivatives	Cyclo(Leu-Pro)	C11H18N2O2	CC(C)CC1C(=O)N2CCCC2C(=O)N1	Bacteria	AntiQS (diketopiperazines)	10.4014/jmb.1907.07030
237	342,1977	7,47	341,1903		H+	341,17	Organic acids and derivatives	Carboxylic acids and derivatives	Amino acids, peptides, and analogues		14-hydroxyterezine D	C19H23N3O3	CC1C(=O)N(C(C(=O)N1)CC2=CNC3=C(C=CC=C23)CC=C(C)C)O	Fungus	Antimicrobial	10.1590/1414-431X2023e12404
245	282,2176	16,84		259,2376	Na+	259,437	Organoheterocyclic compounds	Quinolines and derivatives	Hydroquinolines		1,2,3,4-tétrahydro-5-nonylquinoline	C18H29N	CCCCCCCCCC1=C2CCCNC2=CC=C1	Bacteria	Antifungal	10.1021/acs.orglett.5b00607
280	197,1246	5,20	196,1172		H+	196,12	Organic acids and derivatives	Carboxylic acids and derivatives	Amino acids, peptides, and analogues	Amino acids and derivatives	Cyclo(Pro-Val)	C10H16N2O2	C1CC(N(C1)CC(CO)N)CO	Fungus	AntiQS	10.3390/biom10020298
281	227,1347	5,70	226,1273		H+	226,27	Organic acids and derivatives	Carboxylic acids and derivatives	Amino acids, peptides, and analogues	Amino acids and derivatives	Cyclo(Leu-trans-4-hydroxy-Pro)	C11H18N2O3	CC(C)CC1C(=O)N2CC(CC2C(=O)N1)O	Fungus	Antimicrobial (Algicide)	10.1038/srep14720
282	261,1188	5,75	260,1114		H+	260,29	Organic acids and derivatives	Carboxylic acids and derivatives	Amino acids, peptides, and analogues	Amino acids and derivatives	Cyclo(Pro-Tyr)	C14H16N2O3	C1CC2C(=O)NC(C(=O)N2C1)CC3=CC=C(C=C3)O	Fungus	AntiQS	10.1016/j.isci.2023.107048.
283	185,1247	5,95	184,1173		H+	184,12	Organic acids and derivatives	Carboxylic acids and derivatives	Amino acids, peptides, and analogues	Amino acids and derivatives	Cyclo(Ala-Ile)	C9H16N2O2	CCC(C)C1C(=O)NC(C(=O)N1)C	Fungus	Antimicrobial (Antiviral)	10.1016/j.jep.2018.01.005
286	227,1716	8,74	226,1642		H+	226,168	Organic acids and derivatives	Carboxylic acids and derivatives	Amino acids, peptides, and analogues	Amino acids and derivatives	Cyclo(Leu-Ile)	C12H22N2O2	CCC(C)C1C(=O)NC(C(=O)N1)CC(C)C	Fungus	Antifungal	10.1016/S1875-5364(12)60016-8
310	536,1581	18,64	535,1507		H+	535,147	Organoheterocyclic compounds	Benzopyrans	1-benzopyrans	Xanthenes	Simaomicin	C28H25NO10	CC1=CC2=C(C(=C3C4=C2OCOC4CC5=C3C(=C6C(=C5OC)OC7=C(C6=O)C(CCC7O)O)O)O)C(=O)N1C	Bacteria	Antibacterial	10.7164/antibiotics.43.1059
347	197,1276	5,20	196,1202		H+	196,12	Organic acids and derivatives	Carboxylic acids and derivatives	Amino acids, peptides, and analogues	Amino acids and derivatives	Cyclo(Pro-Val)	C10H16N2O2	C1CC(N(C1)CC(CO)N)CO	Fungus	AntiQS	10.3390/biom10020298
349	261,1225	5,74	260,1151		H+	260,29	Organic acids and derivatives	Carboxylic acids and derivatives	Amino acids, peptides, and analogues	Amino acids and derivatives	Cyclo(Pro-Tyr)	C14H16N2O3	C1CC2C(=O)NC(C(=O)N2C1)CC3=CC=C(C=C3)O	Fungus	AntiQS	10.1016/j.isci.2023.107048.
350	185,1278	5,94	184,1204		H+	184,12	Organic acids and derivatives	Carboxylic acids and derivatives	Amino acids, peptides, and analogues	Amino acids and derivatives	Cyclo(Ala-Ile)	C9H16N2O2	CCC(C)C1C(=O)NC(C(=O)N1)C	Fungus	Antimicrobial (Antiviral)	10.1016/j.jep.2018.01.005
352	211,1432	6,88	210,1358		H+	210,13	Organic acids and derivatives	Carboxylic acids and derivatives	Amino acids, peptides, and analogues	Amino acids and derivatives	Cyclo(Leu-Pro)	C11H18N2O2	CC(C)CC1C(=O)N2CCCC2C(=O)N1	Bacteria	AntiQS (diketopiperazines)	10.4014/jmb.1907.07030
353	287,0512	12,39		264,0712	Na+	264,233	Phenylpropanoids and polyketides	Coumarins and derivatives			isocoumarin NM-3	C13H12O6	CC(C1=CC2=CC(=CC(=C2C(=O)O1)O)OC)C(=O)O	Bacteria	Coumarin	10.1007/s00253-018-8787-x.
383	169,0752	5,37	168,0678		H+	168,2	Organoheterocyclic compounds	Indoles and derivatives	Carbolin alkaloids	β-carboline	β-carboline	C11H8N2	C1=CC=C2C(=C1)C3=C(N2)C=NC=C3	Bacteria	AntiQS	10.1080/08927014.2021.1874942
384	245,1277	7,46	244,1203		H+	244,29	Organic acids and derivatives	Carboxylic acids and derivatives	Amino acids, peptides, and analogues	Amino acids and derivatives	Cyclo(Phe-Pro)	C14H16N2O2	C1CC2C(=O)NC(C(=O)N2C1)CC3=CC=CC=C3	Bacteria	AntiQS	10.1128/mBio.00366-13
385	213,1591	7,71	212,1517		H+	212,15	Organic acids and derivatives	Carboxylic acids and derivatives	Amino acids, peptides, and analogues	Amino acids and derivatives	Cyclo(Val-Leu)	C11H20N2O2	CC(C)CC1C(=O)NC(C(=O)N1)C(C)C	Fungus	Probiotic	10.1038/s41598-020-64374-w.
386	311,1382	7,94	310,1308		H+	310,3478	Organic acids and derivatives	Carboxylic acids and derivatives	Amino acids, peptides and analogues	Amino acids and derivatives	Cyclo(Tyr-Phe)	C18H18N2O3	C1=CC=C(C=C1)CC2C(=O)NC(C(=O)N2)CC3=CC=C(C=C3)O	Bacteria	Cyclopeptides	10.3390/biom10020298
387	247,1433	8,22	246,1359		H+	246,3	Organic acids and derivatives	Carboxylic acids and derivatives	Amino acids, peptides, and analogues	Amino acids and derivatives	Cyclo(Phe-Val)	C14H18N2O2	CC(C)C1C(=O)NC(C(=O)N1)CC2=CC=CC=C2	Fungus	AntiQS	10.1021/acsomega.2c02202
399	385,1793	14,90	384,1719		H+	384,1937	Lipid and lipid-like molecules	Fatty acyls	Fatty alcohols	Azaphilones	9-Decanoyl-3,6a-dimethylfuro[2,3-h]isochromene-6,8-dione	C23H28O5	CCCCCCCCCC(=O)C1=C2C3=COC(=CC3=CC(=O)C2(OC1=O)C)C	Fungus	Antibacterial	10.1021/acs.jnatprod.5b00436
435	300,1665	8,79	299,1591		H+	299,15	Organoheterocyclic compounds	Indoles and derivatives	Carbobazoles	Amino acids and derivatives	Streptoverticillin	C18H21NO3	CC1=C(C2=C(C3=CC=CC=C3N2)C(=C1OC)OC)C[C@@H](C)O	Bacteria	Antifungal	10.1038/ja.2007.19
439	287,0510	11,82		264,0710	Na+	264,23	Organoheterocyclic compounds	Benzopyrans	2-benzopyrans		6-hydroxy-8-methoxy-3a-methyl-3a,9b-dihydro-3h-furo[3,2-c]isochromene-2,5-dione	C13H12O6	CC12CC(=O)OC1C3=C(C(=CC(=C3)OC)O)C(=O)O2	Fungus	Antimicrobial	10.1016/j.fitote.2011.10.013
455	211,1405	7,09	210,1331		H+	210,13	Organic acids and derivatives	Carboxylic acids and derivatives	Amino acids, peptides, and analogues	Amino acids and derivatives	Cyclo(Leu-Pro)	C11H18N2O2	CC(C)CC1C(=O)N2CCCC2C(=O)N1	Bacteria	AntiQS (diketopiperazines)	10.4014/jmb.1907.07030
486	191,0780	6,47	190,0706		H+	190,07	Organoheterocyclic compounds	Diazanaphtalenes	Benzodiazines	Quinazolines	2-(1-hydroxyethyl)-4(3H)quinazoline	C8H12N2O2	CC(C1=NC2=CC=CC=C2C(=O)N1)O	Fungus	Quinazoline antiQS	10.1016/j.bioorg.2022.106226.
499	301,0663	8,35		278,0863	Na+	278,26	Phenylpropanoids and polyketides	Coumarins and derivatives			2-(5,7-dimethoxy-4-methyl-2-oxochromen-3-yl)acetic acid	C14H14O6	CC1=C(C(=O)OC2=C1C(=CC(=C2)OC)OC)CC(=O)O		Coumarin	10.1007/s00253-018-8787-x.
519	342,1972	7,28	341,1898		H+	341,17	Organic acids and derivatives	Carboxylic acids and derivatives	Amino acids, peptides, and analogues		14-hydroxyterezine D	C19H23N3O3	CC1C(=O)N(C(C(=O)N1)CC2=CNC3=C(C=CC=C23)CC=C(C)C)O	Fungus	Antimicrobial	10.1590/1414-431X2023e12404
559	305,1336	8,75		282,1536	Na+	282,158	Organoheterocyclic compounds	Pyranones and derivatives	Pyrrolines		Cladosin A	C14H22N2O4	CC(CC(CC(=N)C1=C(C(=C(C)C)NC1=O)O)OC)O	Fungus	Antimicrobial (Antiviral)	10.1021/np400833x
585	180,0987	6,23	179,0913		H+	179,0946	Organoheterocyclic compounds	Pyridines and derivatives	Pyridine alkaloids	Pyridinecarboxylic acids	Fusaric acid	C10H13NO2	CCCCC1=CN=C(C(=O)O)C=C1	Fungus	AntiQS	10.1016/j.ejmech.2016.11.044
608	433,1083	8,53	432,1009		H+	432,1056	Organoheterocyclic compounds	Azaphilones	Chromanes		Purpurquinone B	C21H20O10	CC=CC1=CC2=CC(=O)C(C(=O)C2(C(O1)O)O)(C)OC(=O)C3=C(C(=C(C=C3O)O)O)C	Fungus	Antimicrobial (Antiviral)	10.1016/j.antiviral.2017.04.007

704	572,3372	10,42	571,3298		H+	571,79	Organoheterocyclic compounds	Naphthopyrans	Diterpenoids		21,22-Diprenylpaxilline	C37H49NO4	CC(=CCC1=CC2=C(C=C1CC=C(C)C)NC3=C2CC4C3(C5(CCC6C(=CC(=O)C(O6)C(C)(C)O)C5(C C4)O)C)C)C	Fungus	Antimicrobial (Antiviral)	10.1021/np400304q
713	355,2799	14,48		332,2999	Na+	332,4	Lipids and lipid-like molecules	Prenol lipids	Diterpenoids	Gibberellins	Gibberellin A12	C20H28O4	CC12CCCC(C1C(C34C2CCC(C3)C(=C)C4)C(=O)O)(C)C(=O)O	Fungus	Plant signaling	10.1242/dev.087650.
717	215,0127	1,48		192,0327	Na+	214,3	Organic nitrogen compounds	Organonitrogen compounds	N-arylamides	N-acetylarylamines	Holomycin	C7H6N2O2S2	CC(=O)NC1=C2C(=CSS2)NC1=O	Bacteria	Antibacterial	10.1128/AEM.00169-21
741	375,0989	5,69		352,1189	Na+	352,115	Organoheterocyclic compounds	Naphthopyrans	Naphthopyranones		O-ethylhydroxydihydrofusarubin	C17H20O8	CCOC1(CC2C(=O)C3=C(C(=CC(=C3O)OC)O)C(=O)C2(CO1)O)C	Fungus	Antibacterial	10.7164/antibiotics.32.685
744	357,0886	6,59		334,1086	Na+	334,105	Benzenoids	Anthraquinones and anthrones	Anthraquinones	Hydroxyanthraquinones	Aspetritone B	C17H18O7	CC1(CC2=C(CC1O)C(=O)C3=CC(=C(C(=C3C2=O)O)OC)OC)O	Fungus	Antibacterial	10.3390/md15110348
834	197,1507	12,15		174,1707	Na+	174,28	Lipids and lipid-like molecules	Fatty acyls	Fatty alcohols		2,6-dimethyloctane-1,8-diol	C10H22O2	CC(CO)CCCC(C)CCO	Eukaryota	Antibacterial	10.1080/14786419.2022.2092735
885	503,2580	11,50	502,2506		H+	502,25	Lipid and lipid-like molecules	Steroids and steroid derivatives	Steroids esters		Andrastone C	C28H38O8	CC1=CC2C3(C(C(C4C2(CCC(C4(C)C)OC(=O)C)C=O)O)OC5=C(C(=O)C1(C53C(=O)OC)C)C)C	Fungus	Antibacterial	10.3390/md18050258
917	191,0667	2,03	190,0593		H+	190,063	Phenylpropanoids and polyketides	Coumarins and derivatives	Isocoumarins		7-hydroxy-3, 5-dimethyl-isochromen-1-one	C11H10O3	CC1=CC2=C(C=C(C=C2C(=O)O1)O)C	Fungus	Antifungal	10.1016/j.fitote.2011.10.013
929	197,1255	5,20	196,1181		H+	196,12	Organic acids and derivatives	Carboxylic acids and derivatives	Amino acids, peptides, and analogues	Amino acids and derivatives	Cyclo(Pro-Val)	C10H16N2O2	C1CC(N(C1)CC(CO)N)CO	Fungus	AntiQS	10.3390/biom10020298
934	245,1247	7,46	244,1173		H+	244,29	Organic acids and derivatives	Carboxylic acids and derivatives	Amino acids, peptides, and analogues	Amino acids and derivatives	Cyclo(Phe-Pro)	C14H16N2O2	C1CC2C(=O)NC(C(=O)N2C1)CC3=CC=CC=C3	Bacteria	AntiQS	10.1128/mBio.00366-13
935	213,1560	7,71	212,1486		H+	212,15	Organic acids and derivatives	Carboxylic acids and derivatives	Amino acids, peptides, and analogues	Amino acids and derivatives	Cyclo(Val-Leu)	C11H20N2O2	CC(C)CC1C(=O)NC(C(=O)N1)C(C)C	Fungus	Probiotic	10.1038/s41598-020-64374-w.
947	227,1243	12,40		204,1443	Na+	204,26	Organic acid and derivatives	Hydroxy acids and derivatives	Medium-chain hydroxy acids and derivatives		3,5-dihydroxydecanoic acid	C10H20O4	CCCCCC(CC(CC(=O)O)O)O	Fungus	Antimicrobial	10.1177/1934578X0900400315
989	307,1066	9,69	306,0992		H+	306,1103	Organoheterocyclic compounds	Benzopyrans	1-benzopyrans	Butenolides	Aspersclerotiorone F	C16H18O6	CC1CC2=C(C(=C(C=C2OC13C(=CC(=O)O3)OC)OC)O)C	Fungus	Antibacterial (AntiQS Furanone derivatives)	10.1016/j.phytochem.2017.02.008
994	261,1192	10,82		238,1392	Na+	260,1049	Phenylpropanoids and polyketides	Coumarins and derivatives	Hydroxycoumarins	Hydroxycoumarins	7-(gamma,gamma-Dimethylallyloxy)-6-hydroxy-4-methylcoumarin	C15H16O4	CC1=CC(=O)OC2=CC(=C(C=C12)O)OCC=C(C)C	Fungus	Coumarin	10.1007/s00253-018-8787-x.
996	244,1658	11,75	243,1584		H+	221,34	Organoheterocyclic compounds	Quinolines and derivatives	Quinolones and derivatives	Hydroquinolones	2-heptylquinolin-4(1H)-one	C16H21NO	CCCCCCCC1=CC(=O)C2=CC=CC=C2N1	Bacteria	QS	10.3762/bjoc.14.241
997	272,1968	13,40	271,1894		H+	271,4	Organoheterocyclic compounds	Quinolines and derivatives	Hydroquinolines		2-nonylquinolin-4(1H)-one	C18H25NO	CCCCCCCCCC1=CC(=O)C2=CC=CC=C2N1	Bacteria	Antimicrobial	10.1016/j.phytochem.2009.05.004
1003	199,0833	5,46	198,0759		H+	198,22	Organoheterocyclic compounds	Quinolines and derivatives	Isoquinolones and derivatives		3-methyl-1H-pyrazolo[1,5-b]isoquinolin-9-one	C12H10N2O	CC1=CNN2C1=CC3=CC=CC=C3C2=O	Bacteria	Antibacterial	10.7164/antibiotics.49.700
1016	183,0907	5,98	182,0833		H+	182,22	Organoheterocyclic compounds	Indoles and derivatives	Indoles	Beta carboline	3-methyl-β-carboline	C12H10N2	CC1=CC2=C(C=N1)NC3=CC=CC=C32	Bacteria	Antibacterial	10.1177/1934578X1501000627
1022	359,0760	9,52		336,0960	Na+	336,9	Polycyclic aromatic polyketides	Anthraquinones and anthrones	Anthroquinones		Methyl 3,6,8-trihydroxy-4-methoxy-1-methyl-9,10-dioxoanthracene-2-carboxylate	C16H16O8	CC1=C2C(=C(C(=C1C(=O)OC)O)OC)C(=O)C3=C(C2=O)C(=CC(=C3)O)O	Fungus	Antibacterial	10.1055/s-0029-1186143
1027	211,0859	11,36	210,0785		H+	210,23	Organoheterocyclic compounds	Indoles and derivatives	Carboline alkaloids	β-carboline	1-acetyl-β-carboline	C13H10N2O	CC(=O)C1=NC=CC2=C1NC3=CC=CC=C23	Bacteria	AntiQS (β-carboline)	10.1007/s11030-021-10347-8
1056	305,1641	13,16	304,1567		H+	304,4	Organoheterocyclic compounds	Indoles and derivatives	Carboline alkaloids	β-carboline	4-Hydroxymethyl-3-(2,4-dimethyl-7 -indolyl)-2-methylindole	C20H20N2O	CC1=C2C=C(NC2=C(C=C1)C3=C(NC4=CC=CC(=C43)CO)C)C	Fungus	AntiQS (β-carboline)	10.1007/s11030-021-10347-8
1058	319,1794	13,98	318,1720		H+	318,413	Organoheterocyclic compounds	Indoles and derivatives	Carboline alkaloids	β-carboline	4-(methoxymethyl)-2-methyl-3-[(2-methyl-1h-indol-4-yl)methyl]-1h-indole	C21H22N2O	CC1=CC2=C(C=CC=C2N1)CC3=C(NC4=CC=CC(=C43)COC)C	Fungus	AntiQS (β-carboline)	10.1007/s11030-021-10347-8
1066	347,2101	15,56		324,2301	Na+	324,5	Lipid and lipid-like molecules	Fatty acyls	Fatty alcohols		Pironetin	C19H32O4	CCC1C=CC(=O)OC1CC(C(C)C(C(C)CC=CC)OC)O	Bacteria	Plant growth simulator	10.1021/ol027211o
1091	247,1294	9,58		224,1494	Na+	224,14	Organoheterocyclic compounds	Pyrans	Pyranones and derivatives		Nipyrone A	C13H20O3	CCC(C)CC(C)C1=CC(=C(C(=O)O1)C)O	Fungus	Antibacterial	10.3390/md17060344
1191	195,1095	6,72	194,1021		H+	194,094	Organoheterocyclic compounds	Coumarins and derivatives			2,3,4-Trimethyl-5,7-dihydroxy-2,3-dihydrobenzofuran	C11H14O3	CC1C(OC2=C(C=C(C(=C12)C)O)O)C	Fungus	Coumarin	10.1007/s00253-018-8787-x.
1202	277,1390	11,83	276,1316		H+	276,28	Organoheterocyclic compounds	Coumarins and derivatives	Coumarines		Visamminol	C15H16O5	CC1=CC(=O)C2=C(C3=C(C=C2O1)OC(C3)C(C)(C)O)O	Eukaryota	Coumarin	10.1007/s00253-018-8787-x
1216	447,2816	18,30	446,2742		H+	446,6	Organic acids and derivatives	Carboxylic acids and derivatives	Amino acids, peptides and analogues	Peptides	Sclerotiotide C	C24H38N4O4	CC=CC=CC=CC(=O)NC1CCCCNC(=O)C(N(C(=O)C(N(C1=O)C)C(C)C)C)C	Fungus	Antifungal	10.1021/np100198h
1249	211,1404	6,60	210,1330		H+	210,13	Organic acids and derivatives	Carboxylic acids and derivatives	Amino acids, peptides and analogues	Amino acids and derivatives	Cyclo(Leu-Pro)	C11H18N2O2	CCC(C)C1C(=O)N2CCCC2C(=O)N1	Bacteria	AntiQS (diketopiperazines)	10.4014/jmb.1907.07030
1250	166,1089	9,05	165,1015		H+	165,11	Benzenoids	Phenols ethers	Aminiophenyl ethers		N-methyl-2-(2-methylpropoxy)aniline	C10H15NO	CC(C)COC1=CC=CC=C1N	Bacteria	Antimicrobial (Algicide)	10.3389/fmicb.2015.01235
1259	319,1351	9,60	318,1277		H+	318,32	Phenylpropanoids and polyketides	Isochromanequinones	Benzoisochromanequinones		Astropaquinone B	C17H18O6	CC1CC2=C(C(O1)OC)C(=O)C3=C(C2=O)C=C(C=C3OC)OC	Fungus	Antimicrobial	10.1055/s-0029-1185684
1287	367,1280	9,76		344,1480	Na+	344,4	Organic acids and derivatives	Carboxylic acids and derivatives	Amino acids, peptides and analogues	Alpha amino acids and derivatives	Penicillin G	C16H18N2O4S	CC1(C(N2C(S1)C(C2=O)NC(=O)CC3=CC=CC=C3)C(=O)O)C	Fungus	Antibacterial	10.1016/j.enzmictec.2007.11.013
1364	323,1038	7,94	322,0964		H+	322,3	Organic acids and derivatives	Carboxylic acids and derivatives	Amino acids, peptides, and analogues	Amino acids and derivatives	6'-[(2-hydroxyphenyl)methylidene]spiro[3H-1-benzofuran-2,3'-piperazine]-2',5'-dione	C18H14N2O4	C1C2=CC=CC=C2OC13C(=O)NC(=CC4=CC=CC=C4O)C(=O)N3	Fungus	Antimicrobial	10.1021/acs.orglett.6b02620
1368	136,0758	8,29	135,0684		H+	135,068	Organic oxygen compounds	Organooxygen compounds	Carboxyl compounds	Ketones	2-Aminoacetophenone	C8H9NO	CC(=O)C1=CC=CC=C1N	Bacteria	Pro QS	10.1128/mbio.00159-23
1369	305,1376	8,74		282,1576	Na+	282,16	Organoheterocyclic compounds	Pyranones and derivatives	Pyrrolines		Cladosin A	C14H22N2O4	CC(CC(CC(=N)C1=C(C(=C(C)C)NC1=O)O)OC)O	Fungus	Antimicrobial (Antiviral)	10.1021/np400833x
1372	334,1539	9,38	333,1465		H+	333,4	Organic acids and derivatives	Carboxylic acids and derivatives	Amino acids, peptides and analogues	Amino acids and derivatives	Isorugulosuvine (Cyclo(Trp-Phe)	C20H19N3O2	C1=CC=C(C=C1)CC2C(=O)NC(C(=O)N2)CC3=CNC4=CC=CC=C43	Fungus	Cyclopeptides	10.3390/biom10020298
1393	194,1141	9,12	193,1067		H+	193,11	Organoheterocyclic compounds	Pyridines and derivatives	Pyridine alkaloids	Pyridinecarboxylic acids	Methyl fusarate	C11H15NO2	CCCCC1=CN=C(C=C1)C(=O)OC	Fungus	Phytotoxin	10.1021/jf981306g.
1433	164,1037	8,03	163,0963		H+	163,17	Organic oxygen compounds	Organooxygen compounds	Carbohydrates and carbohydrates conjugated	Monosaccharides	Mycosamine	C6H13NO4	CC(O)C(O)C(N)C(O)C=O	Bacteria	Antifungal	10.1021/ol060353o.
1436	243,0833	8,44	242,0759		H+	242,23	Organoheterocyclic compounds	Pteridines and derivatives	Alloxazines and isoalloxazines	Flavins	Riboflavin lumichrome	C12H10N4O2	CC1=CC2=C(C=C1C)N=C3C(=N2)C(=O)NC(=O)N3	Fungus	Pro QS	10.1094/MPMI-21-9-1184
1454	317,1301	11,83		294,1501	Na+	294,34	Organoheterocyclic compounds	Coumarins and derivatives	Isocoumarins	Dihydroisocoumarines	Aspergimarin D	C16H22O5	CC(CCCC1CC2=C(C(=C(C=C2)OC)OC)C(=O)O1)O	Fungus	Plant signaling/antibacterial (+ coumarin)	10.3389/fmicb.2019.02846
1477	420,1604	11,14	419,1530		H+	419,4	Organic acids and derivatives	Carboxylic acids and derivatives	Amino acids, peptides and analogues	Alpha amino acids and derivatives	Sorbicillactone B	C21H25NO8	CC=CCCC(=O)C1=C(C(=C(C2(C1C(C(=O)O2)(C)NC(=O)C=CC(=O)O)C)O)C)O	Fungus	Antimicrobial	10.3390/md502023
1487	191,0811	6,47	190,0737		H+	190,07	Organoheterocyclic compounds	Diazanaphtalenes	Benzodiazines	Quinazolines	2-(1-hydroxyethyl)-4(3H)quinazoline	C8H12N2O2	CC(C1=NC2=CC=CC=C2C(=O)N1)O	Fungus	Quinazoline antiQS	10.1016/j.bioorg.2022.106226.
1488	211,1436	6,60	210,1362		H+	210,13	Organic acids and derivatives	Carboxylic acids and derivatives	Amino acids, peptides, and analogues	Amino acids and derivatives	Cyclo(Leu-Pro)	C11H18N2O2	CC(C)CC1C(=O)N2CCCC2C(=O)N1	Bacteria	AntiQS (diketopiperazines)	10.4014/jmb.1907.07030
1504	217,0645	1,07		194,0845	Na+	194,09	Organoheterocyclic compounds	Benzopyrans	1-benzopyrans		5-methoxy-2-methyl-3,4-dihydro-2h-1-benzopyran-4-ol	C11H14O3	CC1CC(C2=C(O1)C=CC=C2OC)O	Fungus	Antibacterial	10.1080/14786419.2015.1072712
1520	320,1671	6,01		297,1871	Na+	297,157	Organic oxygen compounds	Organooxygen compounds	Carbonyl compounds	Ketones	Atpenin B	C15H23NO5	CCC(C)CC(C)C(=O)C1=C(C(=C(NC1=O)OC)OC)O	Fungus	Antifungal	10.7164/antibiotics.43.1553
1573	229,0970	5,93		206,1170	Na+	206,09	Organoheterocyclic compounds	Benzopyrans			8-methoxy-3,5-dimethyl-3,4-dihydroisochromen-6-one	C12H14O3	CC1CC2=C(C(=O)C=C(C2=CO1)OC)C	Fungus	Antifungal	10.1021/np0498859
1609	233,1131	9,54		210,1331	Na+	210,27	Phenylpropanoids and polyketides	Macrolides and analogues			Patulolide B	C12H18O3	CC1CCCCCCC(=O)C=CC(=O)O1	Fungus	Antimicrobial	10.1021/jo0264776
1614	219,0981	12,44		196,1181	Na+	196,11	Organoheterocyclic compounds	Pyrans	Pyranones and derivatives		10-hydroxymucidone	C11H16O3	CCC1=CC=C(CC(C)(C)O)C(=O)O1	Bacteria	Antibacterial	10.1021/acs.jnatprod.6b00175
1615	235,1292	12,67		212,1492	Na+	234,29	Lipid and lipid-like molecules	Prenol lipids	Monoterpenoids	Acyclic monoterpenoids	5,9-dimethyl-10-oxododeca-2,4,6,8-tetraenoic acid	C14H18O3	CCC(=O)/C(=C\C=C\C(=C/C=C/C(=O)O)\C)/C	Bacteria	Antibacterial	10.1007/s10600-014-0970-4

Name	Putative identification	MEA 75% NSW						
		QQ short-HSL		QQ long-HSL		QQ AI-2	Antibiofilm	
		MT102	CV026	MT102	F117	MM32	*V. harveyi*	*Labrenzia sp.*
Li1	Aspergillus versicolor	0	0	0	0	1	0	0
Li2	Aspergillus versicolor	0	0	0	0	1	0	0
Ch1	Aspergillus versicolor	0	0	0	0	1	0	0
Ch2	Aspergillus versicolor	1	0	1	0	1	1	0
Ch3	Aspergillus versicolor	0	1	0	0	1	0	0
Ch4	Aspergillus versicolor	0	1	0	1	0	1	0
Ch5	Aspergillus versicolor	0	1	0	0	0	0	0
Ch6	Geomyces pannorum	0	0	1	0	1	1	0
Ch7	Aspergillus versicolor	0	1	0	0	1	0	0
Ch8	Aspergillus versicolor	0	1	0	0	1	0	0
Ch11	Aspergillus versicolor	0	1	0	0	1	0	0
V1	Penicillium chrysogenum	1	1	1	1	1	0	0
V2	Penicillium atramentosum	1	1	1	1	1	1	1
V3	Penicillium chrysogenum	1	1	1	1	1	0	0
V4	Penicillium chrysogenum	1	1	1	1	1	0	1
V5	Penicillium citreonigrum	1	1	0	1	1	0	1

G1	Penicillium chrysogenum	1	0	0	1	1	0	1
G2	Penicillium chrysogenum	1	1	1	0	1	0	1
G3	Penicillium chrysogenum	1	0	0	0	1	1	0
G4	Penicillium chrysogenum	0	0	0	1	1	0	0
G5	Penicillium chrysogenum	0	0	0	1	1	0	0
G6	Penicillium chrysogenum	0	1	0	1	0	0	1
A1	Acremonium fuci	0	0	0	1	1	0	0
A2	Acremonium fuci	0	1	0	1	1	0	0
A3	Penicillium citreonigrum	1	1	0	1	1	0	1
A4	Acremonium fuci	1	1	0	1	1	1	1
A5	Acremonium hyalinulum	0	0	0	1	1	0	1
A6	Acremonium fuci	1	0	1	1	1	0	0
B1	Penicillium chrysogenum	0	1	0	1	1	0	1
B2	Penicillium chrysogenum	0	1	0	1	1	0	1
B3	Penicillium chrysogenum	1	1	1	1	1	0	1
B4	Penicillium chrysogenum	0	0	0	1	1	0	1
B5	Penicillium chrysogenum	0	1	0	1	1	1	1
F1	Penicillium citreonigrum	0	0	0	0	0	1	0
F2	Penicillium rubens	0	1	0	1	1	0	1

F3	Penicillium citreonigrum	0	1	0	1	0	1	1
F4	Penicillium chrysogenum	0	1	0	1	1	0	1
F5	Penicillium chrysogenum	0	0	1	1	1	0	1
L	Gibberella intricans	0	1	0	1	1	0	0
Q1	Penicillium citreonigrum	1	1	1	1	0	0	0
Q2	Penicillium citreonigrum	0	1	0	1	1	1	0
P	Paradendryphiella salina	1	0	1	1	0	0	0

Name	Putative identification	PDB 75% NSW						
		QQ short-HSL		QQ long-HSL		QQ AI-2	Antibiofilm	
		MT102	CV026	MT102	F117	MM32	*V. harveyi*	*Labrenzia sp.*
Li1	Aspergillus versicolor	0	1	0	0	0	1	1
Li2	Aspergillus versicolor	0	0	0	0	0	1	0
Ch1	Aspergillus versicolor	0	1	0	0	0	1	1
Ch2	Aspergillus versicolor	0	0	0	1	0	1	0
Ch3	Aspergillus versicolor	0	1	0	0	0	1	0
Ch4	Aspergillus versicolor	0	0	0	0	0	1	0
Ch5	Aspergillus versicolor	0	0	0	0	0	1	0
Ch6	Geomyces pannorum	0	0	0	0	0	0	0
Ch7	Aspergillus versicolor	0	0	0	0	0	0	0
Ch8	Aspergillus versicolor	0	0	0	0	0	1	0
Ch11	Aspergillus versicolor	0	1	0	0	0	0	0
V1	Penicillium chrysogenum	0	1	1	0	1	0	0
V2	Penicillium atramentosum	1	1	1	0	1	0	0
V3	Penicillium chrysogenum	1	1	0	1	0	0	0
V4	Penicillium chrysogenum	1	1	1	1	0	0	0
V5	Penicillium citreonigrum	0	0	0	0	0	0	0

G1	Penicillium chrysogenum	1	1	1	1	0	0	0
G2	Penicillium chrysogenum	0	1	0	1	0	0	0
G3	Penicillium chrysogenum	0	1	1	1	0	1	0
G4	Penicillium chrysogenum	0	1	1	0	0	0	0
G5	Penicillium chrysogenum	0	1	0	1	0	0	0
G6	Penicillium chrysogenum	0	1	0	0	0	0	0
A1	Acremonium fuci	0	0	0	1	0	1	0
A2	Acremonium fuci	0	0	0	1	0	1	0
A3	Penicillium citreonigrum	1	0	1	0	0	1	0
A4	Acremonium fuci	1	0	1	1	0	0	0
A5	Acremonium hyalinulum	0	0	0	1	0	0	0
A6	Acremonium fuci	0	0	0	1	0	0	0
B1	Penicillium chrysogenum	0	0	0	1	0	0	0
B2	Penicillium chrysogenum	0	1	0	1	1	0	0
B3	Penicillium chrysogenum	0	1	0	1	1	1	0
B4	Penicillium chrysogenum	0	1	0	1	0	0	0
B5	Penicillium chrysogenum	0	0	0	1	0	0	0
F1	Penicillium citreonigrum	1	0	1	1	0	0	0
F2	Penicillium rubens	0	1	0	1	1	1	0

F3	Penicillium citreonigrum	1	1	1	1	0	0	0
F4	Penicillium chrysogenum	0	1	0	1	1	0	0
F5	Penicillium chrysogenum	0	0	0	1	1	1	0
L	Gibberella intricans	0	0	1	1	1	1	0
Q1	Penicillium citreonigrum	1	0	1	1	1	1	0
Q2	Penicillium citreonigrum	1	1	1	1	1	0	0
P	Paradendryphiella salina	1	0	1	1	1	0	0

Name	Putative identification	QS (HSL and AI-2)				QQ short-HSL (C6-HSL)		QQ long-HSL (oxoC10-HSL)		QQ AI-2	Biofilm formation	Antibiofilm	
		**MM32** (AI-2)	**CV026** (short-HSL)	**MT102** (medium-HSL)	**F117** (long-HSL)	**MT102**	**CV026**	**MT102**	**F117**	**MM32**		***Vibrio harveyi***	***Labrenzia sp.* 2184**
4	*Pseudoalteromonas aliena*	0	0	0	0	1	0	1	1	0	1	1	1
5	*Pseudoalteromonas aliena*	0	0	0	0	1	0	1	1	0	1	0	1
12	*Pseudoalteromonas aliena*	0	0	0	0	1	0	1	1	0	1	0	0
15	*Pseudoalteromonas aliena*	0	0	0	0	0	1	1	0	0	1	0	1
16	*Pseudoalteromonas aliena*	0	0	0	0	1	0	1	1	0	1	0	1
17b	*Paraglaciecola marina*	1	0	0	0	0	0	0	1	0	1	0	0
19	*Alkalihalobacillus hwajinpoensis*	1	0	0	0	0	0	1	1	0	0	0	0
29	*Pseudoalteromonas aliena*	0	0	0	0	1	0	1	1	0	1	0	1
32	*Pseudoalteromonas aliena*	0	0	0	0	1	1	1	1	0	1	1	1
33	*Pseudoalteromonas aliena*	1	0	0	0	1	1	1	1	0	1	0	0
38	*Metabacillus litoralis*	1	0	0	0	0	1	0	0	0	0	0	0
41	*Brevibacterium frigoritolerans*	1	0	0	0	1	0	1	1	0	0	0	0

44	*Granulosicoccus coccoides*	1	0	0	0	1	0	0	1	0	1	0	0
48	*Nocardiopsis deserti*	1	0	0	0	1	0	0	1	0	1	0	0
49	*Pseudoalteromonas aliena*	0	0	0	0	1	0	1	1	0	1	0	0
51	*Granulosicoccus coccoides*	1	0	0	0	1	0	0	1	0	0	0	0
53	*Pseudoalteromonas aliena*	0	0	0	0	1	0	1	1	0	1	1	0
65	*Sulfitobacter donghicola*	1	0	0	0	0	0	0	1	0	0	0	0
74	*Paraglaciecola mesophila*	1	0	0	0	0	0	1	0	0	0	0	0
80	*Loktanella ponticola*	1	0	0	0	0	0	0	1	0	0	0	0
84	*Pseudoalteromonas translucida*	0	0	0	0	1	0	1	1	0	1	0	1
85	*Pseudoalteromonas translucida*	0	0	0	0	1	1	1	1	0	1	0	0
86	*Pseudoalteromonas translucida*	0	0	0	0	1	0	1	1	0	1	0	0
87	*Pseudoalteromonas nigrifaciens*	0	0	0	0	1	0	1	1	0	1	0	0
88	*Vibrio sp.*	1	0	0	0	1	0	1	1	0	1	0	1
89	*Pseudoalteromonas translucida*	0	0	0	0	1	1	1	1	0	1	0	1
90	*Cytobacillus kochii*	1	0	0	0	1	0	1	1	0	1	0	0

91	*Pseudoalteromonas aliena*	0	0	0	0	1	1	1	1	0	1	0	1
92	*Cytobacillus kochii*	1	0	0	0	1	0	1	1	0	1	0	0
93	*Pseudoalteromonas aliena*	0	0	0	0	1	0	1	1	0	1	0	1
94	*Pseudoalteromonas aliena*	0	0	0	0	1	0	1	1	0	1	0	1
95	*Pseudoalteromonas aliena*	0	0	0	1	1	0	1	0	0	1	0	1
96	*Pseudoalteromonas aliena*	0	0	0	0	1	0	1	1	0	1	0	1
97	*Pseudoalteromonas aliena*	0	0	0	0	1	1	1	0	0	1	0	1
98	*Brevibacterium frigoritolerans*	0	0	0	0	1	0	1	1	0	1	0	0
O1	*Brumimicrobium aurantiacum*	0	0	0	0	1	0	1	1	0	1	0	0
O2	*Planococcus maritimus*	1	0	0	0	1	0	1	1	0	1	0	0
O3	*Exiguobacterium aurantiacum*	1	0	0	0	1	0	1	1	0	0	1	1
O4	*Planococcus maritimus*	1	0	0	0	1	1	1	1	0	0	1	0
O5	*Zobellia sp.*	0	0	0	0	1	0	1	1	0	0	1	0
O7	*Alkalihalobacillus algicola*	1	0	0	0	1	0	1	1	0	0	0	0
O8	*Nonlabens xylanidelens*	0	0	0	0	1	0	1	1	0	1	0	0

O9	*Shewanella ulleungensis*	1	0	0	0	1	0	1	1	0	1	0	1
O10	*Shewanella ulleungensis*	1	0	0	1	1	1	1	1	0	0	0	1
O11	*Paraglaciecola marina*	0	0	0	0	1	0	1	1	0	1	1	0
JO1	*Algibacter pacificus*	0	0	0	0	1	0	0	0	0	0	0	0
JO2	*Algibacter miyuki*	0	0	0	0	1	0	0	0	0	0	1	0
JO8	*Maribacter forsetii*	0	0	0	0	1	0	0	0	0	0	0	0
JO9	*Maribacter caenipelagi*	0	0	0	0	1	0	1	1	0	1	0	0
JO10	*Algibacter pacificus*	1	0	0	0	1	0	1	0	0	1	0	0
JO12	*Winogradskyella eximia*	1	0	0	0	1	0	1	0	0	0	0	0
JO13	*Algibacter pacificus*	1	0	0	1	1	0	1	0	0	1	0	0
JO14a	*Algibacter miyuki*	1	0	0	0	1	0	0	0	0	0	0	0
JO14b	*Algibacter miyuki*	1	0	0	0	1	0	1	0	0	0	0	0
JO15	*Algibacter miyuki*	1	0	0	1	1	0	1	0	0	0	0	0
JO17a	*Maribacter forsetii*	1	0	0	1	1	0	1	0	0	1	0	1
JO17b	*Maribacter forsetii*	1	0	0	0	1	0	1	0	1	1	0	1

JO20	*Winogradskyella undariae*	1	0	0	0	1	0	1	0	1	0	0	0
JO21	*Winogradskyella undariae*	0	0	0	1	1	0	1	0	0	0	0	1
JO22	*Dokdonia donghaensis*	1	0	0	0	1	0	0	0	1	0	0	0
JO23	*Dokdonia donghaensis*	0	0	0	0	1	0	0	0	0	1	0	1
JO25	*Dokdonia donghaensis*	1	0	0	1	1	0	1	0	0	1	0	1
JO26	*Dokdonia donghaensis*	1	0	0	0	0	0	0	0	0	1	0	1
JO27	*Winogradskyella undariae*	0	0	0	0	1	0	0	0	1	0	0	1
JO29	*Winogradskyella arenosi*	1	0	0	0	1	0	0	0	0	0	0	0
JO30	*Formosa algae*	0	0	0	0	1	0	1	0	0	0	0	1
JO31	*Maribacter forsetii*	0	0	0	0	1	0	0	0	0	1	0	0
JO32	*Maribacter forsetii*	1	0	0	0	1	0	1	0	1	1	0	0
JO33	*Algibacter pacificus*	0	0	0	0	1	0	1	0	0	1	0	0
JO35	*Polaribacter sejongensis*	1	0	0	0	1	0	1	0	0	0	0	1
JO36a	*Polaribacter sejongensis*	1	0	0	0	1	0	1	1	0	0	0	1
JO36b	*Algibacter pacificus*	1	0	0	0	1	0	1	0	0	1	0	0

JO37	*Algibacter wandonensis*	0	0	0	0	1	0	1	0	0	1	0	0
JO39	*Maribacter spongiicola*	0	0	0	0	0	0	0	0	0	0	0	1
JO40	*Zobellia sp.*	0	0	0	0	0	0	0	0	0	1	0	0
JO41	*Zobellia sp.*	0	0	0	1	0	0	0	0	0	1	0	0
JO42	*Zobellia sp.*	1	0	0	1	0	1	0	0	0	0	0	0
JO43	*Winogradskyella undariae*	1	0	0	1	0	1	1	0	1	0	0	1
JO44a	*Maribacter forsetii*	1	0	0	1	0	1	0	0	0	1	0	0
JO44b	*Maribacter forsetii*	0	0	0	1	0	1	0	0	0	0	0	0
JO45	*Maribacter spongiicola*	1	0	0	1	0	0	0	0	1	1	0	0
JO46	*Formosa sp.*	0	0	0	1	0	1	0	0	0	1	0	1
JO47	*Maribacter forsetii*	0	0	0	1	0	0	0	0	0	1	0	1
JO48	*Algibacter miyuki*	0	0	0	1	0	1	0	0	0	0	0	0
JO49	*Maribacter forsetii*	0	0	0	1	0	0	0	0	0	1	0	0
JO50	*Algibacter pectinivorans*	0	0	0	1	0	0	0	0	0	1	0	0
JO51	*Algibacter miyuki*	1	0	0	1	0	1	0	0	0	1	0	0

JO52	*Algibacter miyuki*	0	0	0	1	1	1	1	0	0	0	0	0
Jf2	*Microbacterium diaminobutyricum*	0	0	0	1	1	0	1	0	0	0	0	0
Jf3	*Microbacterium diaminobutyricum*	0	0	0	1	1	1	0	1	1	0	0	0
Jf9	*Lacinutrix undariae*	0	0	0	1	0	0	0	0	0	0	1	1
Jf16	*Micrococcus endophyticus*	0	0	0	1	0	1	0	0	0	0	0	0
Jf17	*Microbacterium diaminobutyricum*	0	0	0	1	1	0	1	0	0	0	0	0
Jf18	*Winogradskyella helgolandensis*	0	0	0	1	0	1	0	0	0	0	1	1
Jf19	*Salinibacterium amurskyense*	0	0	0	1	0	1	0	0	0	1	0	0
Jf20	*Maribacter forsetii*	0	0	0	1	0	1	0	1	0	0	0	1
Jp1	*Kocuria palustris*	0	0	0	1	0	1	0	0	0	1	1	0
Jp3	*Formosa algae*	0	0	0	1	0	0	0	0	0	1	0	0
Jp8	*Knoellia subterranea*	0	0	0	0	1	0	1	1	0	0	0	0
Jp9	*Winogradskyella undariae*	0	0	0	0	0	1	0	0	1	0	0	1
Jp10	*Colwellia sp.*	0	0	0	1	1	1	1	0	0	0	0	0
Jp11	*Maribacter forsetii*	1	0	0	1	0	1	0	0	0	1	0	0

Jp12	*Formosa algae*	0	0	0	1	0	1	0	1	0	1	0	0
J1	*Winogradskyella sp.*	0	0	0	0	0	1	1	1	0	0	0	0
J2	*Formosa algae*	0	0	0	0	1	0	0	0	0	0	0	1
J3	*Maribacter forsetii*	0	0	0	0	1	0	0	0	0	0	0	0
J4	*Algibacter miyuki*	1	0	0	0	1	0	0	0	0	1	0	0
J5	*Wenyingzhuangia heitensis*	1	0	0	0	1	0	1	0	0	1	0	0
R1	*Shewanella electrodiphila*	1	0	0	0	0	0	0	0	0	1	0	1
R2	*Pseudoalteromonas nigrifaciens*	0	0	0	0	0	0	0	0	0	1	0	1
R3	*Vibrio sp.*	0	0	0	0	0	0	1	0	0	1	0	1
R4	*Shewanella ulleungensis*	0	0	0	1	1	1	1	0	0	1	0	1
R5	*Shewanella electrodiphila*	1	0	0	0	0	0	1	0	0	0	0	1
R6	*Algoriphagus winogradskyi*	1	0	0	0	0	0	1	0	0	1	0	0
Mnb2	*Pseudomonas azotoformans*	1	0	0	0	1	1	0	0	1	0	0	1
Mnb4	*Oceanobacillus massiliensis*	1	0	0	0	1	1	1	0	1	1	0	1
Mnb5	*Colwellia sp.*	0	0	0	0	0	0	0	0	0	1	0	1

Mnb6	*Sulfitobacter donghicola*	1	0	0	0	0	0	0	0	0	1	0	0
Mnb7	*Sulfitobacter donghicola*	1	0	0	0	1	0	1	1	0	1	0	0
Mnb8	*Sulfitobacter donghicola*	1	0	0	0	1	0	0	0	0	0	0	0
Mnb9	*Sulfitobacter donghicola*	1	0	0	0	0	0	0	0	0	0	0	1
Mnb10	*Planococcus maritimus*	0	0	0	0	1	1	1	0	0	0	0	0
Mnb11	*Shewanella ulleungensis*	0	0	1	0	0	0	0	0	0	0	0	0
Mnb12	*Phaeobacter sp.*	1	0	0	0	0	0	0	0	0	0	0	0
Mnb13	*Sulfitobacter donghicola*	1	0	0	0	0	0	1	0	0	1	0	0
Mnb14	*Phaeobacter sp.*	1	0	0	0	1	1	1	1	0	0	0	0
Mnb15	*Sulfitobacter donghicola*	1	0	0	0	0	0	0	0	0	0	0	0
Mnb16	*Pseudomonas azotoformans*	0	0	0	0	0	0	0	0	0	0	0	0
Mc1	*Vibrio sp.*	1	0	0	0	0	0	0	0	0	0	0	0
Mc2	*Shewanella denitrificans*	1	0	0	0	1	0	1	0	0	0	0	0
Mc4	*Paraglaciecola mesophila*	0	0	0	0	0	0	0	0	0	1	0	0
Mc5	*Pseudoalteromonas nigrifaciens*	0	0	0	0	1	0	0	0	0	1	0	1

Mc6	*Pseudoalteromonas aliena*	0	0	0	0	1	0	0	0	0	1	0	1
Mc7	*Pseudoalteromonas aliena*	0	0	0	0	1	0	0	0	0	0	0	1
Mc8	*Shewanella denitrificans*	1	0	0	0	1	0	0	0	0	0	0	0
Mc9	*Pseudomonas azotoformans*	1	0	0	0	1	0	0	0	0	1	0	1
Mc10	*Phaeobacter sp.*	0	0	0	0	1	0	1	1	0	0	1	0
Mc11	*Pseudomonas azotoformans*	1	0	0	0	1	0	0	0	0	0	0	1
Mc12	*Pseudomonas japonica*	0	0	0	0	1	1	1	0	0	0	1	0
Mc13	*Pseudomonas azotoformans*	1	0	0	0	1	0	0	0	0	0	0	0
Mc14	*Pseudoalteromonas nigrifaciens*	0	0	0	0	1	0	0	0	0	0	0	0
Mc15	*Pseudomonas japonica*	1	0	0	0	1	0	0	0	0	0	0	1
Mb1	*Pseudomonas azotoformans*	0	0	0	0	1	0	0	0	0	0	0	1
Mb3a	*Pseudomonas azotoformans*	1	0	0	0	1	0	0	0	0	0	0	1
Mb3b	*Pseudomonas azotoformans*	1	0	0	0	1	0	0	0	0	0	0	1
Mb4	*Pseudomonas azotoformans*	1	0	0	0	1	0	1	0	0	0	0	1
Mb5	*Pseudomonas azotoformans*	1	0	0	0	1	0	0	0	0	0	0	1

Mb6	*Pseudomonas azotoformans*	1	0	0	0	1	1	0	0	0	0	0	1
Mb7	*Pseudomonas azotoformans*	1	0	0	1	1	1	0	0	0	0	0	1
Mb10	*Pseudomonas azotoformans*	1	0	0	0	1	0	1	0	0	0	0	1
Mb11	*Loktanella ponticola*	1	0	0	0	1	0	0	0	0	0	0	0
Mb12	*Pseudomonas azotoformans*	1	0	0	0	1	0	0	0	0	0	0	0
Mb13	*Psychromonas sp.*	1	0	0	0	1	0	0	0	0	0	0	0
Mb14	*Pseudomonas japonica*	1	0	0	0	1	0	0	0	1	0	0	0
Mb16	*Pseudomonas azotoformans*	1	0	0	0	1	0	0	0	0	0	0	1
Mb17	*Pseudomonas azotoformans*	1	0	0	0	1	0	0	0	0	1	0	1
Mb18	*Pseudomonas azotoformans*	0	0	0	0	1	0	1	0	0	0	0	1
Mb20	*Psychrobacter fozii*	1	0	0	0	1	0	0	0	0	1	0	1
Mb22	*Pseudomonas azotoformans*	0	0	0	1	1	0	1	0	0	1	0	0
Mb24	*Pseudomonas japonica*	0	0	0	1	1	0	1	1	0	1	1	0
Mb26	*Pseudoalteromonas nigrifaciens*	0	0	0	1	1	0	1	1	0	0	1	0
Mb27	*Pseudomonas azotoformans*	0	0	0	1	1	0	1	0	0	0	0	0

Mb28	*Pseudomonas azotoformans*	0	0	0	1	1	0	1	0	0	0	0	1
Mnt2	*Microbulbifer thermotolerans*	0	0	0	1	1	0	1	0	0	0	0	0
Mnt3	*Pseudoalteromonas nigrifaciens*	0	0	0	1	1	0	1	0	0	1	0	1
Mnt4	*Pseudoalteromonas aliena*	0	0	0	1	1	0	1	0	1	1	0	1
Mnc1	*Pseudomonas azotoformans*	0	0	0	1	1	1	1	0	0	1	0	0
Mnc2	*Pseudomonas azotoformans*	0	0	0	1	1	1	1	0	0	1	0	0
Mnc3	*Loktanella ponticola*	0	0	0	1	1	0	1	0	0	1	1	0
b2	*Colwellia sp.*	0	0	0	1	1	0	1	1	0	1	1	0
b4	*Colwellia sp.*	0	0	0	1	1	0	0	0	0	1	1	0
b5	*Colwellia sp.*	0	0	0	1	1	0	1	0	0	0	0	0
b7	*Pseudomonas azotoformans*	0	0	0	1	1	1	1	1	0	0	0	0
b8	*Sulfitobacter donghicola*	0	0	0	1	1	0	1	0	0	1	0	0
b9	*Pseudomonas azotoformans*	0	0	0	1	1	1	1	0	0	0	0	0
b10	*Pseudomonas azotoformans*	0	0	0	1	1	1	1	0	0	0	0	0
b11	*Pseudoalteromonas aliena*	0	0	0	1	1	0	1	0	0	1	1	1

b12	*Pseudomonas azotoformans*	0	0	0	1	1	1	1	0	0	1	0	0
b13	*Vibrio sp.*	1	0	0	1	1	0	1	0	0	0	1	0
b14	*Cytobacillus firmus*	0	0	0	1	1	11	1	0	0	1	1	1
b15	*Pseudoalteromonas rhizosphaerae*	0	0	0	1	1	1	1	0	0	1	0	1
b16	*Colwellia sp.*	0	0	0	1	1	0	1	0	0	1	0	0
b17a	*Bacillus altitudinis*	1	0	0	1	1	0	1	0	0	0	0	0
b17b	*Bacillus altitudinis*	0	0	0	1	1	0	1	0	0	1	0	1
b19	*Colwellia sp.*	0	0	0	1	1	0	1	0	0	1	0	0
b20	*Bacillus altitudinis*	1	0	0	0	1	0	1	0	0	1	0	1
b21	*Psychromonas sp.*	0	0	0	0	1	0	1	0	0	1	0	0
b22	*Vibrio sp.*	1	0	0	0	1	0	1	0	0	0	0	0
b23	*Sulfitobacter sp.*	0	0	0	0	0	0	0	0	0	1	0	0
b24	*Pseudomonas azotoformans*	0	0	0	0	1	0	1	0	0	1	0	1
b25	*Pseudomonas azotoformans*	0	0	0	0	1	0	1	1	0	0	0	1
b26	*Pseudomonas japonica*	0	0	0	0	1	0	1	1	0	1	0	0

b27	*Pseudomonas azotoformans*	0	0	0	0	1	0	1	0	0	1	0	1
b28	*Pseudomonas azotoformans*	0	0	0	0	1	0	1	0	0	1	0	0
b29	*Phaeobacter sp.*	0	0	0	0	1	0	1	1	0	1	0	0
b30	*Pseudomonas azotoformans*	0	0	0	0	1	0	1	1	0	1	0	0
b31	*Pseudomonas azotoformans*	0	0	0	0	1	0	1	1	0	1	0	0
b32	*Bacillus pumilus*	1	0	0	0	1	0	1	1	0	0	0	0
b33	*Pseudomonas azotoformans*	0	0	0	0	1	0	1	1	0	1	0	1
b34	*Psychrobacter nivimaris*	0	0	0	0	1	0	1	1	0	0	0	0
b35	*Cobetia litoralis*	1	0	0	0	1	0	1	1	0	1	0	0
b36	*Pseudoalteromonas aliena*	0	0	0	0	1	0	1	0	0	0	0	1
b37	*Psychrobacter fozii*	0	0	0	0	1	0	1	1	0	0	0	1
b38	*Nioella sp.*	0	0	0	0	1	0	1	1	0	1	0	0
b39	*Pseudomonas azotoformans*	0	0	0	0	1	0	1	0	0	1	0	1
b40	*Ruegeria meonggei*	0	0	0	0	1	0	1	0	0	0	0	0
b41	*Pseudomonas azotoformans*	0	0	0	0	1	0	1	1	0	1	0	0

b42	*Pseudoalteromonas aliena*	0	0	0	0	1	0	1	1	0	1	0	1
b43	*Brevibacterium frigoritolerans*	1	0	0	0	1	0	1	0	0	1	0	0
b44	*Wenyingzhuangia sp.*	0	0	0	0	1	0	1	0	0	0	0	0
b48	*Paracoccus yeei*	0	0	0	1	1	0	1	0	0	1	0	0
b50	*Pseudomonas azotoformans*	0	0	0	1	1	0	1	1	0	1	0	0
b51	*Phaeobacter sp.*	0	0	0	1	1	0	1	1	0	0	0	0
b54	*Sulfitobacter donghicola*	0	0	0	1	1	0	0	0	0	0	0	0
b56.1	*Pseudomonas japonica*	0	0	0	1	1	0	1	0	0	1	0	0
b57	*Pseudomonas azotoformans*	0	0	0	1	1	0	1	1	0	0	1	1
C1	*Vibrio sp.*	1	0	0	0	1	0	1	0	0	1	0	0
C2	*Pseudoalteromonas nigrifaciens*	0	0	0	0	1	0	1	0	0	1	0	1
C3	*Vibrio sp.*	1	0	0	1	1	0	1	0	0	1	0	0
C4	*Pseudoalteromonas rhizosphaerae*	0	0	0	1	1	1	1	1	0	1	0	1
C5	*Pseudoalteromonas nigrifaciens*	0	0	0	1	1	1	1	1	0	1	0	0
C6	*Pseudoalteromonas translucida*	0	0	0	0	1	0	1	0	0	1	0	1

C7	*Pseudomonas azotoformans*	0	0	0	1	1	0	1	0	0	0	0	0
C8	*Vibrio sp.*	1	0	0	0	1	0	1	1	0	1	0	0
i1	*Sulfitobacter donghicola*	0	0	0	1	1	0	1	1	0	0	0	0
i2	*Sulfitobacter donghicola*	0	0	0	1	1	0	0	0	0	1	0	0
i3	*Sulfitobacter donghicola*	0	0	0	1	1	0	1	0	0	0	0	1
i4	*Pseudomonas azotoformans*	0	0	0	1	1	0	1	1	0	0	0	0
i5	*Pseudomonas azotoformans*	0	0	0	1	1	0	1	1	0	1	0	0
i6	*Pseudomonas japonica*	0	0	0	1	1	1	0	0	0	1	0	0
i7	*Pseudomonas mucoides*	0	0	0	1	1	0	1	0	0	0	0	0
i9	*Salinibacterium amurskyense*	0	0	0	1	1	0	0	0	0	1	0	0
N4	*Cobetia litoralis*	1	0	0	1	0	1	0	0	0	1	0	0
N5	*Cobetia amphilecti*	1	0	0	0	0	1	0	0	0	1	0	0
N6	*Cobetia amphilecti*	1	0	0	1	1	1	0	0	0	1	0	0
N7	*Staphylococcus sp.*	1	0	0	1	0	0	1	0	0	1	0	0
N8	*Rothia sp.*	1	0	0	1	0	0	1	0	0	1	0	0

N9	*Rothia sp.*	1	0	0	1	1	0	0	0	0	1	0	0
N10	*Rothia sp.*	1	0	0	1	0	0	0	0	0	1	0	0
N11	*Bacillus licheniformis*	1	0	0	1	0	0	1	0	0	0	0	0
N12	*Cobetia litoralis*	1	0	0	1	0	1	1	0	0	1	0	0
N13	*Cobetia amphilecti*	1	0	0	0	0	1	0	0	0	1	0	0
N14	*Cobetia amphilecti*	1	0	0	1	0	0	1	0	0	1	0	0
N15.1	*Priestia flexa*	1	0	0	0	0	0	1	0	0	1	0	0
N15.2	*Priestia flexa*	1	0	0	0	1	0	1	0	0	1	0	0
N16	*Cobetia amphilecti*	1	0	0	1	1	1	1	0	0	1	0	1
N17	*Cobetia amphilecti*	1	0	0	0	0	1	0	0	0	1	0	1
N18	*Dermacoccus sp.*	1	0	0	1	0	0	0	0	0	1	0	0
N19	*Staphylococcus sp.*	1	0	0	1	0	0	0	0	0	1	0	0
N20	*Cobetia amphilecti*	1	0	0	0	0	1	1	0	0	1	0	1
N21	*Cobetia amphilecti*	1	0	0	0	0	1	0	0	0	1	0	1
N22	*Cobetia litoralis*	1	0	0	1	0	0	0	0	0	1	0	0

N24	*Cellulophaga pacifica*	0	0	0	1	0	0	1	0	0	1	0	0
N26	*Cobetia amphilecti*	1	0	0	1	1	1	1	0	0	1	0	1
N27	*Cobetia amphilecti*	1	0	0	1	0	1	1	0	0	1	0	1
N28.1	*Pseudomonas sp.*	0	0	0	0	1	0	1	0	0	0	0	0
N28.2	*Pseudomonas sp.*	0	0	0	1	1	0	0	1	0	0	0	0
N29	*Cellulophaga algicola*	0	0	0	1	0	0	0	1	0	1	0	0
N30	*Cobetia amphilecti*	1	0	0	1	0	1	0	0	0	1	0	0
N31	*Cobetia litoralis*	1	0	0	0	1	1	0	0	0	1	0	0
N32	*Cobetia amphilecti*	1	0	0	1	1	0	1	0	0	1	0	1
N33	*Cellulophaga pacifica*	0	0	0	1	1	0	0	0	0	1	0	0
N34	*Rothia sp.*	0	0	0	1	0	0	1	0	0	1	0	0
N36	*Staphylococcus sp.*	1	0	0	0	0	0	0	0	0	1	0	0
N37.1	*Bacillus licheniformis*	0	0	0	1	1	0	0	0	0	0	0	0
N37.2	*Bacillus licheniformis*	0	0	0	1	1	0	0	0	0	1	0	0
N38	*Staphylococcus sp.*	1	0	0	0	1	0	0	0	0	1	0	0

N39.1	*Pseudomonas oryzihabitans*	1	0	0	1	1	0	0	0	0	0	0	0
N39.2	*Pseudomonas oryzihabitans*	0	0	0	1	1	0	0	0	0	0	0	0
N40.1	*Cellulophaga pacifica*	1	0	0	1	1	0	1	0	0	1	0	0
N40.2	*Cellulophaga pacifica*	1	0	0	1	0	0	1	0	0	1	0	0
N41.1	*Staphylococcus sp.*	1	0	0	1	1	0	0	0	0	1	0	0
